# Structure-based redesign enables clinical translation of CAIX-targeted theranostics in clear cell renal cell carcinoma

**DOI:** 10.7150/thno.134970

**Published:** 2026-07-20

**Authors:** Zexin Xu, Hui Zhou, Ye Dong, Dan Feng, Baocheng Chen, Hongxin Li, Ruitao Yang, Ningjie Li, Yuting Cao, Hankuan He, Han Wu, Yuhua Zhong, Hubing Wu, Liang Zhao, Haojun Chen, Kongzhen Hu

**Affiliations:** 1 Department of Nuclear Medicine, Nanfang Hospital, Southern Medical University, 1838 Guangzhou North Road, Guangzhou, Guangdong Province, 510515, China.; 2 School of Pharmaceutical Sciences, Southern Medical University, 1838 Guangzhou North Road, Guangzhou, 510515, China.; 3 Department of Nuclear Medicine and Minnan PET Center, The First Affiliated Hospital of Xiamen University, School of Medicine, Xiamen University, Xiamen, 361003, China.; 4 Department of Rehabilitation Medicine, Nanfang Hospital, Southern Medical University, 1838 Guangzhou North Road, Guangzhou, Guangdong Province, 510515, China.

**Keywords:** clear cell renal cell carcinoma, carbonic anhydrase IX, PET/CT imaging, theranostic pair, ^68^Ga/^177^Lu-ZH2

## Abstract

Carbonic anhydrase IX (CAIX) is an interesting therapeutic target in clear cell renal cell carcinoma (ccRCC), but gastrointestinal uptake impedes the clinical translation of CAIX-targeted radioligand. In this study, structure-guided optimization of a cyclic peptide scaffold was conducted to decouple tumor targeting from gastrointestinal retention.

**Methods:**

Five novel CAIX-targeting ligands were developed at the base of a cyclic peptide and evaluated in OS-RC-2 cell, small animal positron emission tomography/computed tomography (PET/CT), biodistribution, and radiotherapy experiments. The lead candidate was tested on 21 patients with cancer in comparison to ^18^F-FDG.

**Results:**

In preclinical experiments, ⁶⁸Ga/¹⁷⁷Lu-ZH2 exhibited sub-nanomolar CAIX affinity and a favorable pharmacokinetics, with preserved tumor uptake and markedly reduced gastrointestinal retention, compared with benchmark ⁶⁸Ga/¹⁷⁷Lu-DPI-4452. In mice, ¹⁷⁷Lu-ZH2 inhibited tumor growth and prolonged survival without evident toxicity. In a first-in-human study of 21 patients with renal masses, ⁶⁸Ga-ZH2 PET/CT was deemed safe and demonstrated a superior diagnostic performance to ¹⁸F-FDG, detecting additional primary tumors and metastases with higher contrast.

**Conclusions:**

These results establish ^68^Ga/^177^Lu-ZH2 as a CAIX-targeted imaging and potential therapeutic agent with favorable preclinical and early clinical imaging characteristics, enabling the sensitive detection of ccRCC and laying the groundwork for further therapeutic investigation.

## Introduction

Clear cell renal cell carcinoma (ccRCC) is the most prevalent subtype of renal cancer, accounting for 70% to 80% of all renal malignancies; it results in 170,000 annual deaths worldwide 1, 2. Despite substantial advances in renal cancer treatment, the clinical management of ccRCC remains challenging, particularly for early diagnosis, accurate staging, and treatment of advanced stages. Although localized ccRCC can often be managed with surgical resection, a substantial number of patients present with or eventually develop metastases, which have been associated with poor prognosis and low 5-year survival rates 3, 4.

Radiotheranostics has emerged as a powerful precision-medicine approach that systematically integrates biomarker-targeted imaging with therapy by using the identical or structurally similar ligands labeled with diagnostic or therapeutic radionuclides 5, 6. This strategy successfully treats prostate cancer by using prostate-specific membrane antigen (PSMA)-targeted radioligands 7, 8. Carbonic anhydrase IX (CAIX) is an interesting therapeutic target in ccRCC because of its near-ubiquitous overexpression in this tumor type, driven by von Hippel–Lindau gene loss and hypoxia-inducible signaling 9-12. In contrast, the physiological expression of CAIX in healthy human tissues is low and largely restricted to the gastrointestinal epithelium 13, 14. These features have positioned CAIX as one of the most extensively investigated biomarkers for ccRCC. Multiple CAIX-targeting strategies have been explored, including monoclonal antibodies and small-molecule inhibitors 5. Radiolabeled anti-CAIX antibodies, including girentuximab, demonstrate specific tumor-targeting properties but have prolonged circulation and unfavorable dosimetry profiles, which limit their therapeutic utility 15, 16. Alternatively, small-molecule CAIX inhibitors have faster clearance rates and improved imaging kinetics; nonetheless, they exhibit insufficient tumor uptake or unfavorable tumor-to-kidney ratios, which is particularly problematic in renal malignancies 17-21. Cyclic peptide-based ligands achieve a promising balance among affinity, pharmacokinetics, and manufacturability. Of them, the theranostic agent ⁶⁸Ga/¹⁷⁷Lu-DPI-4452 has demonstrated favorable tumor uptake and enabled both positron emission tomography (PET) imaging and radioligand therapy in preclinical and early clinical studies 22, 23. However, its clinical translation is limited by prominent gastrointestinal retention, which may obscure abdominal lesions and increase toxicity during therapy. Therefore, a systematic strategy to decouple tumor-targeting from gastrointestinal CAIX binding is warranted for the clinical translation of CAIX-directed theranostics.

**Figure 1:**
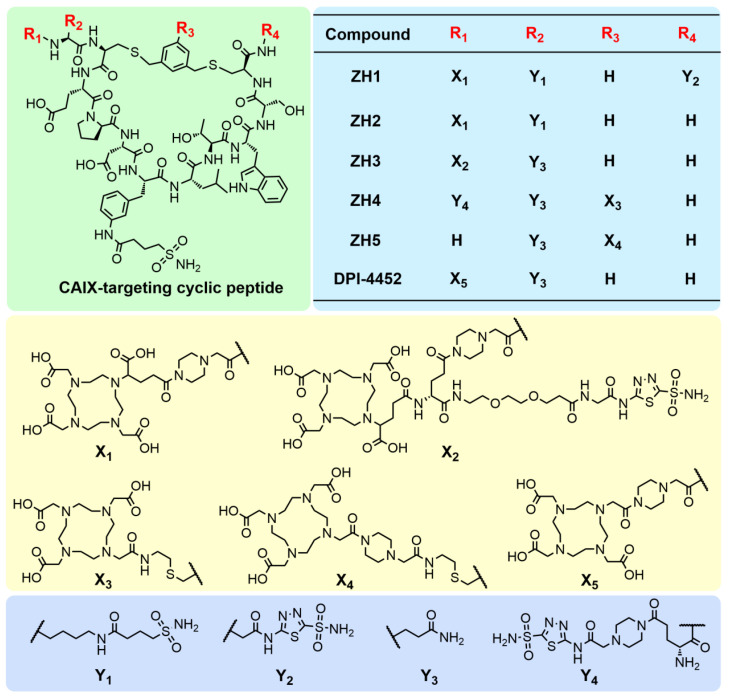
** The chemical structure of ZH1–5**.

In this study, we addressed this translational bottleneck through structure-guided optimization of a cyclic peptide scaffold. A series of novel CAIX-targeting ligands, termed ZH1–5, was generated by systematically modifying the CAIX-binding motifs, linker composition, and chelator architecture (**Figure 1**). Comprehensive preclinical evaluation against the parent ligand DPI-4452 identified ZH2 as the lead candidate, exhibiting preserved tumor uptake and markedly reduced background retention, particularly in the gastrointestinal tract. Accordingly, ZH2 was developed as a matched theranostic pair labeled with ^68^Ga for PET imaging and ^177^Lu for targeted radioligand therapy. Thus, ⁶⁸Ga-ZH2 was advanced to preliminary clinical evaluation in patients with ccRCC.

## Materials and Methods

### Study design

To separate tumor uptake from gastrointestinal retention, this study aimed to develop and translate a novel, therapeutically viable CAIX-targeted theranostic agent. By altering the CAIX-binding motifs, linker composition, and chelator architecture on a cyclic peptide scaffold, a series of novel ligands was systematically generated. *In vitro*/*vivo* and PET/CT imaging were used to screen these ligands for the optimal affinity, pharmacokinetics, biodistribution, and tumor-specific uptake when compared with ⁶⁸Ga-DPI-4452. The organ retention, tumor uptake, antitumor efficacy, and organ toxicity of the selected ligand were determined in the OS-RC-2 xenograft, compared with ^177^Lu-labeling DPI-4452. Based on favorable preclinical data, the diagnostic ligand was translated into a prospective, exploratory human study. The statistical methods and replicates are listed below and in the figure legends. The investigators were not blinded for these experiments.

### Sex as a biological variable

This study involved 21 patients, consisting of 15 men and 6 women. The animal experiments exclusively involved male mice. It is unknown whether the findings apply to female mice. Sex was not considered a biological variable.

### Ethics approval and consent to participate

The research protocol was approved by the First Affiliated Hospital of Xiamen University Institutional Review Board (NCT06956144), and written informed consent was obtained from all participants. The study procedures were conducted in accordance with the tenets of the Declaration of Helsinki. All participants were informed about the purpose of the study and assured of confidentiality; they provided written consent before participation. Participation was voluntary, and the respondents could withdraw at any time without consequence. All animal experiments were approved by the Animal Ethics Committee of Nanfang Hospital and conducted at Southern Medical University (IACUC-LAC-20250211-005).

### Chemistry

All solvents and reagents were purchased from commercial suppliers and used exactly as supplied unless otherwise stated. The linear peptide intermediates of ZH1 and ZH2–ZH5 were synthesized on CTC resin and Rink amide MBHA resin, respectively, using standard Fmoc-based solid-phase peptide synthesis. The corresponding target ligands, ZH1–ZH5, were produced by cyclization and other modifications. Crude products were purified through preparative high-performance liquid chromatography (HPLC), and mass spectrometry was used to verify each ligand’s identity. Analytical HPLC demonstrated that the final products had a chemical purity of > 95%.

### Radiolabeling

First, 2.0 mL of 0.1 M HCl was used to elute ⁶⁸GaCl₃ from a ⁶⁸Ge/⁶⁸Ga generator (Chengdu New Radiomedicine Technology Co., Ltd.). An aliquot of the eluate (2.0 mL; 0.1 M HCl; 740–1110 MBq) was mixed with 1.0 mL of 2.0 M HEPES buffer containing 10 nmol of the precursor, and the pH was adjusted to 4.0. For 10 min, the reaction mixture was incubated at 105 ℃. After cooling, the mixture was diluted with 5 mL of water and loaded onto a C18 cartridge (Waters, WAT024501). The cartridge was washed with 10 mL of sterile water, and the product was respectively eluted with 1 mL of ethanol/water (1:1, v/v) and 7 mL of normal saline into a sterile evacuated vial (BJPET, BJPET-501010). During ¹⁷⁷Lu labeling, 120 MBq of ¹⁷⁷LuCl₃ was diluted with 0.16 mL of NaOAc (0.4 M, pH 5.5), and the precursor (20 nmol) and 2.0 mg of gentisic acid were added. The reaction mixture was incubated at 95 ℃ for 30 min; it was subjected to quality control and purification by radio-HPLC. The detailed radio-HPLC method is described in the Supplementary Material.

### Stability and partition coefficient study

For stability testing, radiotracers were incubated at 37 °C for 2 h (⁶⁸Ga-labeled ligands) or 24 h (¹⁷⁷Lu-labeled ligands) in phosphate-buffered saline (PBS, pH 7.4), mouse serum, or human serum (200 μL each). To determine radiochemical purity, samples were run through a 0.22-μm syringe filter and analyzed by radio-HPLC. The radiotracer was added to a biphasic mixture of n-octanol and PBS (1:1, v/v; total volume 10 mL) to calculate the distribution coefficient (logD). After vigorous mixing for 5 min at room temperature, the samples were centrifuged at 2,000 rpm for 5 min. A γ-counter (CAPRAC-R; Capintec, Ramsey, NJ, USA) was used to quantify radioactivity in 300 μL aliquots from each phase. LogD was calculated as the logarithm of the ratio of the counts in the octanol phase to those in the aqueous phase.

### Cells and animals

CAIX-positive OS-RC-2 human renal cell carcinoma cells were obtained from RRID (CVCL_1626), and CAIX-negative AsPC-1 human pancreatic cancer cells were obtained from RRID (CVCL_0152). CAIX-positive OS-RC-2 cells and CAIX-negative AsPC-1 cells were cultured in RPMI-1640 medium supplemented with 10% fetal bovine serum and 1% penicillin–streptomycin. Cells were maintained in a humidified atmosphere containing 5% CO₂ at 37 °C. For xenograft models, OS-RC-2 or AsPC-1 cells (6 × 10⁶ cells in 100 μL PBS) were subcutaneously injected into the right flank of male BALB/c nude mice (4–6 weeks old, 20–25 g; Gempharmatech-GD, Guangdong, China). Imaging and biodistribution experiments were initiated when tumors reached 4 to 7 mm in diameter. Male C57BL/6 mice (4–6 weeks old, 20–25 g; Zhuhai BesTest Bio-Tech, Guangdong, China) were used for toxicity assessments.

### Cell experiments

For competitive binding assays, using ¹⁷⁷Lu-DPI-4452 as the radioligand, OS-RC-2 cells were co-incubated with unlabeled ligands (10⁻¹²–10⁻⁵ M) for 1 h at 37 ℃. GraphPad Prism (version 9.5.0) was used to compute the half-maximal inhibitory concentration values. For uptake assays, OS-RC-2 cells were treated with either ⁶⁸Ga- or ¹⁷⁷Lu-labeled ligands for 10, 30, 60, and 120 min (and 1440 min for ¹⁷⁷Lu-labeled ligands). After two PBS washes and treatment with lysis buffer (1 M NaOH, 0.2% SDS), radioactivity was measured using a γ-counter. For internalization assays, after incubation with ¹⁷⁷Lu-labeled ligands (10–1440 min), cells were washed and treated with glycine–HCl buffer (1 M, pH 2.2) for 10 min at 37 °C to remove membrane-bound activity. As mentioned previously, cells were washed and lysed. Radioactivity was measured separately in the glycine–HCl fraction (surface-bound) and lysate (internalized). For efflux assays, the cells were pre-incubated with radiotracers for 1 h before being washed and incubated in fresh RPMI-1640 medium for 0, 10, 30, 60, 120 and 1440 min. After incubation, cells were washed and lysed; radioactivity was quantified. For blocking assays, ⁶⁸Ga-ZH2 or ¹⁷⁷Lu-ZH2 was co-incubated with DPI-4452 (0.01 mmol/L) in OS-RC-2 cells for 1 h. For CAIX-negative controls, ⁶⁸Ga-ZH2 or ¹⁷⁷Lu-ZH2 was incubated with AsPC-1 cells for 1 h. In all experiments, a γ-counter was used to measure radioactivity, which is expressed as the percentage of the injected dose per million cells (%ID/1 mio cells).

### Western blot experiment

Phenylmethylsulfonyl fluoride (1 mM) and protease inhibitors were added to the lysis buffer to lyse the tissues. Protein concentrations were determined using a BCA assay. SDS-PAGE was used to separate equal amounts of protein, which were transferred to PVDF membranes and blocked with 5% skim milk for 2 h at room temperature. The membranes were incubated with a primary antibody against CAIX and an internal control antibody (β-tubulin) overnight at 4 °C. Subsequently, they were treated with an HRP-conjugated secondary antibody for 1 h at room temperature. Signals were detected using enhanced chemiluminescence and quantified using ImageJ (NIH).

### Small-animal PET/CT imaging

PET/CT imaging of OS-RC-2 and AsPC-1 xenografts was conducted on an Inveon micro-PET/CT scanner (Siemens, Erlangen, Germany). Immediately after the infusion of ⁶⁸Ga-labeled ligands (7.4 MBq in 150 μL saline) into the tail vein, dynamic PET/CT images were acquired for 2 h. Static PET/CT imaging was conducted at 1 h after injection. For blocking experiments, OS-RC-2 xenografted mice were co-injected with ⁶⁸Ga-ZH2 (7.4 MBq) and DPI-4452 (47.5 nmol). A 3D ordered-subsets expectation maximization (3D-OSEM) algorithm was used to reconstruct the images, which were quantified as the percentage injected dose per gram (%ID/g) and analyzed using Inveon Research Workplace (version 4.1). CT-based attenuation correction was applied. Regions of interest were plotted over tumors and major organs.

### Biodistribution and radioligand therapy study

Radiotracers (0.74 MBq in 150 μL saline; *n* = 3–4 per group) were injected via the tail vein for biodistribution studies. For ⁶⁸Ga-labeled ligands, mice were euthanized at 1 h post-injection (p.i.). For ¹⁷⁷Lu-labeled ligands, mice were euthanized at 1, 4, 24, 72, and 168 h p.i. For blocking studies, OS-RC-2 mice were co-injected with ⁶⁸Ga-ZH2 and DPI-4452 (47.5 nmol) and euthanized at 1 h. For CAIX-negative controls, AsPC-1 xenografted mice were injected with ⁶⁸Ga-ZH2 alone. A γ-counter was used to harvest, weigh, and count the organs. Data are expressed as %ID/g. Mice were randomly assigned to three groups (*n* = 7/group) when OS-RC-2 tumors reached 133 ± 26 mm³: (i) saline, (ii) ¹⁷⁷Lu-ZH2, and (iii) ¹⁷⁷Lu-DPI-4452. The treatment groups received a single intravenous injection of 37 MBq of ¹⁷⁷Lu-labeled ligand (molar activity 37 MBq/nmol), and the control group received an equal volume of saline. The tumor size and body weight were measured every 2 days from days 0 to 24. The tumor volume was calculated as follows: (length × width²)/2. The relative body weight was calculated as follows: body weight on day X/body weight on day 0. Humane endpoints included the tumor volume > 1,500 mm³, body weight loss > 15%, or death. At study completion, the major organs were collected for histological evaluation by Hematoxylin-eosin staining.

### Toxicity test

C57BL/6 mice (18–22 g) were randomized into two groups, namely the treatment and control groups (*n* = 10 per group). The treatment group received a single tail vein injection of ^68^Ga-ZH2, approximately 70 MBq in 0.5 mL saline, whereas the control group received an equivalent volume of saline*.* Body weight was recorded daily for 7 days, and the mice were monitored for clinical signs of toxicity.

### Inclusion criteria for patients

The purpose of this prospective, single-center diagnostic study was to evaluate the performance of ^68^Ga-ZH2 PET/CT in patients with primary or metastatic ccRCC. Twenty-one consecutive patients were enrolled between June and November 2025. Nineteen were treatment-naïve individuals with suspicious renal masses, and two were under surveillance for suspected ccRCC recurrence following prior nephrectomy. Histopathology was used as the reference standard to confirm all lesions. Primary tumors were confirmed through surgical histopathology or core needle biopsy. Metastatic lesions were confirmed through histopathology, where tissue was available, or through composite assessment by integrating morphological imaging (contrast-enhanced CT/MRI), clinical history, and imaging follow-up.

### Clinical PET/CT acquisition

Whole-body PET/CT was conducted using a hybrid PET/CT scanner (Discovery MI, GE HealthCare). A low-dose CT (120 kV; automated tube current modulation, 64–321 mA; pitch 0.8) was acquired for attenuation correction and anatomical localization, followed by a PET scan in the 3D acquisition mode with 6 to 8 bed positions and 2.0 to 2.5 min per position, conducted 1 hour after injection. PET data were reconstructed using the Bayesian penalized likelihood reconstruction algorithm (Q.Clear, GE Healthcare), with a penalization factor (β) of 500 and CT-based attenuation correction. All fused PET/CT images were reviewed using the MedEx system (MedEx Technology Limited Corporation) for registration, fusion, and quantitative measurement. Each patient received ^68^Ga-ZH2 (mean activity 156.14 MBq, range 72.89–263.07 MBq), followed by PET/CT imaging, at 1 h p.i. Nineteen patients further underwent paired ^18^F-FDG PET/CT within 1 week, using a weight-adjusted dose of 3.7 MBq/kg. All 21 patients were followed up for 72 h after radiotracer injection, and no drug-related adverse events were observed during this period.

### Clinical PET/CT analysis

Two experienced nuclear medicine physicians independently reviewed PET/CT images while being blinded to clinical and pathological data. Discrepancies were resolved by consensus. After eliminating sites with known physiological uptake, lesions were classified as positive on PET/CT if focal radiotracer uptake was visibly greater than that of the surrounding healthy tissue. According to RECIST 1.1 criteria, lesion measurability was assessed using the short-axis diameter for lymph nodes and the long-axis diameter for extranodal lesions. A lesion was considered measurable if it had the longest diameter ≥10 mm (extranodal) or the short-axis ≥15 mm (lymph nodes). For all detected lesions, a volumetric region of interest (ROI) was manually delineated on the axial PET slice showing the highest radiotracer concentration to record the maximum standardized uptake value (SUVmax). The positive lesions were taken into consideration when PET imaging showed the non-physiologic foci of increased radiotracer uptake (excluding inflammation, trauma, bone degeneration, and other benign lesions). Reference ROIs were positioned in the gluteus maximus muscle and the mediastinal blood pool to obtain quantitative ratios. The tumor-to-mediastinum (SUV_T/M_) and tumor-to-gluteus (SUV_T/G_) ratios were calculated as follows:

SUV_T/M_ = SUVmax (lesion) / SUVmax (mediastinum)

SUV_T/G_ = SUVmax (lesion) / SUVmax (gluteus maximus)

SUVmax, SUV_T/M_, and SUV_T/G_ were compared between ⁶⁸Ga-ZH2 and ¹⁸F-FDG.

### Statistics

Statistical analyses were conducted using Origin (version 2024; OriginLab Corporation, Northampton, MA, USA) and GraphPad Prism (version 9.5.0; GraphPad Software, Boston, MA, USA). Data are presented as the mean ± standard deviation (SD). One-way analysis of variance (ANOVA) and paired or unpaired two-tailed *t* test were used to determine statistical significance. Survival curves were computed via the Kaplan–Meier method, and differences among groups were demonstrated by log-rank testing. A* P* value < 0.05 was considered statistically significant.

## Results

### Chemistry and radiolabeling

All the five CAIX-targeting ligands (ZH1–5) were successfully synthesized via solid-phase peptide synthesis, with chemical purities > 95%. The synthetic route is illustrated in **Schemes S1–S5**. Intermediates and final products were characterized using mass spectrometry and HPLC, with corresponding spectroscopic and chromatographic data provided in the Supplementary Material. All ligands were radiolabeled with ^68^Ga, demonstrating non-decay-corrected radiochemical yields > 60%, radiochemical purities (RCPs) > 98%, and molar activities > 25 GBq/μmol. Additionally, ^177^Lu-ZH1–3 and ^177^Lu-DPI-4452 were synthesized at 95 ℃ for 30 min, with RCP values > 98% (**Figure S1**). For *in vivo* biodistribution and therapy studies, ^177^Lu-labeled ligands were used directly at a molar activity of 37 MBq/nmol. In contrast, cellular and competitive binding assays used no-carrier-added formulations that were purified through reverse-phase HPLC and exhibited higher molar activities than those used for *in vivo* studies.

### Radioligand stability and distribution coefficient

The CAIX-targeting ligands radiolabeled with either ^68^Ga or ^177^Lu exhibited high stability under various conditions (**Figure S2–S3**). All ^68^Ga-labeled ligands remained stable in PBS, mouse serum, and human serum over 2 h of incubation. Similarly, ^177^Lu-ZH2 and ^177^Lu-DPI-4452 demonstrated comparable stability in the media during 24 h of incubation, with > 95% of the ligands remaining intact. The radioligands had the following partition coefficients (logD): ^68^Ga-ZH1, -3.53 ± 0.08; ^68^Ga-ZH2, -4.25 ± 0.05; ^68^Ga-ZH3, -3.82 ± 0.03; ^68^Ga-ZH4, -3.12 ± 0.05; ^68^Ga-ZH5, -3.87 ± 0.07; ^68^Ga-DPI-4452, -3.66 ± 0.08; ^177^Lu-ZH2, -2.98 ± 0.06; and ^177^Lu-DPI-4452, -2.47 ± 0.04. Consistently negative logD values indicate the highly hydrophilic character of these radioligands.

### *In vitro* cellular experiments

**Figure 2:**
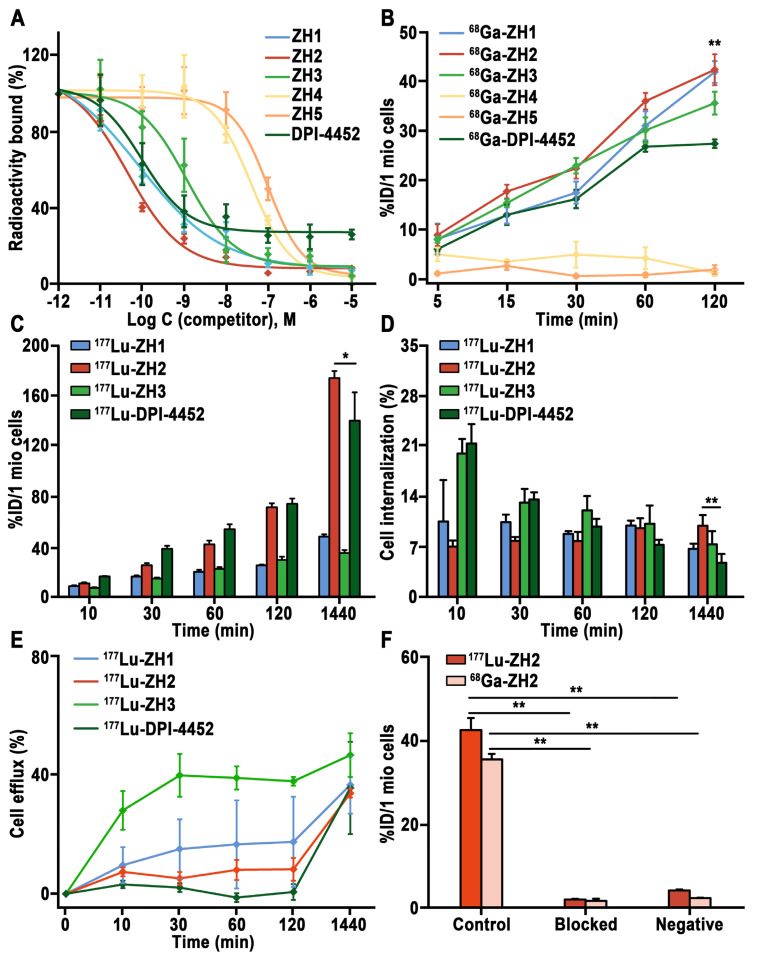
***In vitro* characterization of CAIX-targeting radioligands.** (**A**) Competitive binding of ^177^Lu-DPI-4452 against unlabeled ligands (10⁻^12^–10⁻^5^ M) in OS-RC-2 cells. (**B**) Time-dependent uptake of ^68^Ga-labeled ligands over 120 min in OS-RC-2 cells. (**C**) Uptake of ^177^Lu-ZH1–3 and ^177^Lu-DPI-4452 over 1440 min in OS-RC-2 cells. (**D**) Internalization kinetics of ^177^Lu-labeled ligands over 1440 min in OS-RC-2 cells. (**E**) Efflux kinetics after 1 h incubation with ^68^Ga-labeled ligands and subsequent chase with fresh RPMI-1640 medium in OS-RC-2 cells. (**F**) CAIX specificity of ^68^Ga-ZH2 or ^177^Lu-ZH2 in OS-RC-2 cells (CAIX-positive) with or without DPI-4452, and in AsPC-1 (CAIX-negative) cells. Data represent %ID/1 mio cells (mean ± SD, *n* = 3–4). *P < 0.05, **P < 0.001.

Competitive cell-binding assays were conducted to evaluate the binding affinities of the unlabeled probes (**Figure 2A**). The half-maximal inhibitory concentration values for ZH1–5 and DPI-4452 were 0.10 ± 0.04, 0.03 ± 0.02, 1.10 ± 0.99, 42.29 ± 6.81, 95.59 ± 16.20, and 0.10 ± 0.02 nM, respectively. Notably, ZH1 and ZH2 exhibited binding affinities comparable to that of the reference ligand DPI-4452, whereas ZH3–5 showed a substantial decrease in affinity. Cellular uptake of the ^68^Ga-labeled ligands was subsequently evaluated (**Figure 2B**). The uptake of ^68^Ga-ZH1–3 and ^68^Ga-DPI-4452 was markedly higher than that of ^68^Ga-ZH4 and ^68^Ga-ZH5, increasing in a time-dependent manner and peaking at 120 min. At this time point, the uptake of ^68^Ga-ZH1–3 was significantly higher than that of ^68^Ga-DPI-4452 (all *P* < 0.001). A similar time-dependent uptake trend was observed for the ^177^Lu-labeled ligands, ^177^Lu-ZH1–3 and ^177^Lu-DPI-4452 (**Figure 2C**). After 1440 min, ^177^Lu-ZH2 displayed the highest cellular uptake among all ^177^Lu-labeled ligands (173.82 ± 5.57% ID/1 mio cells, *P* < 0.05). Cell-internalization experiments revealed distinct patterns among the candidates (**Figure 2D**). The internalization of ^177^Lu-ZH1, ^177^Lu-ZH3, and ^177^Lu-DPI-4452 gradually decreased over 1440 min. In contrast, ^177^Lu-ZH2 exhibited continuously increasing internalization, resulting in a significantly higher level than that of ^177^Lu-DPI-4452 at 1440 min (9.89 ± 1.48% vs. 4.74 ± 1.23%, *P* < 0.001). Cell-efflux experiments revealed slow clearance for ^177^Lu-ZH1, ^177^Lu-ZH2, and ^177^Lu-DPI-4452, with > 63% of the activity remaining intracellularly after 1440 min (**Figure 2E**). Conversely, ^177^Lu-ZH3 was cleared rapidly, with only 60% retention observed as early as 30 min. In addition, the uptake of both ^68^Ga-ZH2 and ^177^Lu-ZH2 (< 2.5% ID/1 mio cells, *P* < 0.001) was significantly reduced in OS-RC-2 cells upon co-incubation with the competitor DPI-4452 and in AsPC-1 cells (negative group; < 4.5% ID/1 mio cells, *P* < 0.001), confirming the CAIX-specific binding of ZH2 (**Figure 2F**).

### Small-animal PET/CT imaging and ^68^Ga-radioligand biodistribution

**Figure 3:**
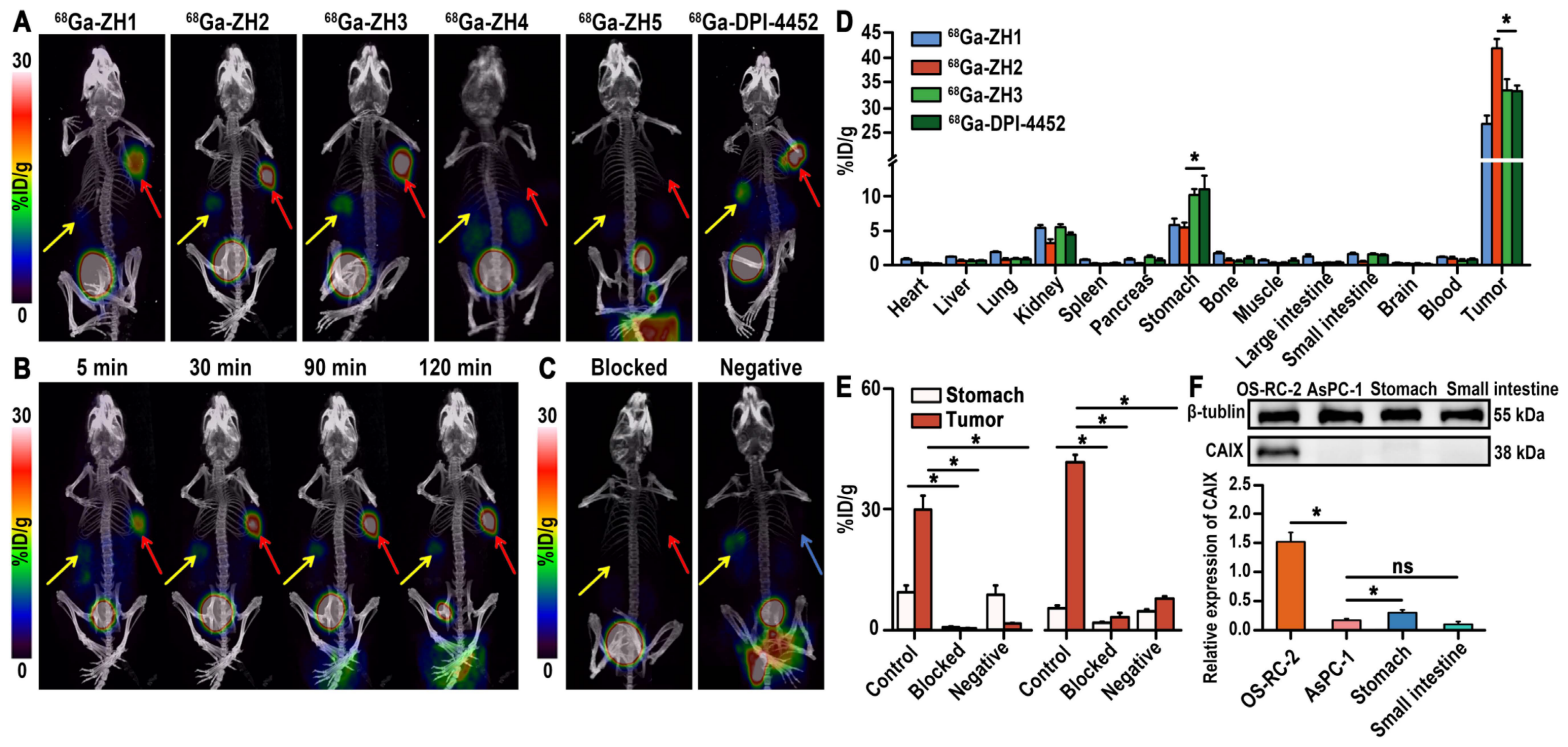
**Representative maximum intensity projections from PET/CT imaging and biodistribution analyses with ^68^Ga-radioligands.** (**A**) PET/CT images of ^68^Ga-ZH1–5 and ^68^Ga-DPI-4452 at 1 h post-injection (p.i.) in OS-RC-2 (CAIX-positive) xenografts. (**B**) PET/CT images of OS-RC-2 xenografts acquired within 120 min after injection of ^68^Ga-ZH2. (**C**) PET/CT images of OS-RC-2 and AsPC-1 (CAIX-negative) xenografts at 1 h p.i. after co-injection of ^68^Ga-ZH2 with or without DPI-4452 as a competitor. (**D**) Biodistribution in OS-RC-2 xenografts at 1 h p.i. after injection of ^68^Ga-ZH1–3 and ^68^Ga-DPI-4452. (**E**) Uptake in stomach and tumor at 1 h p.i. after co-injection of ^68^Ga-ZH2 with or without DPI-4452 in small-animal PET imaging (left) and biodistribution studies (right). (**F**) Western blot analysis of tumors and selected organs. Red and blue arrows indicate OS-RC-2 and AsPC-1 tumors, respectively; the yellow arrow indicates the stomach. Results are presented as the mean ± SD (*n* = 3–4), *P < 0.05; ns, not significant (*P* > 0.05).

To characterize the pharmacokinetic behavior of the CAIX-targeting radioligands *in vivo*, dynamic small-animal PET/CT imaging and time-activity curve analyses were performed over a 120 min acquisition period in nude mice bearing OS-RC-2 xenografts (**Figure 3 and Figure S4**). All tracers were predominantly cleared through the renal pathway, as evidenced by an early renal uptake peak within the first 5 min p.i., followed by rapid washout and progressive accumulation of radioactivity in the bladder. Representative maximum-intensity-projection images revealed pronounced tumor and stomach uptake for ^68^Ga-ZH1–3 and the reference ligand ^68^Ga-DPI-4452 (**Figure 3A**). In contrast, ^68^Ga-ZH4–5 exhibited minimal retention in both tumor and stomach, consistent with their low CAIX-binding affinities observed *in vitro*. Semi-quantitative analysis of PET/CT at 120 min p.i. demonstrated that ^68^Ga-ZH2 and ^68^Ga-DPI-4452 exhibited comparable tumor uptake (33.49 ± 2.98 vs. 28.64 ± 5.51 %ID/g) and renal uptake (1.91 ± 0.21 vs. 2.67 ± 0.63 %ID/g), but ^68^Ga-ZH2 showed significantly reduced stomach uptake (7.94 ± 0.17 vs. 10.87 ± 2.84 %ID/g, *P* < 0.05) (**Figure S4**). Based on this favorable balance between preserved tumor-targeting and reduced gastrointestinal accumulation features, ^68^Ga-ZH2 was selected as the lead candidate for further evaluation. Time-course PET imaging confirmed rapid and sustained accumulation of ^68^Ga-ZH2 in OS-RC-2 tumors, enabling clear tumor visualization from 5 to 120 min p.i. (**Figure 3B**). The CAIX specificity of ^68^Ga-ZH2 was further supported by competitive blocking and CAIX-negative control experiments, which showed significantly reduced tumor uptake under both conditions (**Figure 3C**).

To validate the small-animal PET imaging experiments, biodistribution studies of ^68^Ga-ZH1–3 and ^68^Ga-DPI-4452 were performed at 1 h p.i. in OS-RC-2 and AsPC-1 xenograft models. As shown in **Figure 3D**, all four ligands exhibited high uptake in CAIX-positive OS-RC-2 tumors, followed by substantial accumulation in the stomach. Moderate renal uptake was observed, likely reflecting renal excretion, whereas the uptake remained low in other organs (< 2 %ID/g). These biodistribution results are consistent with the observations from small-animal PET imaging (**Table S1**). Among the ligands, ^68^Ga-ZH2 demonstrated the highest tumor uptake and the lowest stomach uptake, with statistically significant differences compared with the values corresponding to the control agent ^68^Ga-DPI-4452 (tumor, 41.84 ± 1.82 vs. 33.32 ± 1.13 %ID/g, *P* < 0.001; stomach, 5.51 ± 0.73 vs. 11.11 ± 1.98 %ID/g, *P* < 0.001). In addition, ^68^Ga-ZH2 showed superior tumor-to-background contrast ratios in **Table S2** across multiple organs—including liver, lung, kidney, spleen, pancreas, bone, muscle, large intestine, and small intestine—indicating an overall improvement in pharmacokinetic performance than that of the reference tracer. CAIX-specific binding was further confirmed by competitive blocking with excess unlabeled DPI-4452 (**Figure 3E**), which markedly reduced both tumor and stomach uptake in PET imaging and biodistribution analyses (*P* < 0.001). Furthermore, the tumor uptake of ^68^Ga-ZH2 was substantially lower in the CAIX-negative AsPC-1 xenografts than in OS-RC-2 models, whereas physiological stomach uptake remained comparable, collectively confirming CAIX-dependent tumor-targeting. Finally, western blotting corroborated the imaging and biodistribution results and showed specific CAIX expression in OS-RC-2 tumors and stomach, consistent with previous reports of physiological CAIX expression in the gastrointestinal tract (**Figure 3F and Figure S5**) 9.

### Biodistribution characteristics and therapeutic efficacy

**Figure 4:**
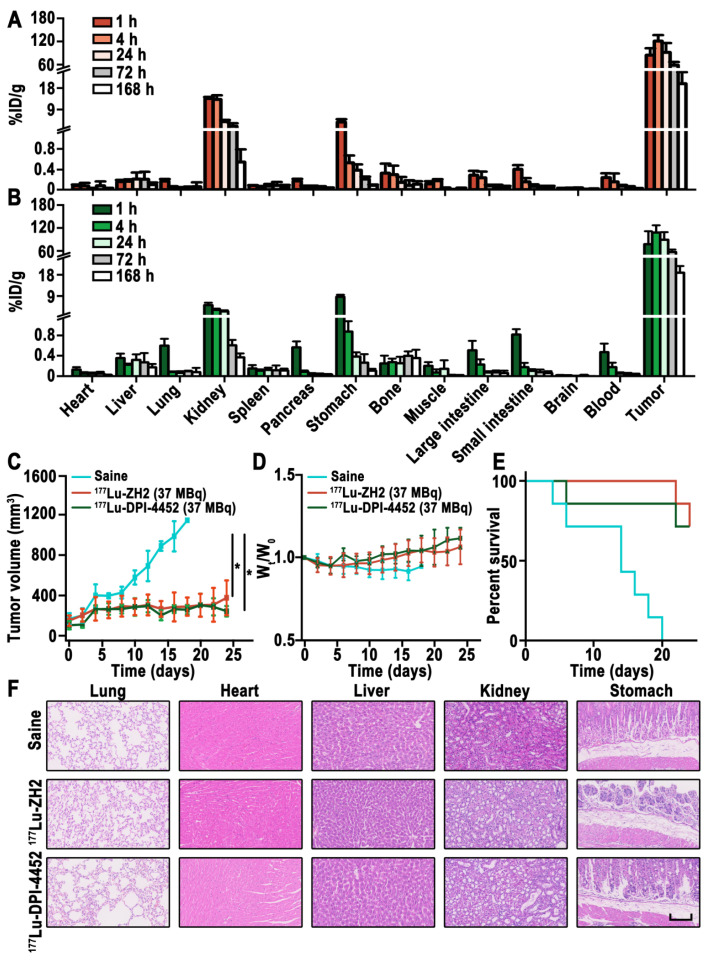
***Ex vivo* biodistribution, therapeutic efficacy, and toxicity evaluation of ^177^Lu-ZH2 and ^177^Lu-DPI-4452 in OS-RC-2 xenografts.** *Ex vivo* biodistribution in OS-RC-2 xenografts after injection of ^177^Lu-ZH2 (**A**) and ^177^Lu-DPI-4452 (**B**) over 168 h. Curves show changes in the average tumor size (**C**), average body weight (**D**), and survival curve (**E**) over time. (**F**) Hematoxylin-eosin staining of major organ sections (including lung, heart, liver, kidney, and stomach) from different groups. Results are presented as the mean ± SD (biodistribution: *n* = 3–4; therapy study: *n* = 7). Scale bar: 50 μm, *P < 0.01.

Based on the favorable *in vitro* and *in vivo* performance of ZH2, the extended-time biodistribution of ^177^Lu-ZH2 was next assessed and compared with ^177^Lu-DPI-4452 in OS-RC-2 xenograft models (**Figure 4A–B, and Table S3–S4**). Both radioligands exhibited high and sustained tumor uptake. Tumor activity for ^177^Lu-ZH2 and ^177^Lu-DPI-4452 was 83.51 ± 18.23 and 77.63 ± 33.78 %ID/g at 1 h p.i., peaked at 4 h (120.51 ± 15.15 and 107.92 ± 18.86 %ID/g, respectively), and gradually decreased but remained substantial at 168 h (19.79 ± 4.79 and 18.76 ± 2.83 %ID/g, respectively). Moderate kidney and stomach uptake was observed at 1 h, followed by progressive clearance; stomach activity declined to < 1.0 %ID/g by 4 h. Uptake in other organs remained low (< 1.0 %ID/g) from 1 h onward. To compare overall tissue exposure, areas under the curve (AUC) were calculated from biodistribution data (**Table S5**). Tumor AUCs were comparable between ^177^Lu-ZH2 and ^177^Lu-DPI-4452 (9686.00 ± 1064.00 vs. 9440.00 ± 810.20 (%ID/g)·h). In contrast, renal AUC was higher for ^177^Lu-ZH2 (481.30 ± 86.40 (%ID/g)·h) than for ^177^Lu-DPI-4452 (183.90 ± 25.24 (%ID/g)·h), whereas stomach AUC for ^177^Lu-ZH2 was approximately 1.4-fold lower than that of ^177^Lu-DPI-4452. Accordingly, the tumor-to-kidney AUC ratio for ^177^Lu-ZH2 was approximately 20, and tumor-to-healthy tissue AUC ratios exceeded 200 for multiple non-renal organs.

To evaluate therapeutic efficacy, OS-RC-2-tumor–bearing mice were randomized into three groups as saline, ^177^Lu-ZH2, and ^177^Lu-DPI-4452 and treated with a single dose of ^177^Lu-labeled radioligand (**Figure 4C–E, and Figure S6**). Compared with the saline group, both radioligand therapy groups showed significant tumor growth inhibition (both *P* < 0.001). In the saline group, most animals reached humane-endpoint criteria by approximately day 18–20 due to progressive tumor growth (**Figure 4C and Figure S6A**). Body-weight monitoring indicated acceptable tolerability. Although mild and transient weight loss was observed in both treated groups during the first 4 days, the body weights recovered thereafter and exceeded baseline by day 24 (relative body weight 106.43 ± 10.48% for ^177^Lu-ZH2 and 111.64 ± 6.31% for ^177^Lu-DPI-4452 in **Figure 4D** and **Figure S6B**). Survival analysis based on log-rank testing revealed significant differences among the groups (χ² = 19.69, *P* < 0.001; **Figure 4E**). Pairwise comparisons showed that both the ^177^Lu-ZH2 (*P* = 0.0001) and ^177^Lu-DPI-4452 (*P* = 0.0024) groups showed significantly prolonged survival compared with the saline group (median survival, 14 days), whereas survival did not differ significantly between the two radioligand-treated groups. Histopathological evaluation using hematoxylin-eosin staining was conducted to assess major organ toxicity across the three groups (**Figure 4F**). No evident pathological abnormalities were observed in major organs, including the heart, liver, lung, kidney, and stomach, supporting the tolerability of ^177^Lu-ZH2 at the administered dose.

### Clinical PET/CT studies of ^68^Ga-ZH2

**Figure 5:**
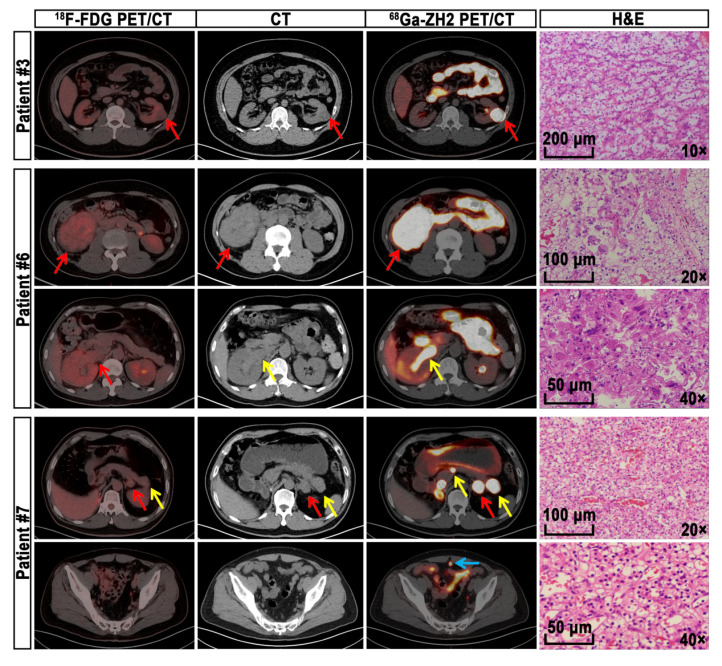
** Representative ^68^Ga-ZH2 and ^18^F-FDG PET/CT images from three patients with ccRCC.** Patient #3: A primary left renal tumor (red arrows) shows intense uptake on ^68^Ga-ZH2 PET/CT but false negative results on ^18^F-FDG PET/CT. The corresponding CT image revealed an exophytic, slightly hypodense nodule. Patient #6: A right renal primary tumor (red arrow) and a right renal-vein tumor thrombus (yellow arrows) show intense uptake on ^68^Ga-ZH2 PET/CT. In contrast, ^18^F-FDG PET/CT shows only mild uptake in these lesions, whereas CT demonstrates a marginally hyperdense soft-tissue mass. Patient #7 (follow-up after resection of right-sided ccRCC): Metastases in the left adrenal gland (red arrows), pancreatic head and tail (yellow arrows), and pelvic mesentery (blue arrow) were all clearly visualized on ^68^Ga-ZH2 PET/CT. ^18^F-FDG PET/CT detects the pancreatic-tail and left adrenal lesions with mild uptake but fails to identify the pancreatic-head and pelvic-mesenteric metastases, which are not clearly visualized on CT. ccRCC is confirmed through histopathological evaluation (hematoxylin–eosin staining) of surgical resection or biopsy specimens.

**Figure 6:**
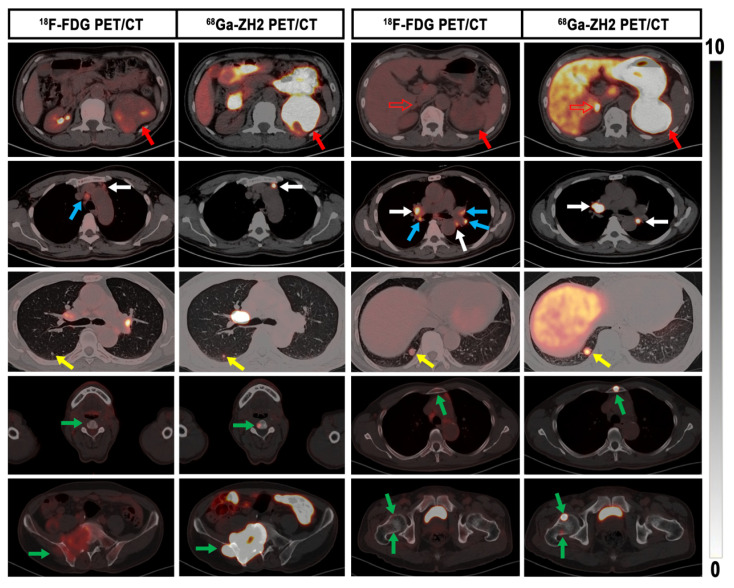
**^68^Ga-ZH2 PET/CT demonstrates superior performance in detecting both the primary tumor and metastatic lesions than ^18^F-FDG PET/CT in patient #4 with ccRCC.** The primary renal tumor (red arrows) and metastases in the right adrenal gland (red hollow arrows), lymph nodes (white arrows), lungs (yellow arrows), and multiple bones (green arrows) are clearly visualized on ^68^Ga-ZH2 PET/CT. In contrast, ^18^F-FDG PET/CT shows only mild uptake in a subset of these lesions (e.g., right lung, sternum, iliac bone, sacrum, and femoral head) and generates false-negative results for the metastases in the right adrenal gland, dorsal lung segment, third cervical vertebra, and right femoral trochanter. A mediastinal lymph node (blue arrow) shows increased ^18^F-FDG uptake, representing a false-positive finding.

**Figure 7:**
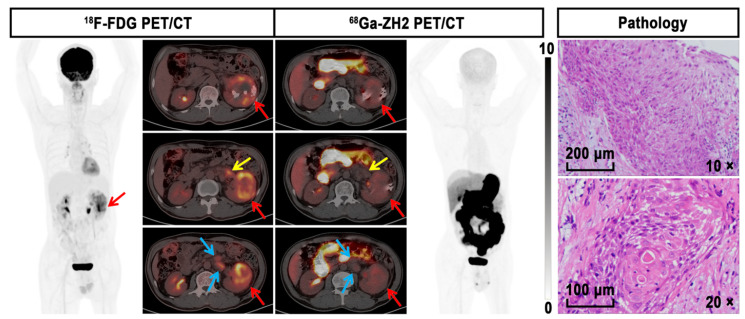
** Comparative ^18^F-FDG and ^68^Ga-ZH2 PET/CT images in patient #19 with left renal keratinizing squamous cell carcinoma.** The primary renal tumor (red arrows), left renal vein tumor thrombus (yellow arrows), and retroperitoneal lymph node metastasis (blue arrows) are all ^18^F-FDG-avid but demonstrate no uptake on ⁶⁸Ga-ZH2 PET/CT.

Before clinical studies, toxicity tests in C57BL/6 mice confirmed the safety of ^68^Ga-ZH2 (**Figure S7**). Subsequently, it was safely administered to all 21 patients via whole-body PET/CT. No immediate or delayed adverse reactions, clinically detectable pharmacological effects, or significant changes in vital signs were observed during or after tracer injection or throughout the PET/CT imaging procedure. The clinical characteristics of the study cohort are summarized in **Table 1**. Histopathological evaluation identified 19 patients with ccRCC (including two with postoperative recurrence or metastasis), one with keratinizing squamous cell carcinoma, and one with chromophobe renal cell carcinoma. CAIX immunohistochemistry was performed in 11 patients with ccRCC, representing more than half of the cohort. Most evaluated ccRCC specimens showed CAIX expression, with heterogeneous staining intensities ranging from weak to strong membranous and/or cytoplasmic staining (**Figure S8**). Of the 20 patients with ccRCC or squamous cell carcinoma, 19 (95%) completed paired ^18^F-FDG PET/CT within 1 week. This subgroup comprised 18 patients with ccRCC (13 men, 5 women; median age, 55.5 years; range, 35–73) and 1 patient with squamous cell carcinoma (a 58-year-old man). The remaining two patients (one with ccRCC, a 53-year-old man; one with chromophobe renal cell carcinoma, a 37-year-old woman) underwent ^68^Ga-ZH2 PET/CT only. Among non-tumor organs, the stomach (42.87 ± 21.64), small intestine (24.13 ± 8.40), and pancreas (13.79 ± 21.81) had the highest SUVmax uptake of ^68^Ga-ZH2. In contrast, other organs, including the liver (3.27 ± 1.06), kidneys (2.28 ± 0.82), large intestine (0.89 ± 0.70), aortic arch (0.94 ± 0.31), heart (0.68 ± 0.29), spleen (0.63 ± 0.28), bone (0.31 ± 0.32), lungs (0.30 ± 0.10), and muscle (0.26 ± 0.11) showed lower uptake (**Table S6**). On visual analysis, ^68^Ga-ZH2 PET/CT provided superior lesion delineation to ^18^F-FDG PET/CT, with a higher detection rate for primary tumors (16 vs. 5, *P* < 0.001) and metastases (73 vs. 27, *P* < 0.001) (**Table 2**). Additionally, 33 of the 89 CT-measurable lesions (37.1%) showed positive ^68^Ga-ZH2 uptake. In the representative cases, ^68^Ga-ZH2 PET/CT exhibited more intense tracer avidity and higher sensitivity than ^18^F-FDG PET/CT for ccRCC lesions, which was corroborated by histopathological analysis results (**Figure 5**). The uptake of ^68^Ga-ZH2 and ^18^F-FDG in all tumor lesions was further quantified (**Table 2**). In primary tumors, the SUVmax value of ^68^Ga-ZH2 was significantly higher than that of ^18^F-FDG (median, 108.25 vs. 3.00; z = -3.46, *P* < 0.001). Similarly, across all metastatic sites—including lymph nodes, lung, bone, and other metastases—the SUVmax of ^68^Ga-ZH2 was significantly higher than that of ^18^F-FDG (all *P <* 0.05). Moreover, ^68^Ga-ZH2 PET/CT achieved significantly higher tumor-to-background contrast than ^18^F-FDG PET/CT in both primary tumors and distant metastases, as shown by higher SUVmax (lesion) / SUVmax (mediastinum) values (SUV_T/M_, median, 10.13 vs. 1.28; *P* < 0.001) and SUVmax (lesion) / SUVmax (gluteus maximus) values (SUV_T/G,_ median, 26.5 vs. 2.13; *P* < 0.001). In a representative case of extensively metastatic ccRCC, ^68^Ga-ZH2 PET/CT demonstrated superior detection sensitivity by clearly visualizing all primary and metastatic lesions (**Figure S9**). In contrast, in another case, ^18^F-FDG PET/CT failed to detect several metastatic sites, showed only weak uptake in others, and yielded false-positive findings in lymph nodes (**Figure 6 and Figure S10**). Among the enrolled patients, two were diagnosed with non-ccRCC histology. Patient #19, who had keratinizing squamous cell carcinoma, underwent paired ⁶⁸Ga-ZH2 and ^18^F-FDG imaging (**Figure 7 and Figure S11**). On ^68^Ga-ZH2 PET/CT, neither the primary tumor nor the nodal metastases showed any discernible tracer uptake, whereas ^18^F-FDG PET/CT demonstrated markedly high tracer uptake. Patient #21, who was diagnosed with chromophobe renal cell carcinoma, underwent only ^68^Ga-ZH2 PET/CT. This test showed no tracer uptake in the primary lesion (**Figure S12**).

## Discussion

Because of its high and specific tumor expression, CAIX has long been recognized as an attractive theranostic target in ccRCC. Despite extensive efforts, CAIX-directed radiotheranostics have not achieved broad clinical translation. Antibody-based strategies, such as radiolabeled girentuximab, demonstrated tumor targeting properties, which were impeded by prolonged circulation times and unfavorable dosimetry profiles 24, 25. In contrast, most small-molecule CAIX inhibitors exhibited insufficient tumor retention or suboptimal tumor-to-kidney ratios, which is a major limitation in renal malignancies 17-21. The cyclic peptide DPI-4452 represented a notable advance by improving pharmacokinetics; however, persistent gastrointestinal uptake remained a major obstacle to clinical application 22, 23. Previously, a tri-cysteic acid linker was introduced to alter the CAIX-targeting cyclic peptide. This resulted in the theranostic pair ^18^F/^177^Lu-C3-DPI, which enhanced tumor uptake and tumor-to-background ratios while showing high gastrointestinal uptake 26. In the present study, this limitation was not solely attributed to ligand affinity; rather, it reflects a fundamental challenge in decoupling tumor targeting from physiological gastrointestinal CAIX binding. A refined CAIX-targeted theranostic strategy was proposed through systematic, structure-guided ligand redesign and validation across preclinical models and first-in-human studies.

Structural optimization of the cyclic peptide scaffold demonstrated that relatively subtle architectural modifications can exert profound effects on organ-level biodistribution 27. The ZH1–ZH5 design offered a targeted structure–activity relationship (SAR) framework to elucidate the impact of structural elements of the cyclic peptide scaffold on CAIX binding and organ-level biodistribution. In ZH1–ZH3, DOTA was replaced with DOTAGA to increase molecular polarity and hydrophilicity, whereas variations in the linker length and composition were used to modulate pharmacokinetics, including tumor retention, renal clearance, and gastrointestinal uptake. Additionally, altering the conjugation site of the chelator–linker moiety in ZH4 and ZH5 markedly reduced CAIX affinity; therefore, the spatial presentation of the CAIX-binding motif is critical for receptor recognition. ZH2 attained the most favorable balance, lowering gastrointestinal retention in preclinical studies while maintaining strong tumor uptake and sub-nanomolar CAIX affinity. These findings provide credence to the idea that rational tuning of the chelator–linker architecture can partially separate tumor targeting and physiological gastrointestinal CAIX binding 28.

Radiolabeled ZH2 displayed favorable cellular behavior, including enhanced uptake, prolonged intratumoral retention, and reduced efflux, supporting its potential for CAIX-targeted imaging and radioligand therapy. *In vivo*, ^68^Ga-ZH2 achieved an optimal balance between high tumor uptake and reduced accumulation in non-tumor organs, particularly in the stomach, distinguishing it from earlier CAIX-targeting agents 17-19. Importantly, this optimization was evident in the therapeutic setting, where ¹⁷⁷Lu-ZH2 showed high tumor-to-healthy tissue ratios in most organs and comparable tumor exposure but lower stomach exposure than ¹⁷⁷Lu-DPI-4452. Radioligand therapy with ¹⁷⁷Lu-ZH2 significantly inhibited tumor development and prolonged survival in tumor-bearing mice, supporting its potential as a promising CAIX-targeted theranostic candidate. The relatively higher renal exposure of ¹⁷⁷Lu-ZH2 should be carefully evaluated in future therapeutic translation, although its tumor-to-kidney AUC ratio was comparable to that reported for clinically approved radioligand therapies, such as ¹⁷⁷Lu-PSMA-617 7, 29, 30. No noticeable renal or gastrointestinal histopathological abnormalities were observed under the current experimental conditions; however, comprehensive safety evaluation, long-term toxicity assessment, and absorbed dose estimation for tumors and critical organs, particularly the kidneys, are essential to inform dose optimization and further define the ¹⁷⁷Lu-ZH2 therapeutic window.

The exact mechanism underlying this tumor–gastrointestinal dissociation is still unclear, although ZH2 showed markedly reduced gastric retention than DPI-4452. Considering ZH2 maintained strong tumor uptake and sub-nanomolar CAIX binding, the current findings suggest that this effect is unlikely to result from a simple decrease in intrinsic CAIX affinity. Instead, ligand physicochemical properties and potential differences in carbonic anhydrase isoform interactions may affect reduced gastrointestinal uptake, which is similar with earlier findings for modified DPI-4452 derivatives 28. Particularly, carbonic anhydrase II (CAII) is abundantly expressed in the gastric mucosa and may contribute to the nonspecific gastrointestinal retention of CAIX-targeted ligands 31. Therefore, the modified chelator–linker architecture and increased hydrophilicity of ZH2 may reduce effective gastric residence and/or off-target interaction with gastrointestinal carbonic anhydrase isoforms while retaining tumor-associated CAIX binding.

The translational relevance of this strategy was validated in a prospective first-in-human PET/CT study. In that cohort, CAIX immunohistochemistry was performed on tumour specimens from 11 patients with ccRCC and confirmed CAIX expression in the evaluated lesions. The observation that strong membranous CAIX staining corresponded to high tumour uptake on ^68^Ga-ZH2 PET/CT provides histopathological support for the biological specificity of tracer accumulation. ^68^Ga-ZH2 demonstrated markedly superior sensitivity and tumor-to-background contrast relative to standard ^18^F-FDG PET/CT, enabling reliable detection of primary tumors, metastatic lesions, and venous tumor thrombi that were occult or equivocal on ^18^F-FDG imaging. Notably, ^68^Ga-ZH2 showed broader diagnostic utility than the previously reported CAIX-targeted tracers 32, which have demonstrated limited sensitivity for nodal or distant metastases. Thus, CAIX-targeted PET with ^68^Ga-ZH2 substantially improves staging accuracy and lesion detection in ccRCC, with direct implications for patient stratification and therapeutic decision-making.

Several limitations of this study should be acknowledged. First, the clinical cohort had a modest size and was primarily designed to evaluate diagnostic performance rather than therapeutic outcomes. Second, there was no delayed imaging; all clinical PET/CT images were acquired at a single time point, 60 min after injection. Future studies incorporating multi-time-point imaging are warranted to optimize the clinical acquisition protocol and to comprehensively characterize the pharmacokinetics of ⁶⁸Ga-ZH2. Additionally, despite no noticeable adverse effects after ⁶⁸Ga-ZH2 administration in preclinical and clinical diagnostic settings, these findings do not confirm the therapeutic safety of ¹⁷⁷Lu-ZH2. Toxicity evaluation for therapy requires a thorough assessment of ¹⁷⁷Lu-ZH2, including absorbed-dose estimation, organ toxicity assessment, and longer-term follow-up. Finally, ^68^Ga/^177^Lu-ZH2 showed reduced gastrointestinal retention than ^68^Ga/^177^Lu-DPI-4452 in preclinical models. Nonetheless, a head-to-head comparison of ^68^Ga-ZH2 with ^68^Ga-DPI-4452 or other CAIX-targeted peptides has not been conducted in patients with ccRCC. Nevertheless, the strong concordance between preclinical and clinical findings underscores the robustness of the design strategy and provides a clear framework for subsequent therapeutic trials.

## Conclusion

In preclinical evaluation, ^68^Ga/^177^Lu-ZH2 demonstrates strong CAIX targeting with favorable pharmacokinetics, achieving effective tumor uptake and minimizing gastrointestinal retention. ^177^Lu-ZH2 inhibits tumor development and prolongs survival without evident toxicity. In preliminary clinical PET/CT imaging, ^68^Ga-ZH2 PET/CT is safe and shows superior performance in detecting primary tumors and metastases than ^18^F-FDG PET/CT, with a higher tumor-to-background contrast. Overall, this study demonstrates that rational, structure-guided ligand redesign can mitigate a critical biological barrier that has previously limited the clinical translation of CAIX-targeted radiotheranostics. These results allow for sensitive ccRCC detection, lay the groundwork for patient selection and targeted radioligand therapy, and support additional assessment of CAIX-directed precision oncology in clinical studies by characterizing the ^68^Ga/^177^Lu-ZH2 pair as a promising imaging and therapeutic candidate.

## Supplementary Material

Supplementary figures and tables.

## Figures and Tables

**Table 1:** Characteristics of the patients who completed a paired PET/CT scan.

Patient no.	Sex	Age (yr)	Condition	Pathology	Tumor location	Lesions	^18^F-FDG-SUVmax	^68^Ga-ZH2-SUVmax
1	M	68	AO	ccRCC	Lung mets	2/0	1.0	11.1
2	F	50	BO	ccRCC	Primary tumor	1/0	15.8	14.1
					Lymph node, lung, and bone mets	5/1	5.4 (3.6, 12.5)	3.6 (2.1, 5.0)
3	M	37	BO	ccRCC	Primary tumor	1/0	2.4	244
4	M	57	BO	ccRCC	Primary tumor	1/0	6.1	186.6
					Lymph node, lung, bone, and right adrenal gland mets	4/18	3.4 (2.5,5.5)	24.5 (7.4, 35.2)
5	M	70	BO	ccRCC	Primary tumor	1/0	3.8	81.1
6	M	56	BO	ccRCC	Primary tumor	1/0	6.7	47.8
					Left renal vein	1/0	3.9	35.1
7	M	63	AO	ccRCC	Left adrenal gland, pancreas, and pelvic mesentery mets	3/4	2.1 (1.7, 2.4)	145.8 (79.7, 222.7)
8	F	68	BO	ccRCC	Primary tumor	1/0	15.5	65.1
					Left renal vein, lymph node, and lung mets	0/18	1.2 (0.9, 2.9)	3.7 (2.0, 8.5)
9	M	45	BO	ccRCC	Primary tumor	1/0	2.6	169.2
10	M	71	BO	ccRCC	Primary tumor	1/0	4	84.3
					Right renal vein and lung mets	3/14	1.0 (0.7,1.4)	2.9 (1.3,4.0)
11	M	55	BO	ccRCC	Primary tumor	1/0	2.4	178.1
12	F	54	BO	ccRCC	Primary tumor	1/0	2.6	102.3
13	M	54	BO	ccRCC	Primary tumor	1/0	7.4	110.6
14	F	54	BO	ccRCC	Primary tumor	1/0	3.2	96.7
15	M	35	BO	ccRCC	Primary tumor	1/0	2.8	223.2
16	M	50	BO	ccRCC	Primary tumor	1/0	2.5	123.5
17	F	69	BO	ccRCC	Primary tumor	0/1	2.6	105.9
18	M	73	BO	ccRCC	Primary tumor	1/0	2.3	195.9

SUVmax, maximum standardized uptake value; M, male; F, female; BO, before operation; AO, after operation; ccRCC, clear cell renal cell carcinoma; mets, metastases. Lesions: Measurable/nonmeasurable lesions on CT according to RESIST 1.1 criteria. SUVmax reporting: single values indicate individual measurements; two values are reported as the mean; ≥ 3 values are reported as the median (first quartile, third quartile).

**Table 2:** Comparison of diagnostic efficacy between ^68^Ga-ZH2 and ^18^F-FDG in patients with ccRCC.

Parameter		Primary tumor		Lymph-node mets	Lung		Bone		Other mets		Total		
SUVmax	68Ga-ZH2	108.25 (83.50, 180.23)	31.40 (15.20, 46.50)			2.80 (1.48, 4.83)		24.20 (6.00, 32.25)		108.80 (29.00, 176.45)		9.40 (3.00, 47.80)	
	18F-FDG	3.00 (2.58, 6.25)	11.40 (5.70, 14.40)			1.10 (0.73, 1.48)		2.70 (2.30, 4.30)		2.30 (1.75, 2.80)		2.40 (1.20, 4.00)	
	*P*	< 0.001	0.028			< 0.001		0.001		0.003		< 0.001	
SUV_T/M_	68Ga-ZH2	199.62 (110.15, 251.25)	62.80 (29.60, 66.67)			3.37 (2.18, 6.95)		2.99 (0.94, 16.38)		116.00 (26.36, 160.41)		10.13 (2.60, 74.80)	
	18F-FDG	1.80 (1.36, 3.23)	5.76 (3.56, 7.13)			0.59 (0.42, 0.80)		1.69 (1.44, 2.67)		1.00 (0.84, 1.47)		1.28 (0.63, 2.35)	
	*P*	< 0.001	0.011			< 0.001		0.118		0.003		< 0.001	
SUV_T/G_	68Ga-ZH2	1064.50 (771.00, 1459.07)	291.00 (148.00, 314.00)			12.75 (6.75, 25.38)		1.36 (0.78, 9.22)		290.00 (83.50, 529.67)		26.50 (7.40, 300.00)	
	18F-FDG	6.45 (4.92, 9.72)	16.29 (8.14, 20.57)			1.50 (1.04, 2.09)		1.43 (0.77, 2.61)		2.13 (1.77, 3.25)		2.13 (1.29, 5.90)	
	*P*	< 0.001	0.011			< 0.001		0.047		0.003		< 0.001	
Lesions (*n*)	68Ga-ZH2	16	9			38		15		11		89	
	18F-FDG	5	8			8		6		5		40	
	*P*	< 0.001	1			< 0.001		0.004		0.031		< 0.001	
													

SUVmax, maximum standardized uptake value; SUV_T/M_, SUVmax (lesion) / SUVmax (mediastinum); SUV_T/G_, SUVmax (lesion) / SUVmax (gluteus maximus); mets, metastasis; other mets include venous tumor thrombus, adrenal gland metastasis, pancreatic metastasis, and pelvic mesenteric metastasis. Lesions indicate the number of lesions with positive uptake on ^68^Ga-ZH2 or ^18^F-FDG PET/CT. Data are presented as median (first quartile, third quartile). The *P*-values are based on paired statistical comparison. *n* = 18.

## Data Availability

All data generated or analysed during this study are included in this published article and its supplementary material files.

## Abbreviations

CAIX: carbonic anhydrase IX
ccRCC: clear cell renal cell carcinoma
PET/CT: positron emission tomography/computed tomography
PSMA: prostate-specific membrane antigen
HPLC: high-performance liquid chromatography
PBS: phosphate-buffered saline
%ID/1 mio cells: the percentage of the injected dose per million cells
%ID/g: percentage injected dose per gram
ROI: region of interest
SUVmax: maximum standardized uptake value
SUV_T/M_: SUVmax (lesion) / SUVmax (mediastinum)
SUV_T/G_: SUVmax (lesion) / SUVmax (gluteus maximus)
SD: standard deviation
RCPs: radiochemical purities
p.i.: post-injection
AUC: under the curve
SAR: structure–activity relationship
CAII: carbonic anhydrase II
