# Supplementary material for: Structure-based redesign enables clinical translation of CAIX-targeted theranostics in clear cell renal cell carcinoma
Source: Theranostics. 2026 Jul 20;16(14):8247–62. doi: 10.7150/thno.134970 (PMC13440624; doi:10.7150/thno.134970)
Supplement: Supplementary file 1 [file thnov16p8247s1.pdf]

Supplementary Material

Structure-based redesign enables clinical translation of CAIX-targeted theranostics in clear cell renal cell carcinoma

Zexin Xu[1,2#], Hui Zhou[3#], Ye Dong[1#], Dan Feng[1], Baocheng Chen[1], Hongxin Li[1,2], Ruitao Yang[1,2], Ningjie Li[1,2], Yuting Cao[4], Hankuan He[4], Han Wu[4], Yuhua Zhong[4], Hubing Wu[1], Liang Zhao[3✉], Haojun Chen[3✉], Kongzhen Hu[1,2✉]

[1]Department of Nuclear Medicine, Nanfang Hospital, Southern Medical University, 1838 Guangzhou North Road, Guangzhou, Guangdong Province, 510515, China

[2] School of Pharmaceutical Sciences, Southern Medical University, 1838 Guangzhou North Road, Guangzhou, 510515, China.

[3] Department of Nuclear Medicine and Minnan PET Center, The First Affiliated Hospital of Xiamen University, School of Medicine, Xiamen University, Xiamen, 361003, China.

[4]Department of Rehabilitation Medicine, Nanfang Hospital, Southern Medical University, 1838 Guangzhou North Road, Guangzhou, Guangdong Province, 510515, China

# These authors contributed equally to this work.

✉Corresponding authors: Liang Zhao, E-mail: wzhaoliang01@163.com; Haojun Chen, E-mail: leochen0821@foxmail.com; Kongzhen Hu, E-mail: stonglass@163.com.

Table of contents

1. Chemistry ······ **3**

1.1. General information ······ **3**

1.2. Schemes ······ **5**

Scheme 1. Solid-phase synthesis route of ZH1. ······ 5

Scheme 2. Solid-phase synthesis route of ZH2. ······ 6

Scheme 3. Solid-phase synthesis route of ZH3. ······ 7

Scheme 4. Solid-phase synthesis route of ZH4. ······ 8

Scheme 5. Solid-phase synthesis route of ZH5. ······ 9

1.3. Synthesis procedure ······ **10**

1.4. Spectroscopic and chromatogram data ······ **27**

2. Supplementary Figures ······ **38**

Figure S1. The quality control of the radiolabeled ligands.. ······ 38

Figure S2. In vitro stability of ^{68}Ga-ZH1–5 and ^{68}Ga-DPI-4452 in PBS, mouse serum and human serum for 2 h. ······ 39

Figure S3. In vitro stability of ^{177}Lu-ZH2 and ^{177}Lu-DPI-4452 in PBS, mouse serum and human serum for 24 h. ······ 40

Figure S4. Time-activity curves of ^{68}Ga-ZH1–5 and ^{68}Ga-DPI-4452 in selected organs and tumor for 2 h. ······ 41

Figure S5. The original gel blots of WB analysis, *n* = 3. ······ 42

Figure S6. Therapeutic efficacy of CAIX-targeted ^{177}Lu-radiolabeling ligands. ······ 43

Figure S7. Toxicity test of ^{68}Ga-ZH2. ········ 44

Figure S8. Immunohistochemical staining of patient tissue samples for CAIX. ········ 46

Figure S9. ^{68}Ga-ZH2 PET/CT in Patient #20 with Metastatic ccRCC. ········ 46

Figure S10. Imaging and histopathological of a metastatic lesion in the first sacral (S1) vertebra (Patient #4). ········ 47

Figure S11. Histopathology of the resected left renal mass (Patient #19). ········ 48

Figure S12. ^{68}Ga-ZH2 PET/CT in Patient #21 with chromophobe renal cell carcinoma. ········ 49

3. Supplementary Tables ········ 49

Table S1. Ex vivo biological distribution of ^{68}Ga-ZH1–3 and ^{68}Ga-DPI-4452 at 1 h. ·· 49

Table S2. The values of tumor to organ or tissue of ^{68}Ga-ZH2 and ^{68}Ga-DPI-4452 based on the ex vivo biodistribution data. ········ 50

Table S3. Ex vivo biological distribution of ^{177}Lu-ZH2 at 1, 4, 24, 72 and 168 h. ······ 51

Table S4. Ex vivo biological distribution of ^{177}Lu-DPI-4452 at 1, 4, 24, 72 and 168 h. 52

Table S5. The values of AUC and AUC ratio of tumor to organ or tissue of ^{177}Lu-ZH2 and ^{177}Lu-DPI-4452 based on the ex vivo biodistribution data. ········ 53

Table S6. The non-tumor organs and primary tumor uptakes (SUVmax) of ^{68}Ga-ZH2 in the patients with renal clear cell carcinoma. ········ 55

1. Chemistry

1.1. General information

Liquid chromatography–mass spectrometry (LC–MS) was performed on AB SCIEX

3200 for quality control. Column: Sepax C18 (250 mm × 4.6 mm, 5 μm; Sepax Technologies, USA). High-performance liquid chromatography (HPLC) was equipped with DAD-UV detector (Shimadzu, Japan) and B-FC-3200 high energy PMT detector (Bioscan. Inc, Washington DC, USA) conducting in a LC-20AD HPLC system (Shimadzu, Japan). Column: XDB-C18 analytic columns (4.6 × 150 mm, 5 μm; Agilent Technologies, USA).

Mobile phase I for chemical quality control: 0.1% trifluoroacetic acid (TFA) in H_2O (solvent A) and CH_3CN (solvent B). Flow rate of 1.0 mL/min. The linear gradient was 25%–45% (solvent B) from 0 to 20 min. The elution process was detected with an ultraviolet detector at 220 nm.

Mobile phase II for radiochemical quality control and purification: 0.1% TFA in H_2O (solvent A) and 0.1% TFA in CH_3CN (solvent B). Flow rate of 1.0 mL/min. Method 1 was used for radio-identification and radiochemical purity of ^{68}Ga--labeled ligands: 10%–10% (solvent B) from 0 to 2 min; 10%–80% (solvent B) from 2 to 10 min; 80%–80% (solvent B) from 10 to 14 min; 80%–10% (solvent B) from 14 to 15 min. Method 2 was used for ^{177}Lu-labeled ligands purification: 20%–20% (solvent B) from 0 to 2 min; 20%–35% (solvent B) from 2 to 119 min; 35%–20% (solvent B) from 119 to 128 min.

1.2. Schemes

Scheme 1. Solid-phase synthesis route of ZH1.

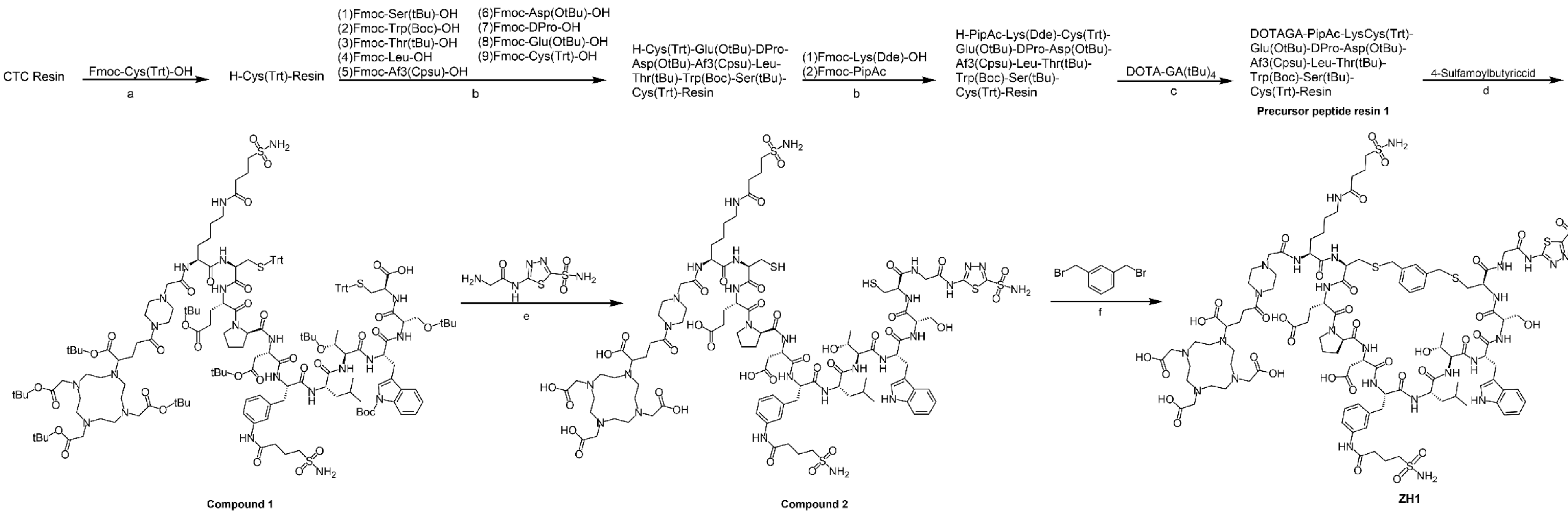

Reagents and conditions. (a) (i) DIPEA, in DCM, rt, 4 h; (ii) 20% piperidine in DMF. (b) DIC (3 equivalents), HOBt (3 equivalents), in DMF, rt, 2 h. (c) (i) HATU (1 equivalents), DIPEA (3 equivalents), in DMF, rt, 2 h; (ii) 4% $N_2H_4 \cdot H_2O$ in DMF. (d) (i) HATU (1 equivalents), DIPEA (3 equivalents), in DMF, rt, 2 h; (ii) 1% TFA in DCM, rt, 2 h. (e) (i) EDCI:HOOBT:NMM (1:1:1) in DCM, rt, 12 h; (ii) 95% TFA in H_2O, rt, 2 h. (f) NH_4HCO_3:MeCN (1:1), rt, 2 h.

Scheme 2. Solid-phase synthesis route of ZH2.

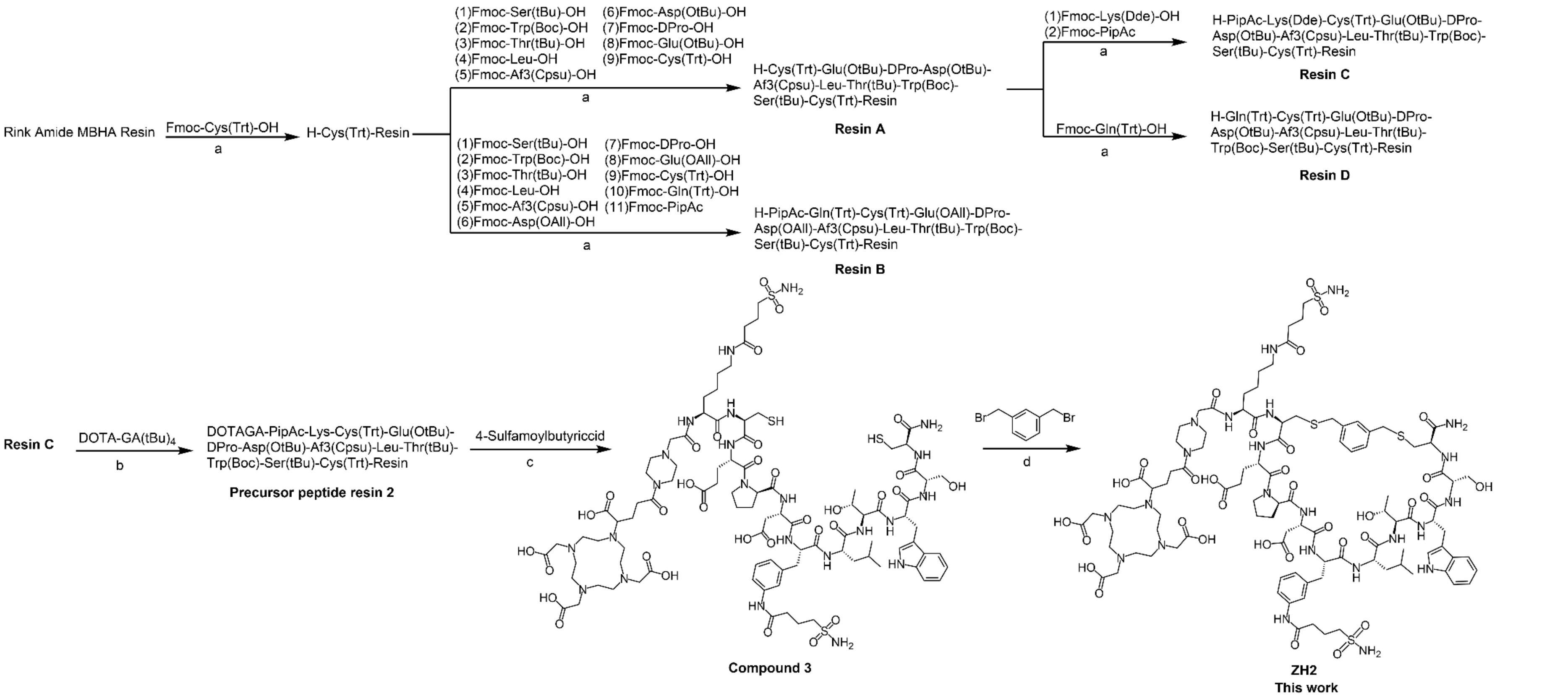

Reagents and conditions. (a) (i) DIC (3 equivalents), HOBt (3 equivalents), in DMF, rt, 2 h. (ii) 20% piperidine in DMF. (b) (i) HATU (1 equivalents), DIEA (3 equivalents), in DMF, rt, 2 h; (ii) 4% $N_2H_4{\bullet}H_2O$ in DMF. (c) (i) HATU (1 equivalents), DIPEA (3 equivalents), in DMF, rt, 2 h; (ii) 95% TFA in H_2O, rt, 2 h. (d) NH_4HCO_3:MeCN (1:1), rt, 2 h.

Scheme 3. Solid-phase synthesis route of ZH3.

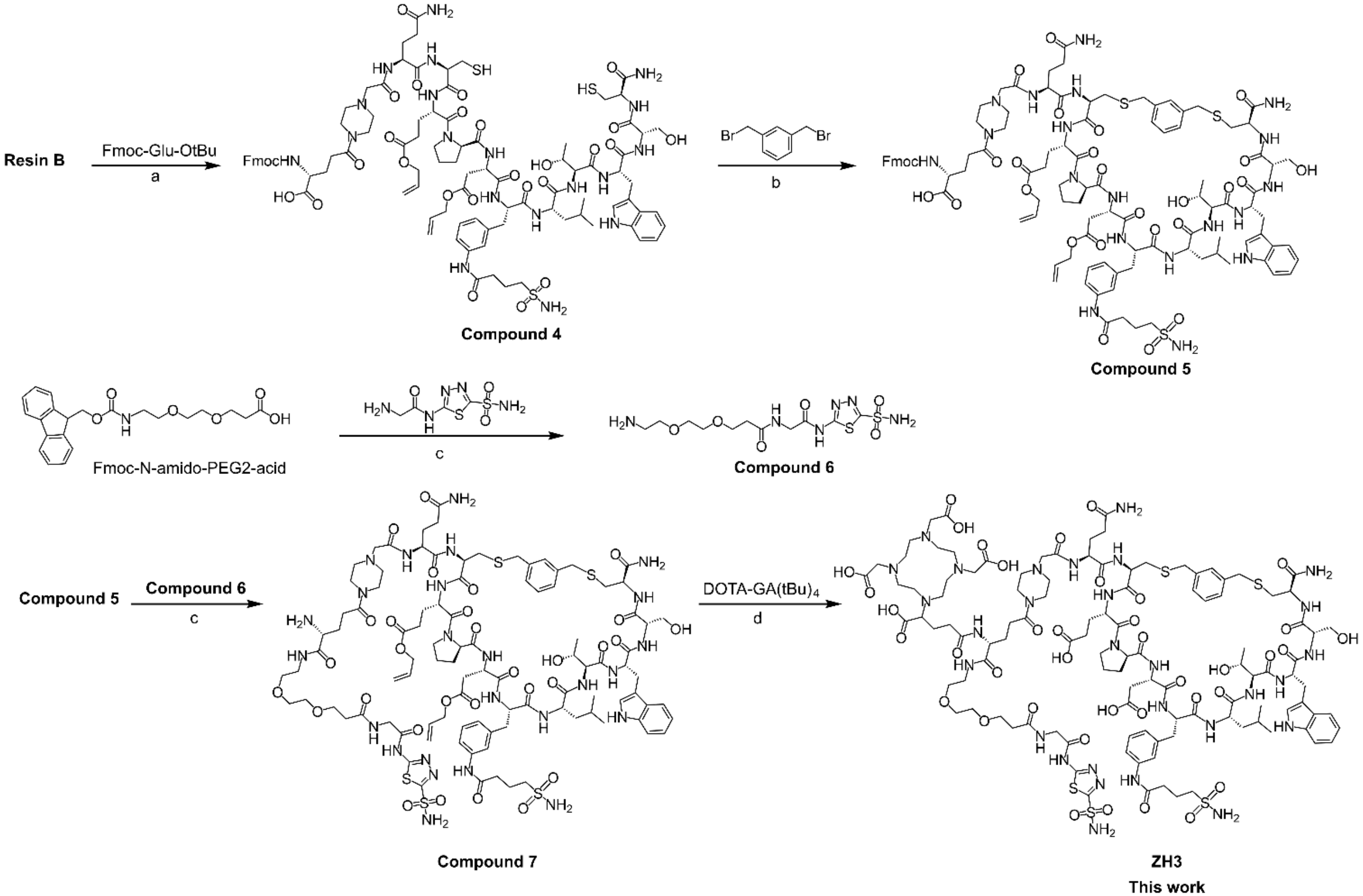

Reagents and conditions. (a) (i) DIC (3 equivalents), HOBt (3 equivalents), in DMF, rt, 2 h. (ii) 95% TFA in H_2O, rt, 2 h. (b) NH_4HCO_3:MeCN (1:1), rt, 2 h. (c) (i) EDCI:HOOBT:NMM (1:1:1) in DCM, rt, 12 h; (ii) 20% piperidine in DMF. (d) (i) HATU (1 equivalents), DIEA (3 equivalents), in DMF, rt, 2 h; (ii) $Pd(PPh_3)_4$, N-Methylaniline in DMF, rt, 12 h; (iii) 95% TFA in H_2O, rt, 2 h.

Scheme 4. Solid-phase synthesis route of ZH4.

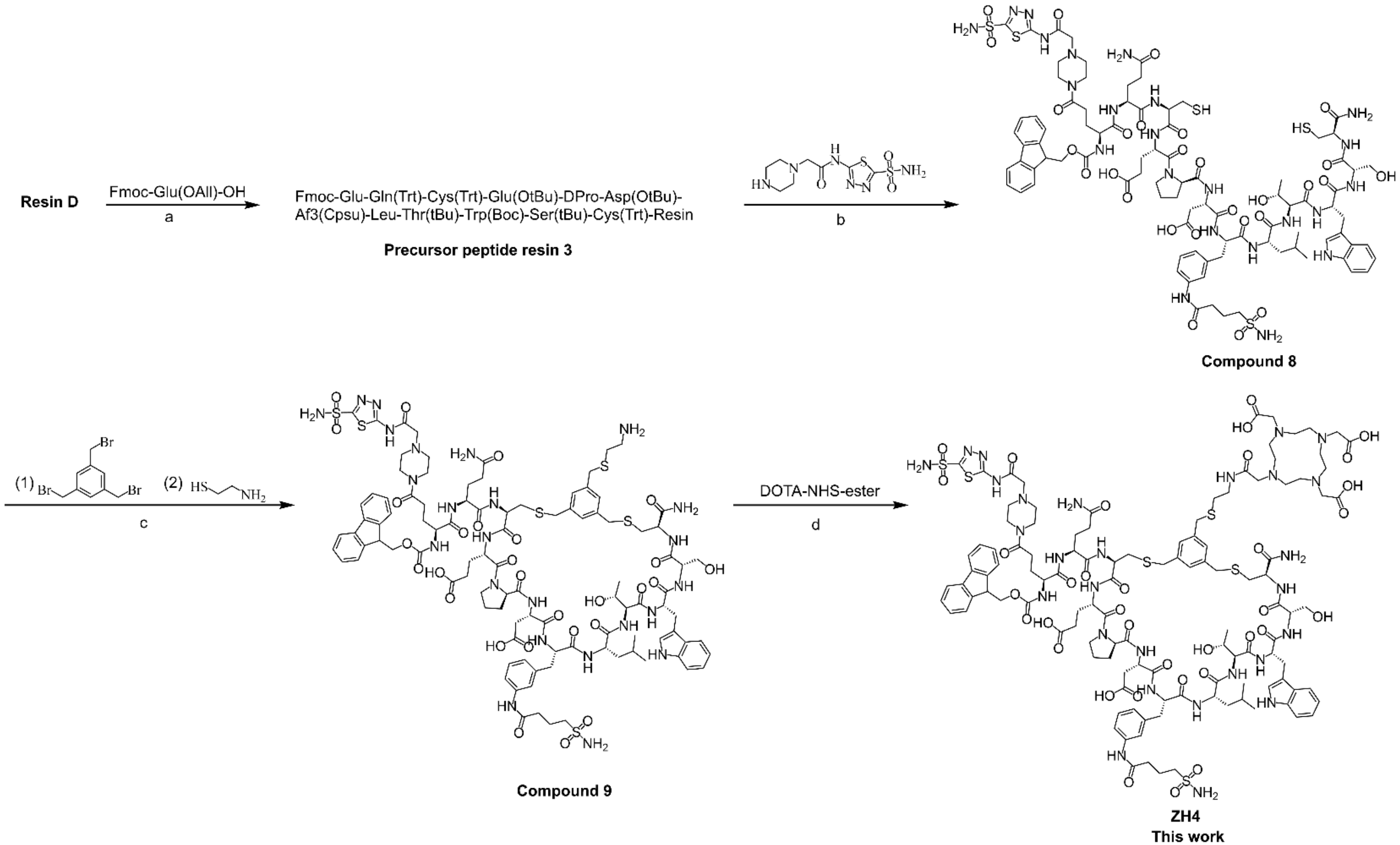

Reagents and conditions. (a) (i) DIC (3 equivalents), HOBt (3 equivalents), in DMF, rt, 2 h. (ii) $Pd(PPh_3)_4$, $CHCl_3$, AcOH, N-Methylaniline in DMF, rt, 12h. (b) (i) HATU (1 equivalents), DIEA (3 equivalents), in DMF, rt, 2 h; (ii) 95% TFA in H_2O, rt, 2 h. (c) NH_4HCO_3:MeCN (1:1), rt, 2 h. (d) (i) HATU (1 equivalents), DIEA (3 equivalents), in DMF, rt, 2 h; (ii) 20% piperidine in DMF, rt, 2 h.

Scheme 5. Solid-phase synthesis route of ZH5.

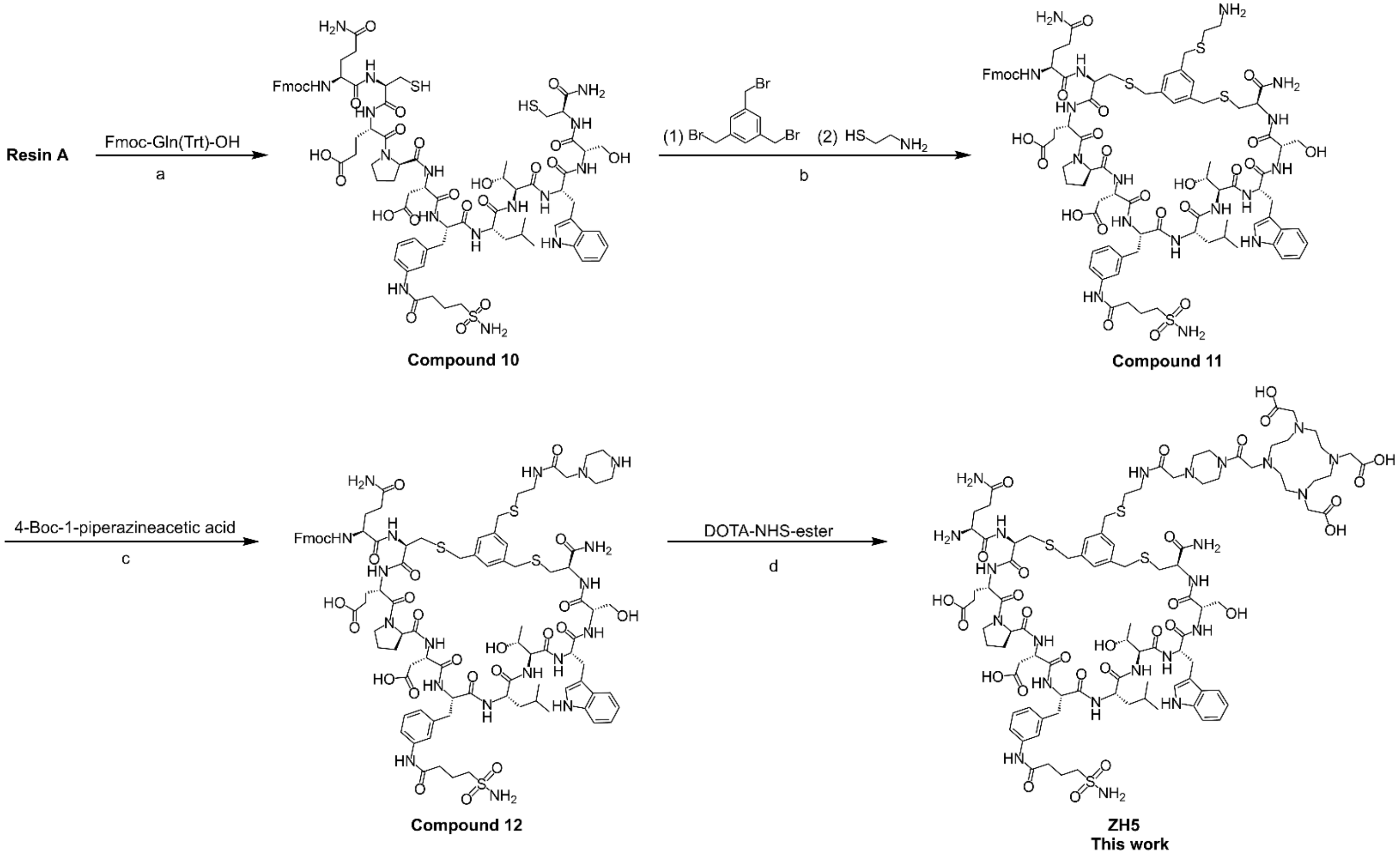

Reagents and conditions. (a) (i) DIC (3 equivalents), HOBt (3 equivalents), in DMF, rt, 2 h. (ii) 95% TFA in H_2O in H_2O, rt, 2 h. (b) NH_4HCO_3:MeCN (1:1), rt, 2 h. (c) (i) DCC:HOSu:DIEA (1:1:1.2) in DMF, rt, 3 h; (ii) 95% TFA in H_2O, rt, 2 h. (d) (i) HATU (1 equivalents), DIEA (3 equivalents), in DMF, rt, 2 h; (ii) 20% piperidine in DMF, rt, 2 h.

1.3. Synthesis procedure

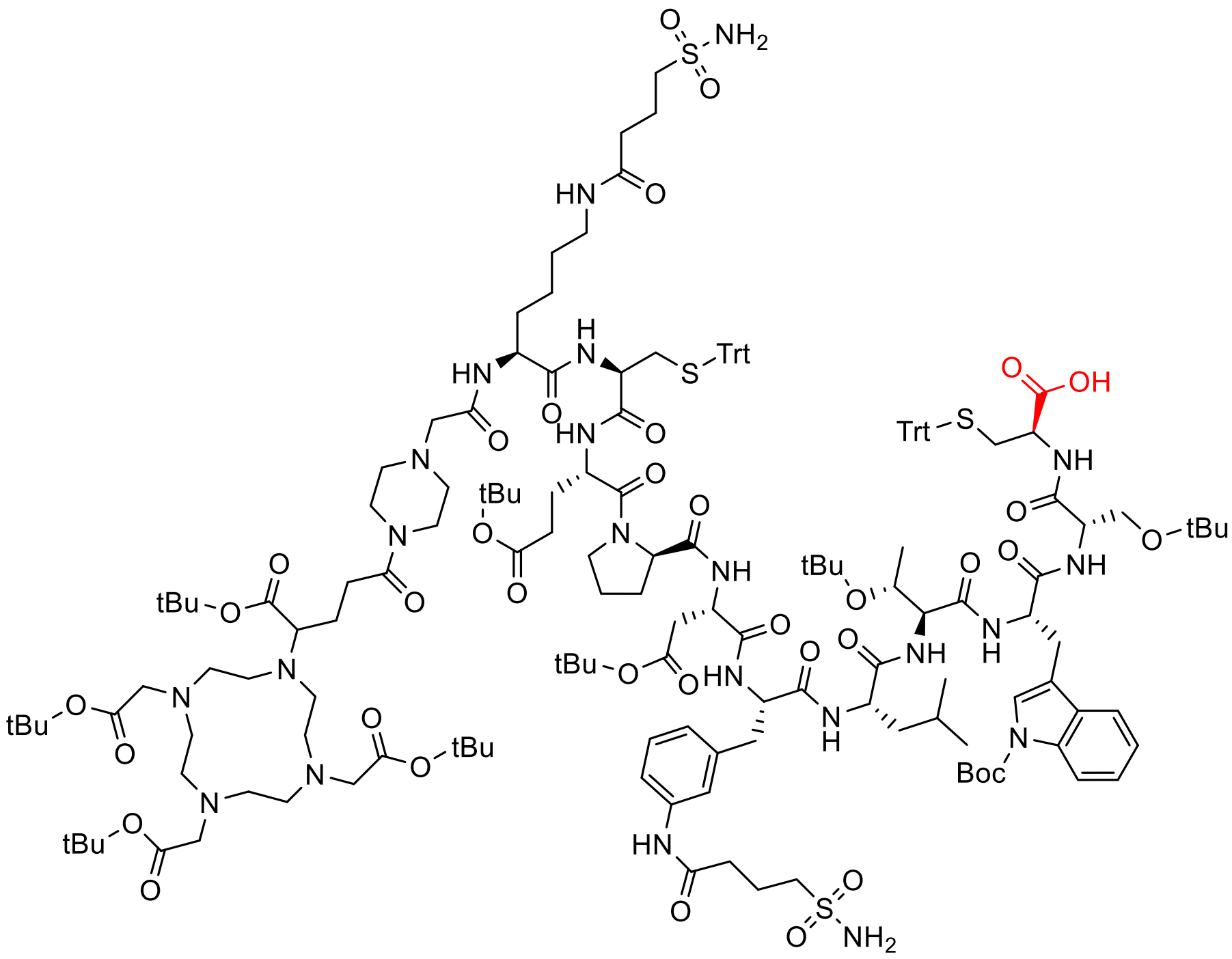

Compound 1

N-(N-(Na-(N-(((2S)-2-((2S)-4-(tert-butoxy)-2-((2R)-1-((2S)-5-(tert-butoxy)-2-((2R)-2-((2S)-2-(2-(4-(5-(tert-butoxy)-5-oxo-4-(4,7,10-tris(2-(tert-butoxy)-2-oxoethyl)-1,4,7,10-tetraazacyclododecan-1-yl)pentanoyl)piperazin-1-yl)acetamido)-6-(4-sulfamoylbutanamido)hexanamido)-3-(tritylthio)propanamido)-5-oxopentanoyl)pyrrolidine-2-carboxamido)-4-oxobutanamido)-3-(3-(4-sulfamoylbutanamido)phenyl)propanoyl)-L-leucyl)-O-(tert-butyl)-L-threonyl)-1-(tert-butoxycarbonyl)-L-tryptophyl)-O-(tert-butyl)-L-seryl)-S-trityl-L-cysteine (Compound 1)

To a solution of **precursor peptide resin 1** (31.2 mg, containing 0.01 mmol of peptide) and 4-sulfamoylbutyric acid (5.0 mg, 0.03 mmol, 3.0 equiv) in anhydrous DMF (1.5 mL) were added HATU (3.8 mg, 0.01 mmol, 1.0 equiv) and DIPEA (5.2 µL, 0.03 mmol, 3.0 equiv). The mixture was stirred at room temperature for 2 h. The resin was then washed with DMF (3 × 2 mL) and DCM (3 × 2 mL). To cleave the peptide from the CTC resin, the dried resin was treated with 1% TFA in DCM (2 mL) at room temperature for 2 h. This step selectively releases the peptide from the solid support while retaining acid-labile side chain protecting groups. The cleavage solution was collected by filtration, and the resin was washed with DCM (2 × 1 mL). The combined filtrates were concentrated under a gentle stream of nitrogen to afford the crude peptide with a free carboxyl group at the C-terminus, indicating complete removal of the peptide from the CTC resin. This crude peptide was then purified using HPLC on a C18 column obtaining **compound 1** (15.7 mg, yield approximately 14.5%). MS (m/z): calced for $C_{167}H_{240}N_{22}O_{36}S_4$ $[M + 2H]^{2+}$, 1629.8, found, 1630.95.

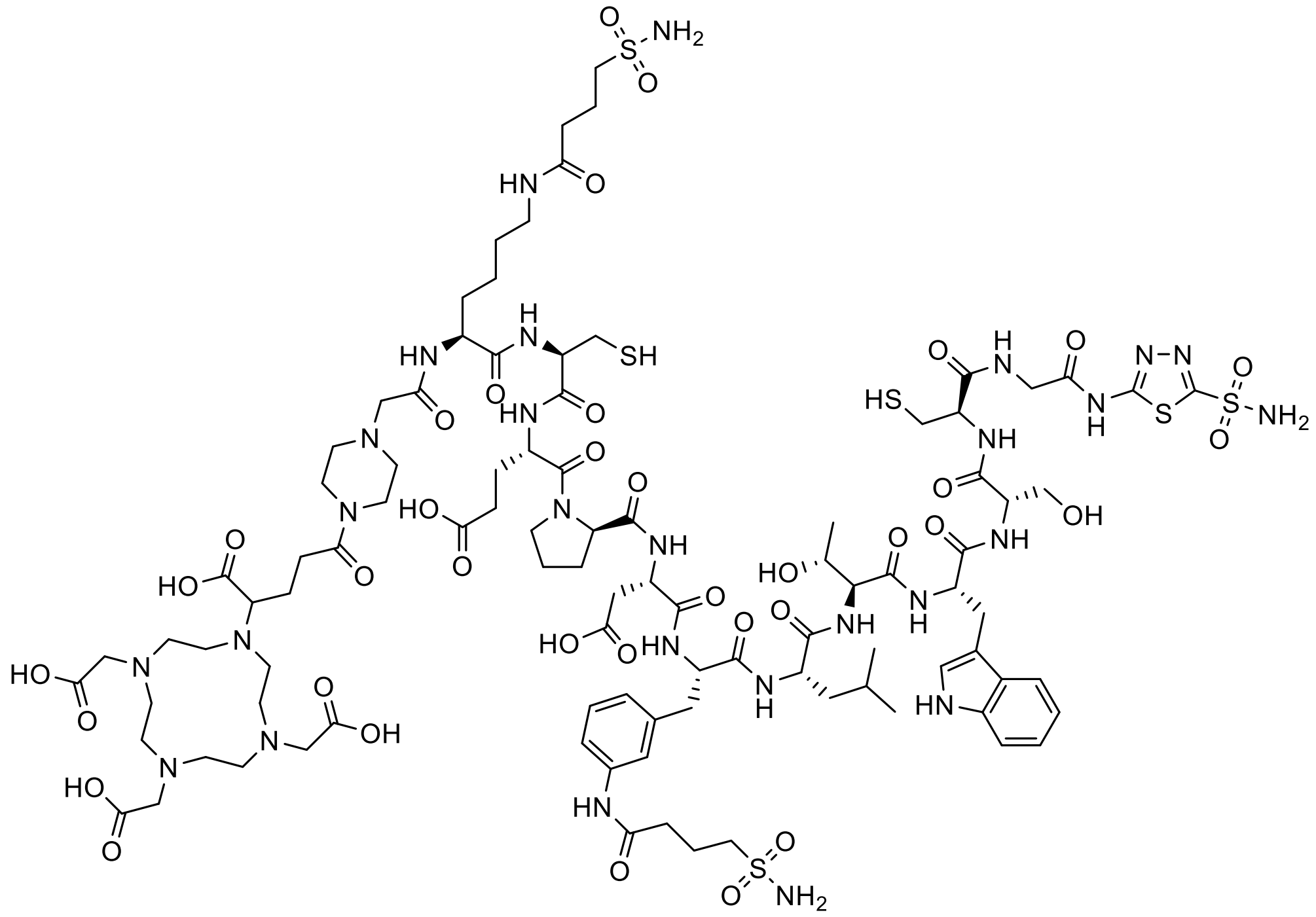

Compound 2

2,2',2''-(10-(4-(4-(2-(((S)-1-(((R)-1-(((S)-1-((R)-2-(((5R,8S,11S,14S,17S,20S,23S)-11-((1H-indol-3-yl)methyl)-24-carboxy-14-((R)-1-hydroxyethyl)-8-(hydroxymethyl)-17-isobutyl-5-(mercaptomethyl)-1,4,7,10,13,16,19,22-octaoxo-1-((5-sulfamoyl-1,3,4-thiadiazol-2-yl)amino)-20-(3-(4-sulfamoylbutanamido)benzyl)-3,6,9,12,15,18,21-heptaazatetracosan-23-yl)carbamoyl)pyrrolidin-1-yl)-4-carboxy-1-oxobutan-2-yl)amino)-3-mercapto-1-oxopropan-2-yl)amino)-1-oxo-6-(4-sulfamoylbutanamido)hexan-2-yl)amino)-2-oxoethyl)piperazin-1-yl)-1-carboxy-4-oxobutyl)-1,4,7,10-tetraazacyclododecane-1,4,7-triyl)triacetic acid (Compound 2)

To a solution of **compound 1** (32.6 mg, 0.01 mmol) and 2-amino-*N*-(5-sulfamoyl-1,3,4-thiadiazol-2-yl)acetamide (7.1 mg, 0.03 mmol, 3.0 equiv) in anhydrous DMF (1.5 mL) were added HOOBt (4.9 mg, 0.03 mmol, 3.0 equiv) and NMM (3.3 µL, 0.03 mmol, 3.0 equiv) as coupling reagents, followed by EDCI (5.7 mg, 0.03 mmol, 3.0 equiv) in DCM (0.5 mL). The mixture was stirred at room temperature for 12 h. The reaction mixture was concentrated under reduced pressure to remove DCM and most of the DMF. 95% TFA in H_2O (2 mL) was used to strip off Boc, tBu and Trt protecting groups for 2 h. The TFA solution was then concentrated under a gentle stream of nitrogen, and the crude product was precipitated by adding cold diethyl ether (10 mL). The precipitate was collected by centrifugation, washed twice with cold diethyl ether, and dried under vacuum. The crude product was then purified using HPLC on a C18 column obtaining **compound 2** as a white powder (11.0 mg, yield approximately 6.5%). MS (m/z): calced for $C_{96}H_{145}N_{27}O_{36}S_6$ $[M + 2H]^{2+}$, 1222.9, found, 1224.2.

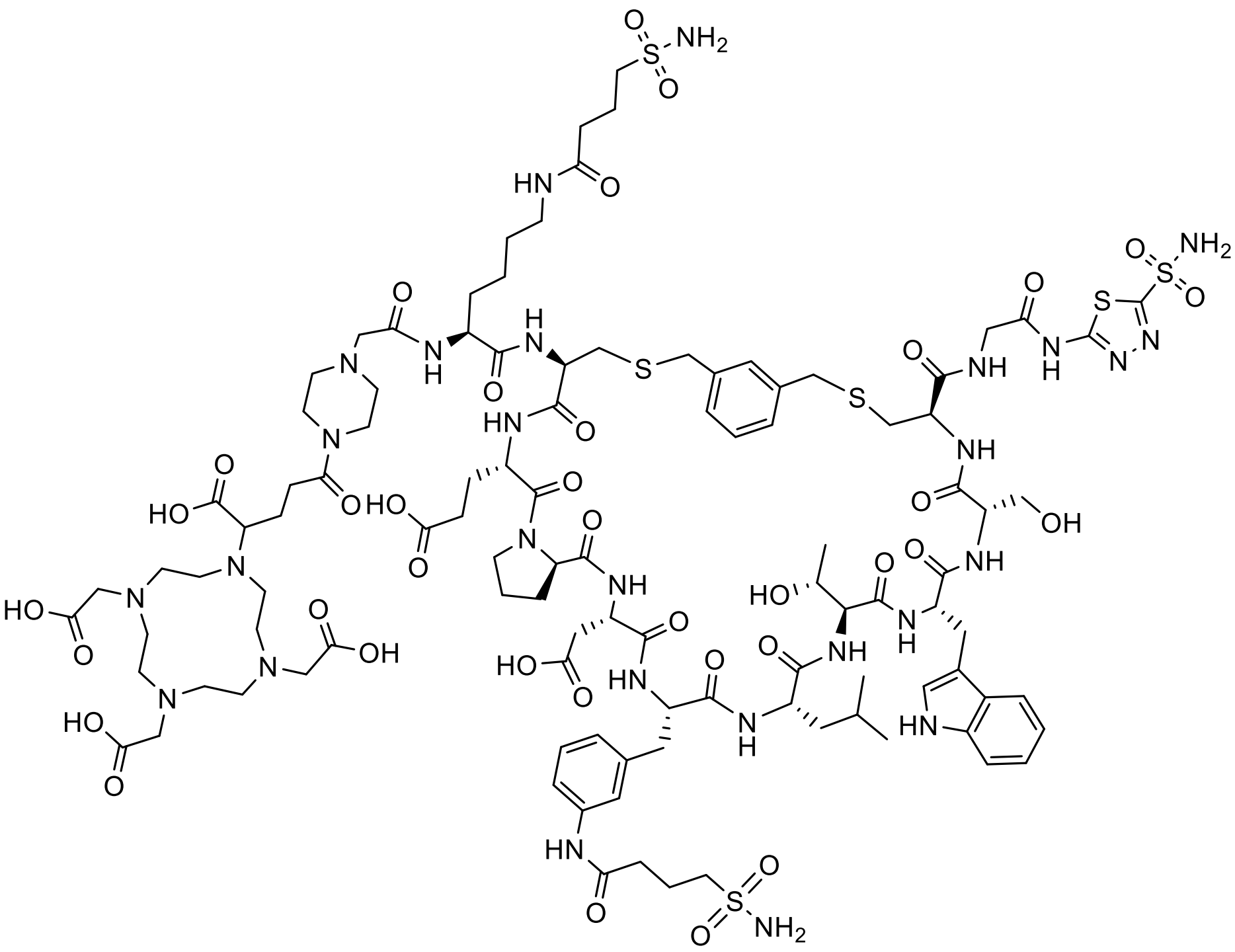

ZH1

2,2',2''-(10-(4-(4-(2-(((S)-1-(((12R,3S,6R,14R,17S,20S,23S,26S,29S,32S)-20-((1H-indol-3-yl)methyl)-3-(2-carboxyethyl)-32-(carboxymethyl)-23-((R)-1-hydroxyethyl)-17-(hydroxymethyl)-26-isobutyl-2,5,16,19,22,25,28,31,34-nonaoxo-14-((2-oxo-2-((5-sulfamoyl-1,3,4-thiadiazol-2-yl)amino)ethyl)carbamoyl)-29-(3-(4-sulfamoylbutanamido)benzyl)-8,12-dithia-4,15,18,21,24,27,30,33-octaaza-1(1,2)-pyrrolidina-10(1,3)-benzenacyclotetratriacontaphane-6-yl)amino)-1-oxo-6-(4-sulfamoylbutanamido)hexan-2-yl)amino)-2-oxoethyl)piperazin-1-yl)-1-carboxy-4-oxobutyl)-1,4,7,10-tetraazacyclododecane-1,4,7-triyl)triacetic acid (ZH1)

To a solution of **compound 2** (17.2 mg, 0.007 mmol) and 1,3-bis(bromomethyl)benzene (5.5 mg, 0.021 mmol, 3.0 equiv) in DMF (2 mL) was added a solution of NH_4HCO_3 in MeCN/H_2O to mediate the cyclization. The mixture was stirred at room temperature for 2 h. The reaction mixture was diluted with H_2O (10 mL) and lyophilized to remove volatile solvents (DMF, MeCN, and NH_4HCO_3). The crude product was then purified using HPLC on a C18 column obtaining **ZH1** as a white powder (5.7 mg, yield approximately 2.1%). MS (m/z): calced for $C_{104}H_{151}N_{27}O_{36}S_6$ $[M + 2H]^{2+}$, 1274.0, found, 1274.9.

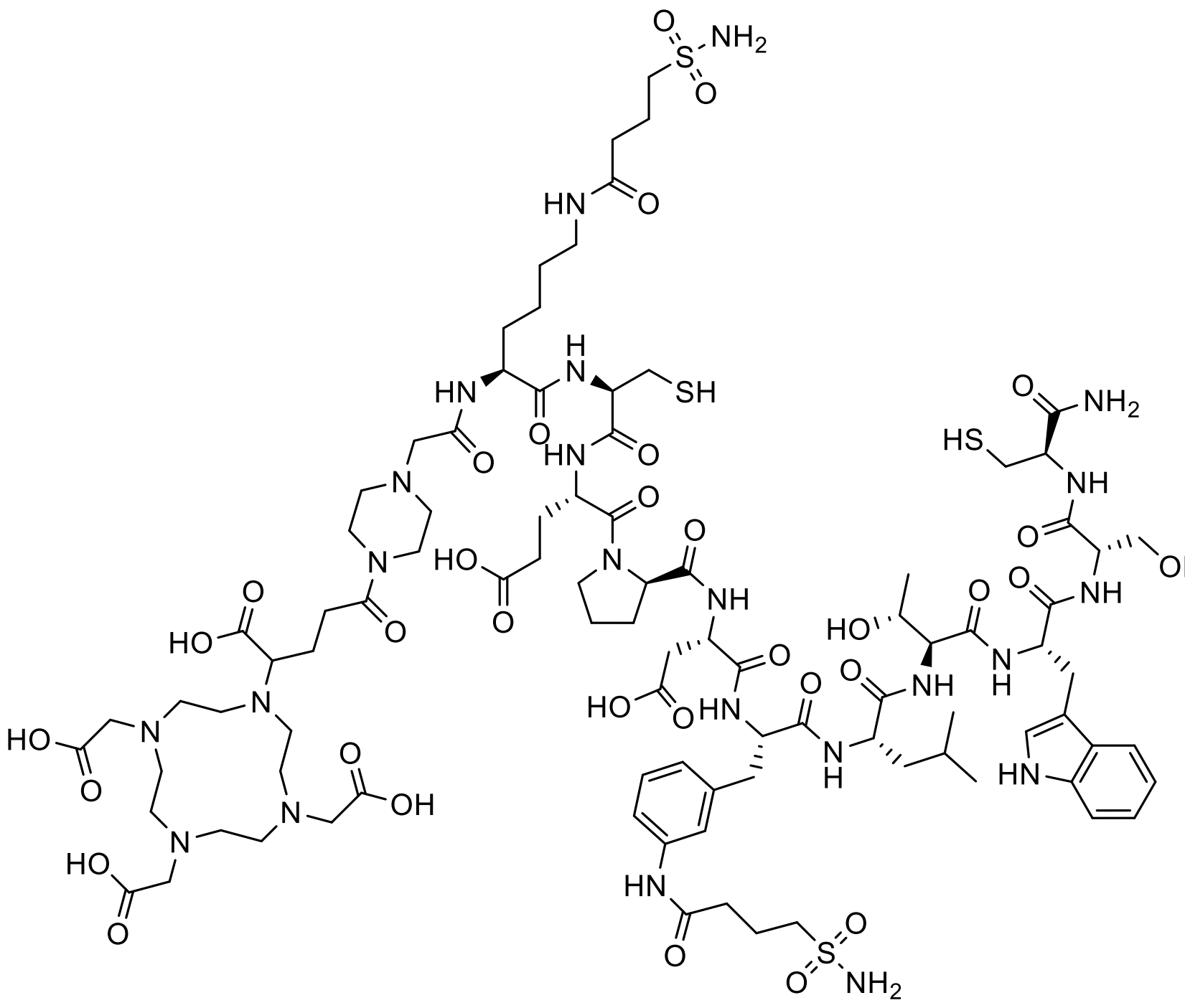

Compound 3

2,2',2''-(10-(4-(4-(2-(((S)-1-(((R)-1-(((S)-1-((R)-2-(((2R,5S,8S,11S,14S,17S,20S)-8-((1H-indol-3-yl)methyl)-1-amino-21-carboxy-11-((R)-1-hydroxyethyl)-5-(hydroxymethyl)-14-isobutyl-2-(mercaptomethyl)-1,4,7,10,13,16,19-heptaoxo-17-(3-(4-sulfamoylbutanamido)benzyl)-3,6,9,12,15,18-hexaazahenicosan-20-yl)carbamoyl)pyrrolidin-1-yl)-4-carboxy-1-oxobutan-2-yl)amino)-3-mercapto-1-oxopropan-2-yl)amino)-1-oxo-6-(4-sulfamoylbutanamido)hexan-2-yl)amino)-2-oxoethyl)piperazin-1-yl)-1-carboxy-4-oxobutyl)-1,4,7,10-tetraazacyclododecane-1,4,7-triyl)triacetic acid (Compound 3)

To a solution of **precursor peptide resin 2** (20.8 mg, containing 0.01 mmol of peptide) and 4-sulfamoylbutyric acid (5.0 mg, 0.03 mmol, 3.0 equiv) in anhydrous DMF (1.5 mL) were added HATU (3.8 mg, 0.01 mmol, 1.0 equiv) and DIPEA (5.2 μL, 0.03 mmol, 3.0 equiv). The mixture was stirred at room temperature for 2 h to complete the coupling reaction. The resin was then washed with DMF (3 × 2 mL) and DCM (3 × 2 mL) to remove excess reagents and by-products. To cleave the peptide from the Rink Amide MBHA resin and simultaneously remove all acid-labile protecting groups (Boc, tBu, Trt), the dried resin was treated with 95% TFA in H_2O (v/v, 2 mL) at room temperature for 2 h. The cleavage cocktail was collected by filtration, and the resin was washed with TFA (2 × 0.5 mL). The combined filtrate was concentrated under a gentle stream of nitrogen to afford the crude peptide. The crude product was then purified using HPLC on a C18 column obtaining **compound 3** (12.7 mg, yield approximately 17.8%). MS (m/z): calced for $C_{92}H_{141}N_{23}O_{33}S_4$ $[M + 2H]^{2+}$, 1112.9, found, 1113.3.

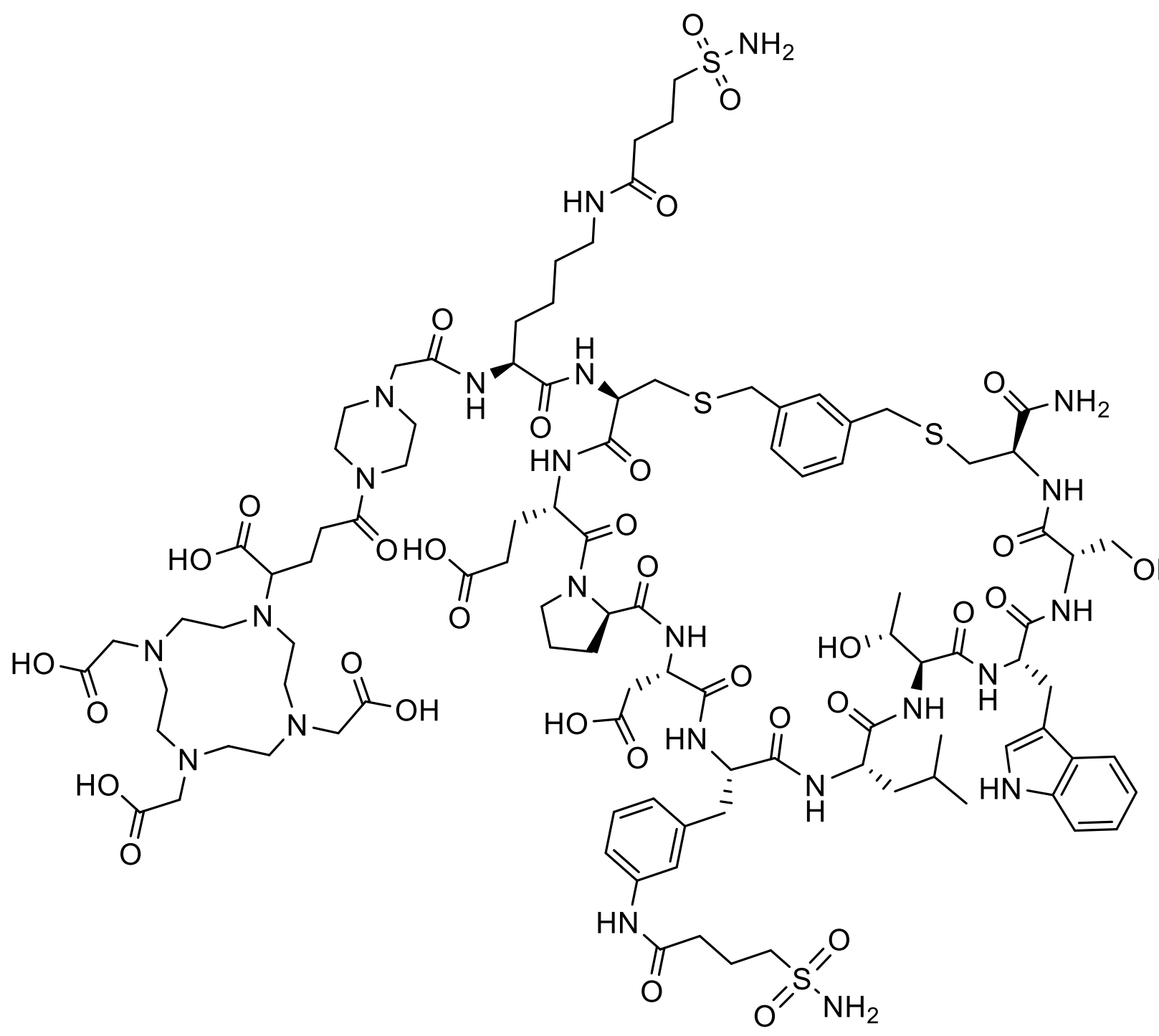

ZH2

2,2',2''-(10-(4-(4-(2-(((S)-1-(((12R,3S,6R,14R,17S,20S,23S,26S,29S,32S)-20-((1H-indol-3-yl)methyl)-14-carbamoyl-3-(2-carboxyethyl)-32-(carboxymethyl)-23-((R)-1-hydroxyethyl)-17-(hydroxymethyl)-26-isobutyl-2,5,16,19,22,25,28,31,34-nonaoxo-29-(3-(4-sulfamoylbutanamido)benzyl)-8,12-dithia-4,15,18,21,24,27,30,33-octaaza-1(1,2)-pyrrolidina-10(1,3)-benzenacyclotetratriacontaphane-6-yl)amino)-1-oxo-6-(4-sulfamoylbutanamido)hexan-2-yl)amino)-2-oxoethyl)piperazin-1-yl)-1-carboxy-4-oxobutyl)-1,4,7,10-tetraazacyclododecane-1,4,7-triyl)triacetic acid (ZH2)

To a solution of **compound 3** (18.0 mg, 0.008 mmol) and 1,3-bis(bromomethyl)benzene (6.4 mg, 0.024 mmol, 3.0 equiv) in DMF (2 mL) was added a freshly prepared mixture of NH_4HCO_3 solution in $MeCN/H_2O$ to promote cyclization. The resulting biphasic mixture was stirred at room temperature for 2 h. After completion, the reaction mixture was diluted with H_2O (10 mL) and lyophilized to remove volatile solvents (DMF, MeCN, and NH_4HCO_3). The crude product was then purified using HPLC on a C18 column obtaining **ZH2** as a white powder (7.4 mg, yield approximately 7.0%). MS (m/z): calced for $C_{100}H_{147}N_{23}O_{33}S_4$ $[M + 3H]^{3+}$, 776.3, found, 777.4.

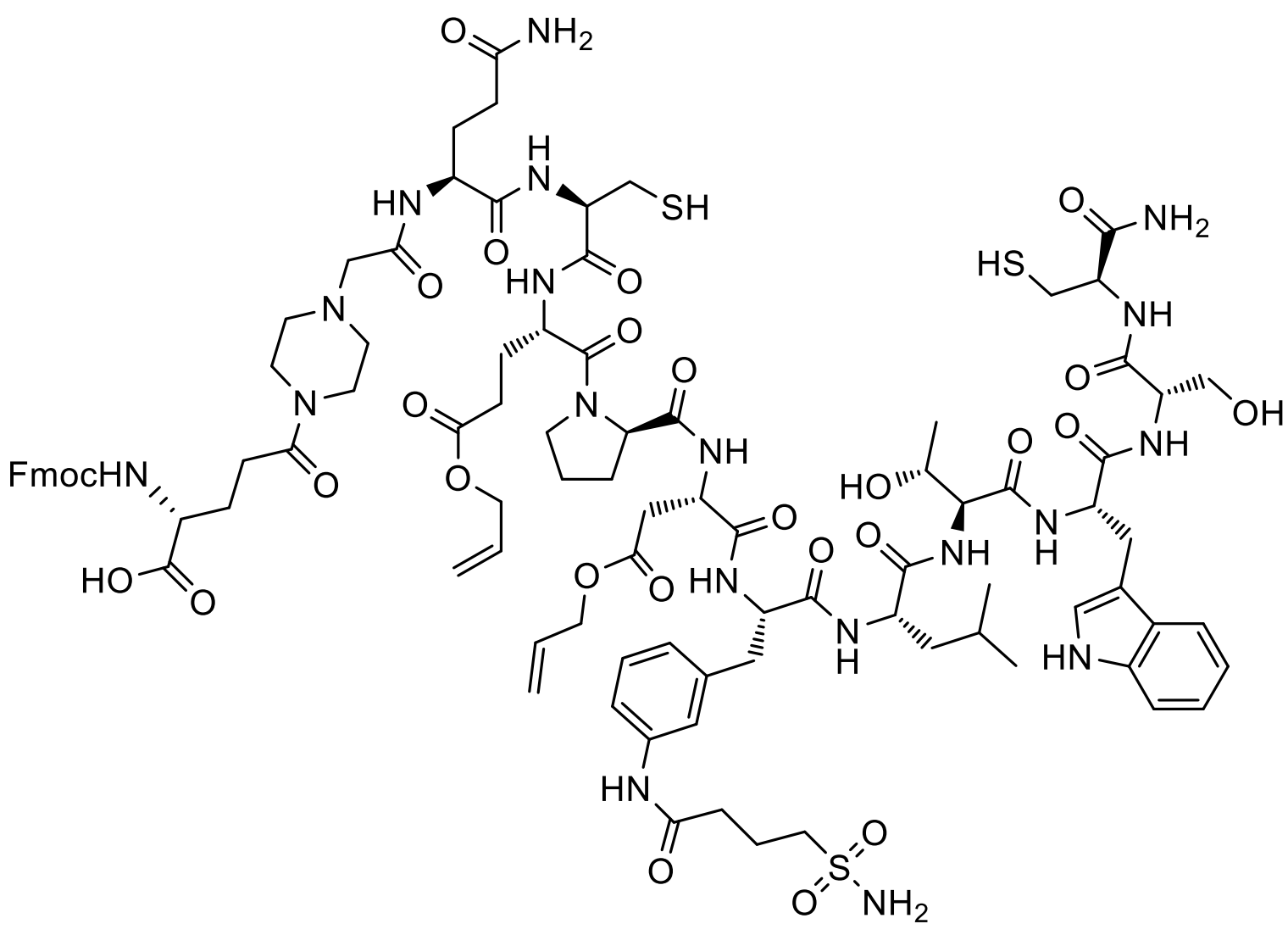

Compound 4

(R)-5-(4-((4S,7R,10S)-10-((R)-2-(((2R,5S,8S,11S,14S,17S,20S)-8-((1H-indol-3-yl)methyl)-1-amino-11-((R)-1-hydroxyethyl)-5-(hydroxymethyl)-14-isobutyl-2-(mercaptomethyl)-1,4,7,10,13,16,19,22-octaoxo-17-(3-(4-sulfamoylbutanamido)benzyl)-23-oxa-3,6,9,12,15,18-hexaazahexacos-25-en-20-yl)carbamoyl)pyrrolidine-1-carbonyl)-4-(3-amino-3-oxopropyl)-7-(mercaptomethyl)-2,5,8,13-tetraoxo-14-oxa-3,6,9-triazaheptadec-16-en-1-yl)piperazin-1-yl)-2-((((9H-fluoren-9-yl)methoxy)carbonyl)amino)-5-oxopentanoic acid (Compound 4)

To a solution of **Resin B** (17.0 mg, containing 0.01 mmol of peptide) and Fmoc-Glu-OtBu (12.8 mg, 0.03 mmol, 3.0 equiv) in DMF (1.5 mL) was added a coupling group of DIC (5.7 μL, 0.03 mmol, 3.0 equiv) and HOBt (4.1 mg, 0.03 mmol, 3.0 equiv). The mixture was stirred at room temperature for 2 h. The resin was then washed with DMF (3 × 2 mL) and DCM (3 × 2 mL) to remove excess reagents and by-products. To cleave the peptide from the Rink Amide MBHA resin and simultaneously remove all acid-labile protecting groups (including OtBu, Boc, Trt, etc.), the dried resin was treated with 95% TFA in H_2O (v/v, 2 mL) at room temperature for 2 h. The cleavage cocktail was collected by filtration, and the resin was washed with TFA (2 × 0.5 mL). The combined filtrate was concentrated under a gentle stream of nitrogen to afford the crude peptide. The crude product was then purified using HPLC on a C18 column obtaining **compound 4** (12.3 mg, yield approximately 18.8%). MS (m/z): calced for $C_{94}H_{125}N_{19}O_{27}S_3$ $[M + 2H]^{2+}$, 1024.9, found, 1024.9.

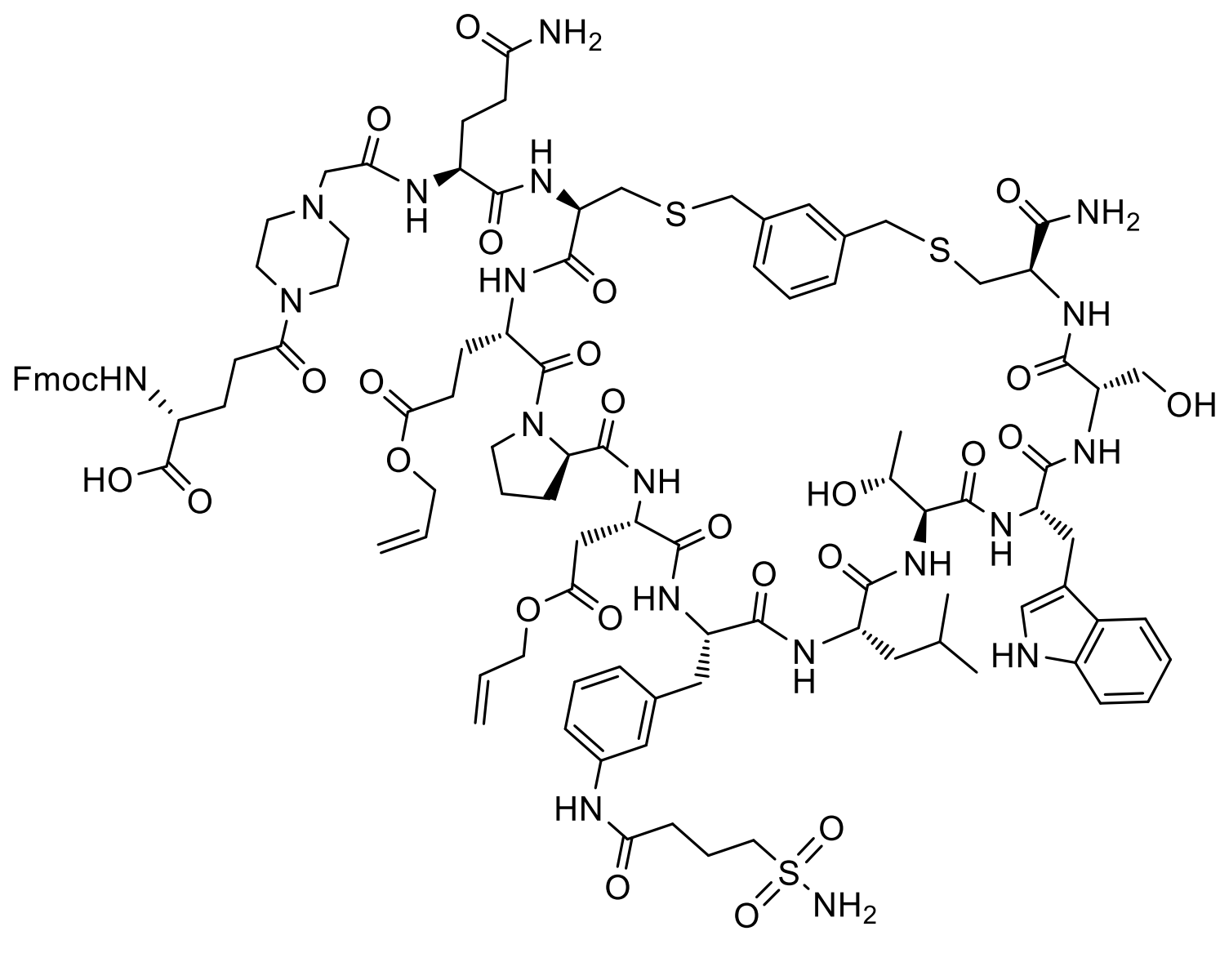

Compound 5

(R)-5-(4-(2-(((S)-1-(((12R,3S,6R,14R,17S,20S,23S,26S,29S,32S)-20-((1H-indol-3-yl)methyl)-32-(2-(allyloxy)-2-oxoethyl)-3-(3-(allyloxy)-3-oxopropyl)-14-carbamoyl-23-((R)-1-hydroxyethyl)-17-(hydroxymethyl)-26-isobutyl-2,5,16,19,22,25,28,31,34-nonaoxo-29-(3-(4-sulfamoylbutanamido)benzyl)-8,12-dithia-4,15,18,21,24,27,30,33-octaaza-1(1,2)-pyrrolidina-10(1,3)-benzenacyclotetratriacontaphane-6-yl)amino)-5-amino-1,5-dioxopentan-2-yl)amino)-2-oxoethyl)piperazin-1-yl)-2-((((9H-fluoren-9-yl)methoxy)carbonyl)amino)-5-oxopentanoic acid (Compound 5)

To a solution of **compound 4** (20.5 mg, 0.01 mmol) and 1,3-bis(bromomethyl)benzene (7.9 mg, 0.03 mmol, 3.0 equiv) in DMF (1.5 mL) was added a freshly prepared mixture of NH_4HCO_3 in MeCN/H_2O to promote cyclization. The resulting mixture was stirred at room temperature for 2 h. After completion, the reaction mixture was diluted with H_2O (10 mL) and lyophilized to remove volatile solvents (DMF, MeCN, and NH_4HCO_3). The crude product was then purified using HPLC on a C18 column obtaining **compound 5** (7.6 mg, yield approximately 6.6%). MS (m/z): calced for $C_{102}H_{131}N_{19}O_{27}S_3$ $[M + 2H]^{2+}$, 1075.9, found, 1076.4

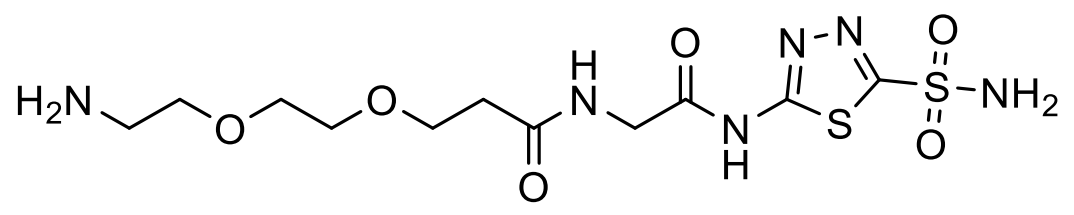

Compound 6

3-(2-(2-aminoethoxy)ethoxy)-N-(2-oxo-2-((5-sulfamoyl-1,3,4-thiadiazol-2-yl)amino)ethyl)propanamide (Compound 6)

To a solution of Fmoc-N-amido-PEG2-acid (4.0 mg, 0.01 mmol, 1.0 equiv) and 2-amino-N-(5-sulfamoyl-1,3,4-thiadiazol-2-yl)acetamide (2.4 mg, 0.01 mmol, 1.0 equiv) in anhydrous DMF (0.5 mL) were added HOOBt (1.5 mg, 0.01 mmol, 1.0 equiv), NMM (1.0 μL, 0.01 mmol, 1.0 equiv), and EDCI (1.7 mg, 0.01 mmol, 1.0 equiv) in DCM (0.2 mL). After completion of the coupling reaction, the solvent was removed under reduced pressure. The residue was treated with 20% piperidine in DMF (1.5 mL) at room temperature for 30 min to remove the Fmoc protecting group. The reaction mixture was then concentrated under a gentle stream of nitrogen, and the crude product was precipitated by adding cold diethyl ether (5 mL). The precipitate was collected by centrifugation, washed twice with cold diethyl ether, and dried under vacuum. The crude product was then purified using HPLC on a C18 column obtaining **compound 6** (1.6 mg, yield approximately 40.0%). MS (m/z): calced for $C_{11}H_{20}N_6O_6S_2$ $[M + H]^+$, 397.1, found, 397.2.

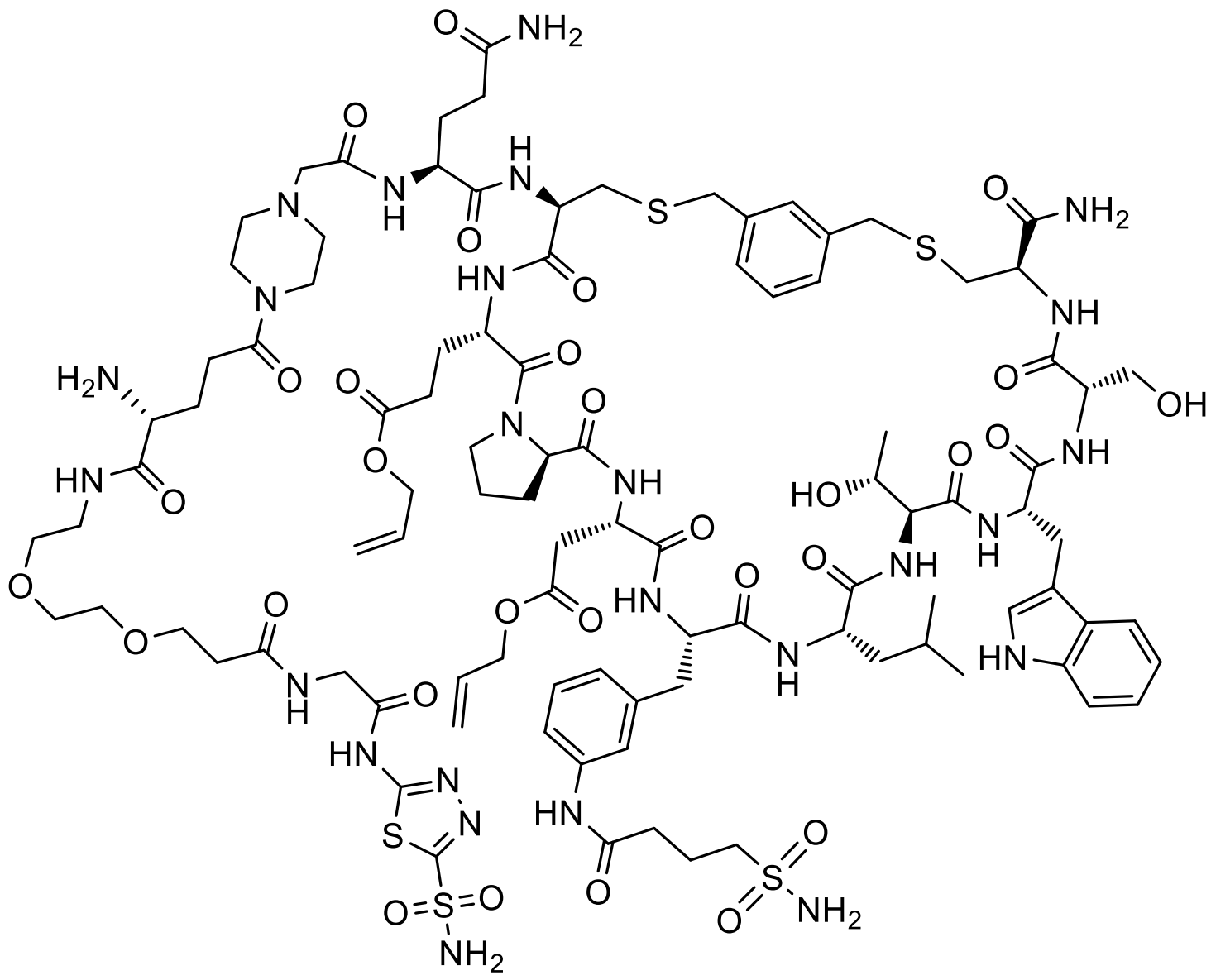

Compound 7

Allyl 3-((12R,3S,6R,14R,17S,20S,23S,26S,29S,32S)-20-((1H-indol-3-yl)methyl)-32-(2-(allyloxy)-2-oxoethyl)-6-((S)-5-amino-2-(2-(4-((R)-15-amino-1,4,14-trioxo-1-((5-sulfamoyl-1,3,4-thiadiazol-2-yl)amino)-7,10-dioxa-3,13-diazaoctadecan-18-oyl)piperazin-1-yl)acetamido)-5-oxopentanamido)-14-carbamoyl-23-((R)-1-hydroxyethyl)-17-(hydroxymethyl)-26-isobutyl-2,5,16,19,22,25,28,31,34-nonaoxo-29-(3-(4-sulfamoylbutanamido)benzyl)-8,12-dithia-4,15,18,21,24,27,30,33-octaaza-1(1,2)-pyrrolidina-10(1,3)-benzenacyclotetratriacontaphane-3-yl)propanoate (Compound 7)

To a solution of **compound 5** (11.4 mg, 0.006 mmol, containing a free carboxyl group) and **compound 6** (2.4 mg, 0.006 mmol, 1.0 equiv) in anhydrous DMF (1.0 mL) were added HOOBt (1.0 mg, 0.006 mmol, 1.0 equiv), NMM (0.66 μL, 0.006 mmol, 1.0 equiv), and EDCI (1.15 mg, 0.006 mmol, 1.0 equiv) in DCM (0.3 mL). The mixture was stirred at room temperature for 12 h. After completion of the coupling reaction, the solvent was removed under reduced pressure. The residue was treated with 20% piperidine in DMF (1.5 mL) at room temperature for 30 min to remove the Fmoc protecting group. The reaction mixture was then concentrated under a gentle stream of nitrogen, and the crude product was precipitated by adding cold diethyl ether (8 mL). The precipitate was collected by centrifugation, washed twice with cold diethyl ether, and dried under vacuum. The crude product was then purified using HPLC on a C18 column obtaining **compound 7** (3.1 mg, yield approximately 3.4%). MS (m/z): calced for $C_{98}H_{139}N_{25}O_{30}S_5$ $[M + 2H]^{2+}$, 1153.9, found, 1153.9.

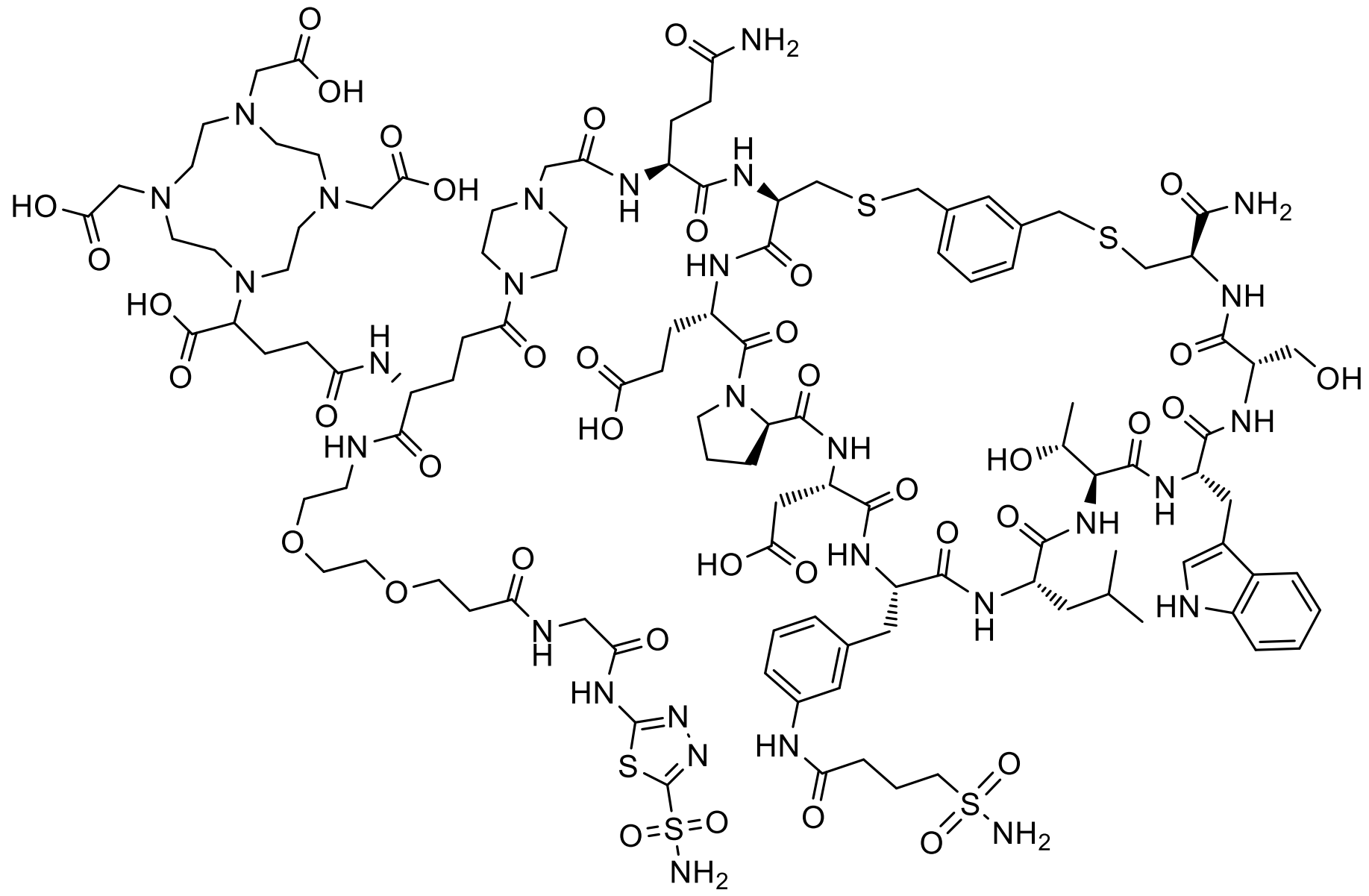

ZH3

2,2',2''-(10-((15R)-15-(3-(4-(2-(((S)-1-(((12R,3S,6R,14R,17S,20S,23S,26S,29S,32S)-20-((1H-indol-3-yl)methyl)-14-carbamoyl-3-(2-carboxyethyl)-32-(carboxymethyl)-23-((R)-1-hydroxyethyl)-17-(hydroxymethyl)-26-isobutyl-2,5,16,19,22,25,28,31,34-nonaoxo-29-(3-(4-sulfamoylbutanamido)benzyl)-8,12-dithia-4,15,18,21,24,27,30,33-octaaza-1(1,2)-pyrrolidina-10(1,3)-benzenacyclotetratriacontaphane-6-yl)amino)-5-amino-1,5-dioxopentan-2-yl)amino)-2-oxoethyl)piperazin-1-yl)-3-oxopropyl)-20-carboxy-1,4,14,17-tetraoxo-1-((5-sulfamoyl-1,3,4-thiadiazol-2-yl)amino)-7,10-dioxa-3,13,16-triazaicosan-20-yl)-1,4,7,10-tetraazacyclododecane-1,4,7-triyl)triacetic acid (ZH3)

To a solution of **compound 7** (2.3 mg, 0.001 mmol) and DOTA-GA(tBu)$_4$ (2.1 mg, 0.003 mmol, 3.0 equiv) in anhydrous DMF (0.5 mL) were added HATU (0.38 mg, 0.001 mmol, 1.0 equiv) and DIEA (0.52 μL, 0.003 mmol, 3.0 equiv). The mixture was stirred at room temperature for 2 h. After the coupling reaction, the solvent was removed under reduced pressure. The residue was dissolved in DMF (0.5 mL), and then Pd(PPh$_3$)$_4$ (1.16 mg, 0.001 mmol, 1.0 equiv) and N-methylaniline (0.33 μL, 0.003 mmol, 3.0 equiv) were added. Then, the mixture was stirred at room temperature for 12 h to remove the OAll group. Finally, the residue was treated with 95% TFA in H_2O (v/v, 1 mL) at room temperature for 2 h to remove tBu protecting groups. The crude product was then purified using HPLC on a C18 column obtaining **ZH3** as a white powder (1.4 mg, yield approximately 1.8%). MS (m/z): calced for $C_{111}H_{161}N_{29}O_{39}S_5$ $[M + 2H]^{2+}$, 1343.0, found, 1344.1.

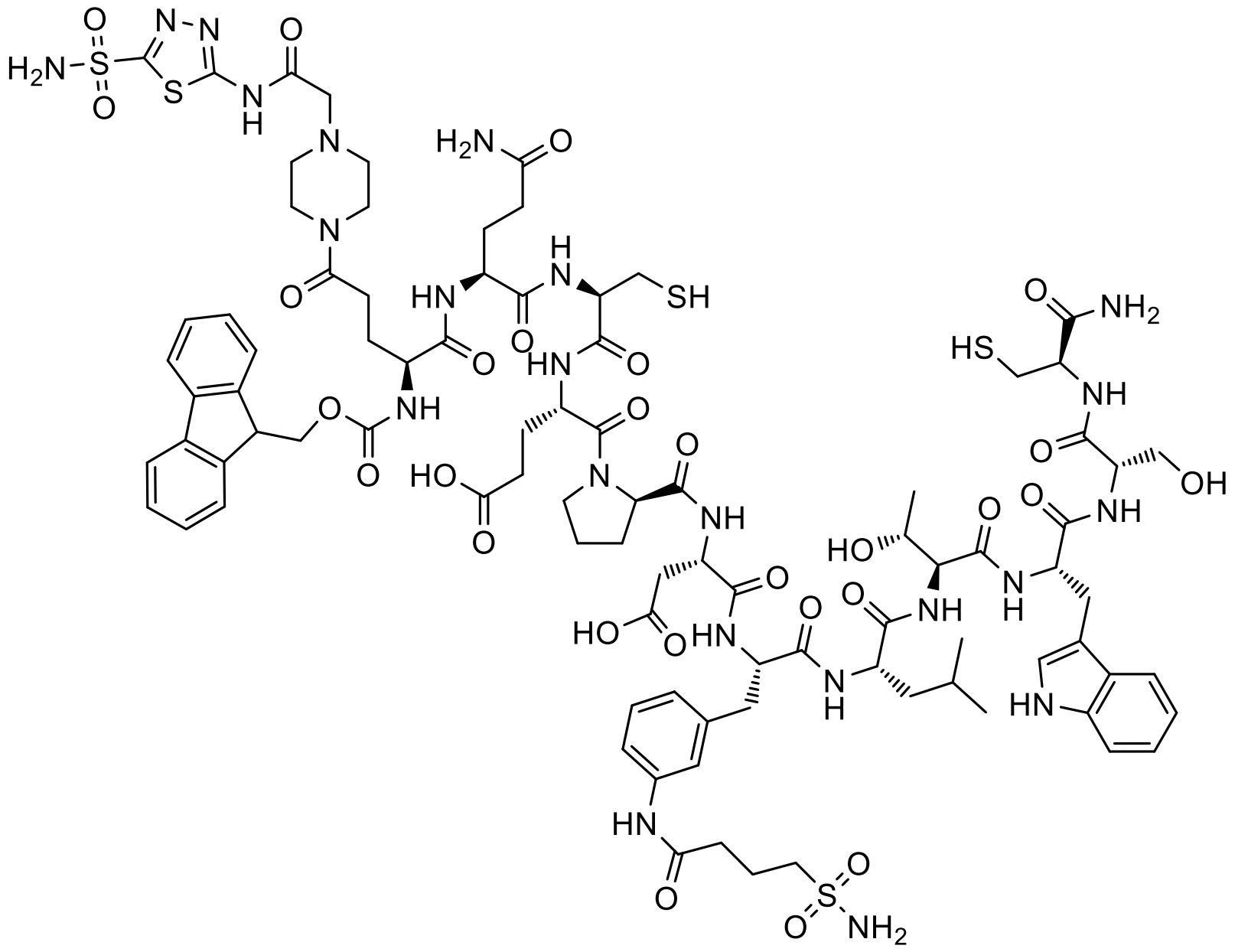

Compound 8

(2R,5S,8S,11S,14S,17S,20S)-8-((1H-indol-3-yl)methyl)-1-amino-20-((R)-1-((5S,8S,11R,14S)-8-(3-amino-3-oxopropyl)-14-(2-carboxyethyl)-1-(9H-fluoren-9-yl)-11-(mercaptomethyl)-3,6,9,12-tetraoxo-5-(3-oxo-3-(4-(2-oxo-2-((5-sulfamoyl-1,3,4-thiadiazol-2-yl)amino)ethyl)piperazin-1-yl)propyl)-2-oxa-4,7,10,13-tetraazapentadecan-15-oyl)pyrrolidine-2-carboxamido)-11-((R)-1-hydroxyethyl)-5-(hydroxymethyl)-14-isobutyl-2-(mercaptomethyl)-1,4,7,10,13,16,19-heptaoxo-17-(3-(4-sulfamoylbutanamido)benzyl)-3,6,9,12,15,18-hexaazadocosan-22-oic acid (Compound 8)

To a solution of **precursor peptide resin 3** (18.4 mg, 0.01 mmol) and 2-(piperazin-1-yl)-N-(5-sulfamoyl-1,3,4-thiadiazol-2-yl)acetamide (9.2 mg, 0.03 mmol, 3.0 equiv) in DMF (1.5 mL) was added HATU (3.8 mg, 0.01 mmol, 1.0 equiv) as coupling reagent and DIEA (5.2 µL, 0.03 mmol, 3.0 equiv) as base. The mixture was stirred at room temperature for 2 h to complete the coupling reaction. The resin was then washed with DMF (3 × 2 mL) and DCM (3 × 2 mL) to remove excess reagents and by-products. To cleave the peptide from the Rink Amide MBHA resin and simultaneously remove all acid-labile protecting groups (Boc, tBu, Trt, etc.), the dried resin was treated with 95% TFA in H_2O (v/v, 2 mL) at room temperature for 2 h. The cleavage cocktail was collected by filtration, and the resin was washed with TFA (2 × 0.5 mL). The crude peptide was then purified using HPLC on a C18 column obtaining **compound 8** (11.7 mg, yield approximately 17.2%). MS (m/z): calced for $C_{90}H_{119}N_{23}O_{28}S_5$ $[M + 2H]^{2+}$, 1065.9, found, 1066.1.

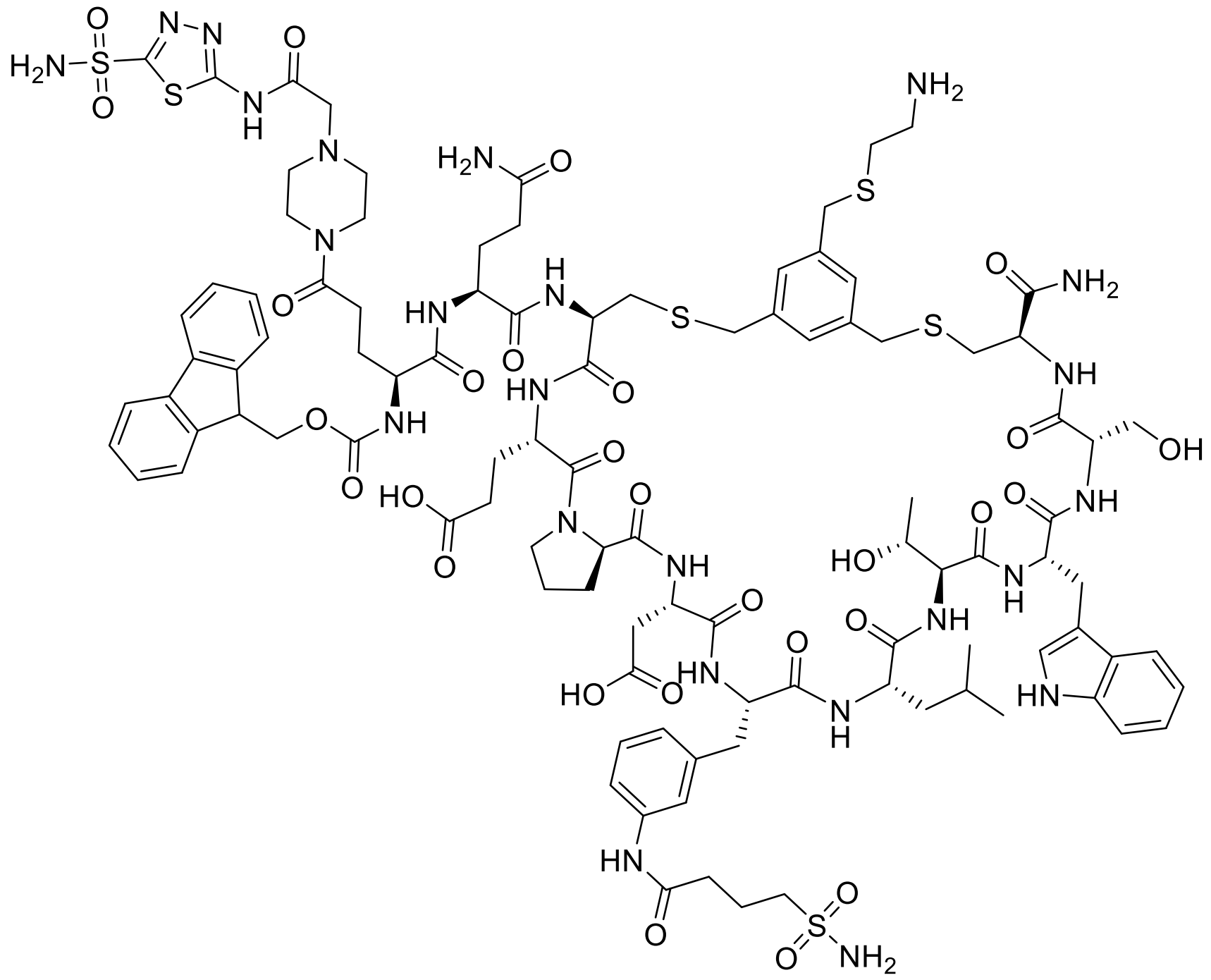

Compound 9

3-((12R,3S,6R,14R,17S,20S,23S,26S,29S,32S)-20-((1H-indol-3-yl)methyl)-6-((S)-2-((S)-2-((((9H-fluoren-9-yl)methoxy)carbonyl)amino)-5-oxo-5-(4-(2-oxo-2-((5-sulfamoyl-1,3,4-thiadiazol-2-yl)amino)ethyl)piperazin-1-yl)pentanamido)-5-amino-5-oxopentanamido)-105-(((2-aminoethyl)thio)methyl)-14-carbamoyl-32-(carboxymethyl)-23-((R)-1-hydroxyethyl)-17-(hydroxymethyl)-26-isobutyl-2,5,16,19,22,25,28,31,34-nonaoxo-29-(3-(4-sulfamoylbutanamido)benzyl)-8,12-dithia-4,15,18,21,24,27,30,33-octaaza-1(1,2)-pyrrolidina-10(1,3)-benzenacyclotetratriacontaphane-3-yl)propanoic acid (Compound 9)

To a solution of **compound 8** (21.3 mg, 0.01 mmol) in DMF (1.5 mL) were added 1,3,5-tris(bromomethyl)benzene (3.6 mg, 0.01 mmol, 1.0 equiv) and 2-aminoethane-1-thiol (0.8 mg, 0.0104 mmol, 1.04 equiv). Then a freshly prepared mixture of NH_4HCO_3 in MeCN/H_2O was added to promote cyclization. The resulting mixture was stirred at room temperature for 2 h. After completion, the reaction mixture was diluted with H_2O (10 mL) and lyophilized to remove volatile solvents (DMF, MeCN, and NH_4HCO_3). The crude product was then purified using HPLC on a C18 column obtaining **compound 9** (9.5 mg, yield approximately 7.0%). MS (m/z): calced for $C_{101}H_{132}N_{24}O_{28}S_6$ $[M + 2H]^{2+}$, 1161.4, found, 1161.9.

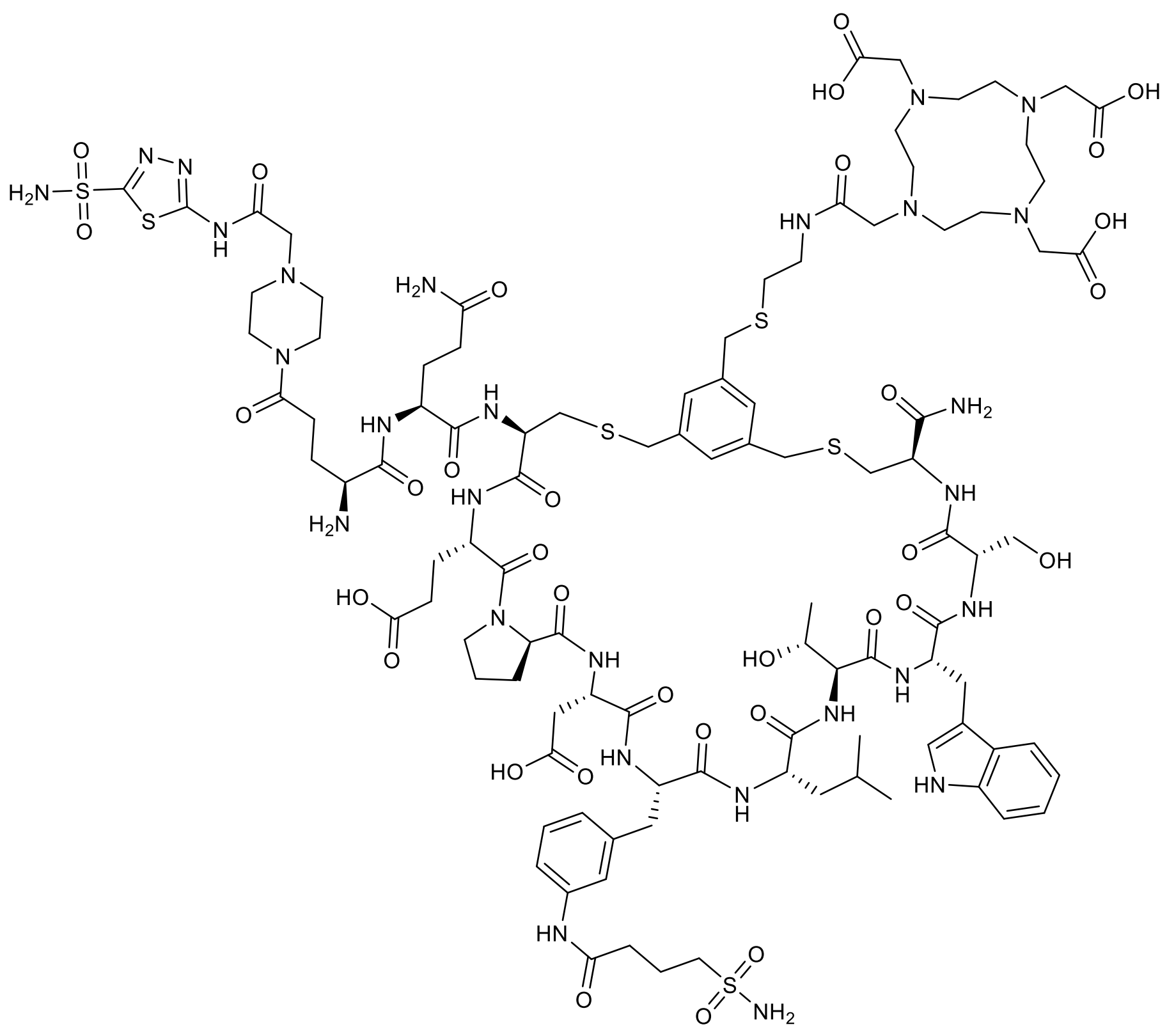

ZH4

2,2',2''-(10-(2-((2-((((12R,3S,6R,14R,17S,20S,23S,26S,29S,32S)-20-((1H-indol-3-yl)methyl)-6-((S)-2-((S)-2-((((9H-fluoren-9-yl)methoxy)carbonyl)amino)-5-oxo-5-(4-(2-oxo-2-((5-sulfamoyl-1,3,4-thiadiazol-2-yl)amino)ethyl)piperazin-1-yl)pentanamido)-5-amino-5-oxopentanamido)-14-carbamoyl-3-(2-carboxyethyl)-32-(carboxymethyl)-23-((R)-1-hydroxyethyl)-17-(hydroxymethyl)-26-isobutyl-2,5,16,19,22,25,28,31,34-nonaoxo-29-(3-(4-sulfamoylbutanamido)benzyl)-8,12-dithia-4,15,18,21,24,27,30,33-octaaza-1(1,2)-pyrrolidina-10(1,3)-benzenacyclotetratriacontaphane-105-yl)methyl)thio)ethyl)amino)-2-oxoethyl)-1,4,7,10-tetraazacyclododecane-1,4,7-triyl)triacetic acid (ZH4)

To a solution of **compound 9** (15 mg, 0.0065 mmol) and DOTA-NHS-ester (9.8 mg, 0.0195 mmol, 3.0 equiv) in anhydrous DMF (1.0 mL) were added HATU (2.5 mg, 0.0065 mmol, 1.0 equiv) and DIEA (3.4 µL, 0.0195 mmol, 3.0 equiv). The mixture was stirred at room temperature for 2 h. After the coupling reaction, the solvent was removed under reduced pressure. The residue was treated with 20% piperidine in DMF (1.5 mL) at room temperature for 2 h to remove pyrrolidine-2,5-dione groups. The reaction mixture was then concentrated under a gentle stream of nitrogen, and the crude product was precipitated by adding cold diethyl ether (8 mL). The precipitate was collected by centrifugation, washed twice with cold diethyl ether, and dried under vacuum. The crude product was then purified using HPLC on a C18 column obtaining **ZH4** as a white powder (8.3 mg, yield approximately 3.0%). MS (m/z): calced for $C_{102}H_{148}N_{28}O_{33}S_6$ $[M + 2H]^{2+}$, 1243.5, found, 1244.0.

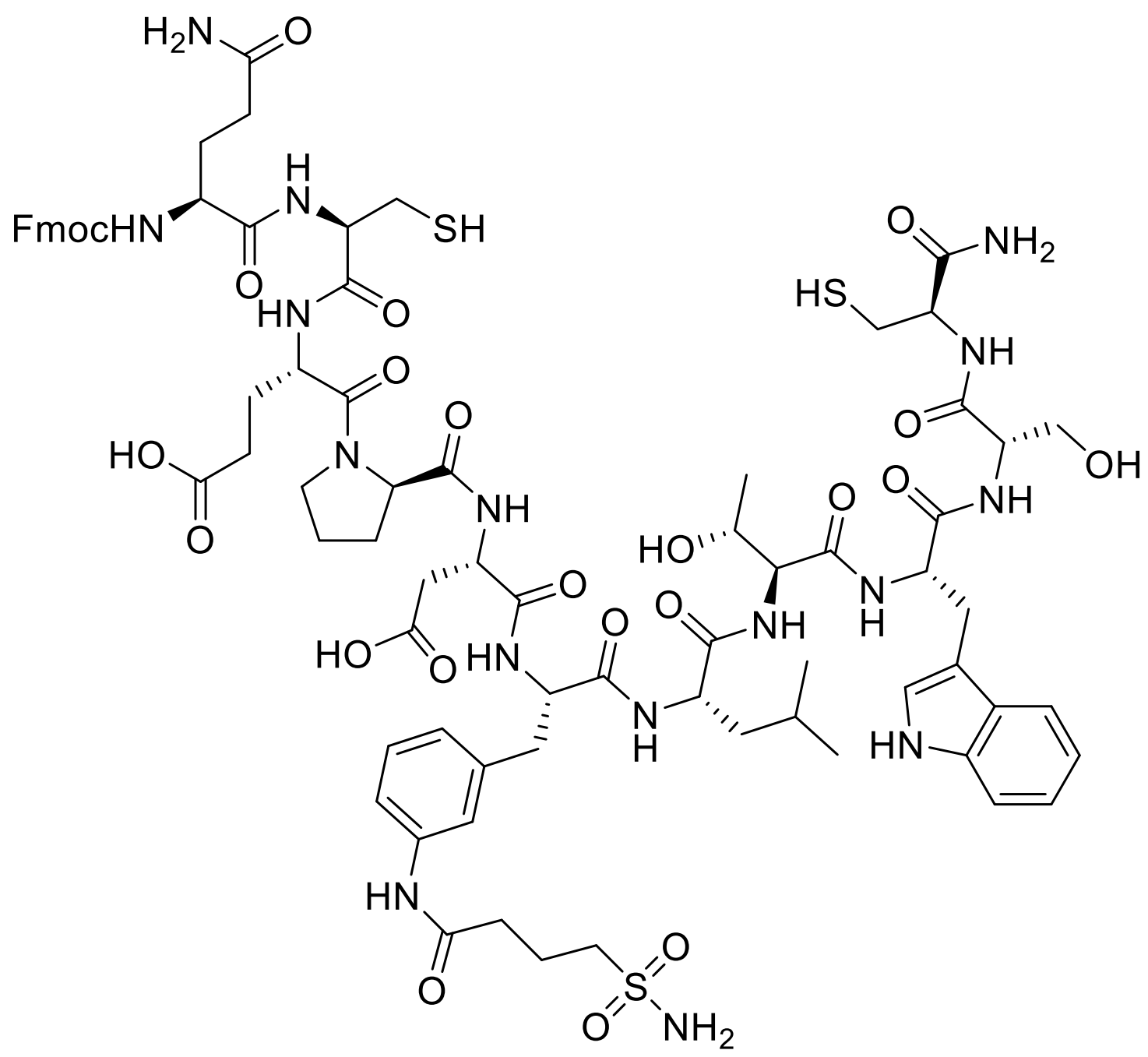

Compound 10

(2R,5S,8S,11S,14S,17S,20S)-8-((1H-indol-3-yl)methyl)-1-amino-20-((R)-1-((5S,8R,11S)-5-(3-amino-3-oxopropyl)-11-(2-carboxyethyl)-1-(9H-fluoren-9-yl)-8-(mercaptomethyl)-3,6,9-trioxo-2-oxa-4,7,10-triazadodecan-12-oyl)pyrrolidine-2-carboxamido)-11-((R)-1-hydroxyethyl)-5-(hydroxymethyl)-14-isobutyl-2-(mercaptomethyl)-1,4,7,10,13,16,19-heptaoxo-17-(3-(4-sulfamoylbutanamido)benzyl)-3,6,9,12,15,18-hexaazadocosan-22-oic acid (Compound 10)

To a solution of **Resin A** (13.6 mg, 0.01 mmol) and Fmoc-Gln(Trt)-OH (18.3 mg, 0.03 mmol, 3.0 equiv) in DMF (1.5 mL) was added a coupling group of DIC (4.7 µL, 0.03 mmol, 3.0 equiv) and HOBt (4.1 mg, 0.03 mmol, 3.0 equiv). The mixture was stirred at room temperature for 2 h. The resin was then washed with DMF (3 × 2 mL) and DCM (3 × 2 mL) to remove excess reagents and by-products. To cleave the peptide from the Rink Amide MBHA resin and simultaneously remove protecting groups, the dried resin was treated with 95% TFA in H_2O (v/v, 2 mL) at room temperature for 2 h. The cleavage cocktail was collected by filtration, and the resin was washed with TFA (2 × 0.5 mL). The combined filtrate was concentrated under a gentle stream of nitrogen to afford the crude peptide. The crude product was then purified using HPLC on a C18 column obtaining **compound 10** (8.8 mg, yield approximately 16.1%). MS (m/z): calced for $C_{77}H_{100}N_{16}O_{23}S_3$ $[M + 2H]^{2+}$, 857.3, found, 857.6.

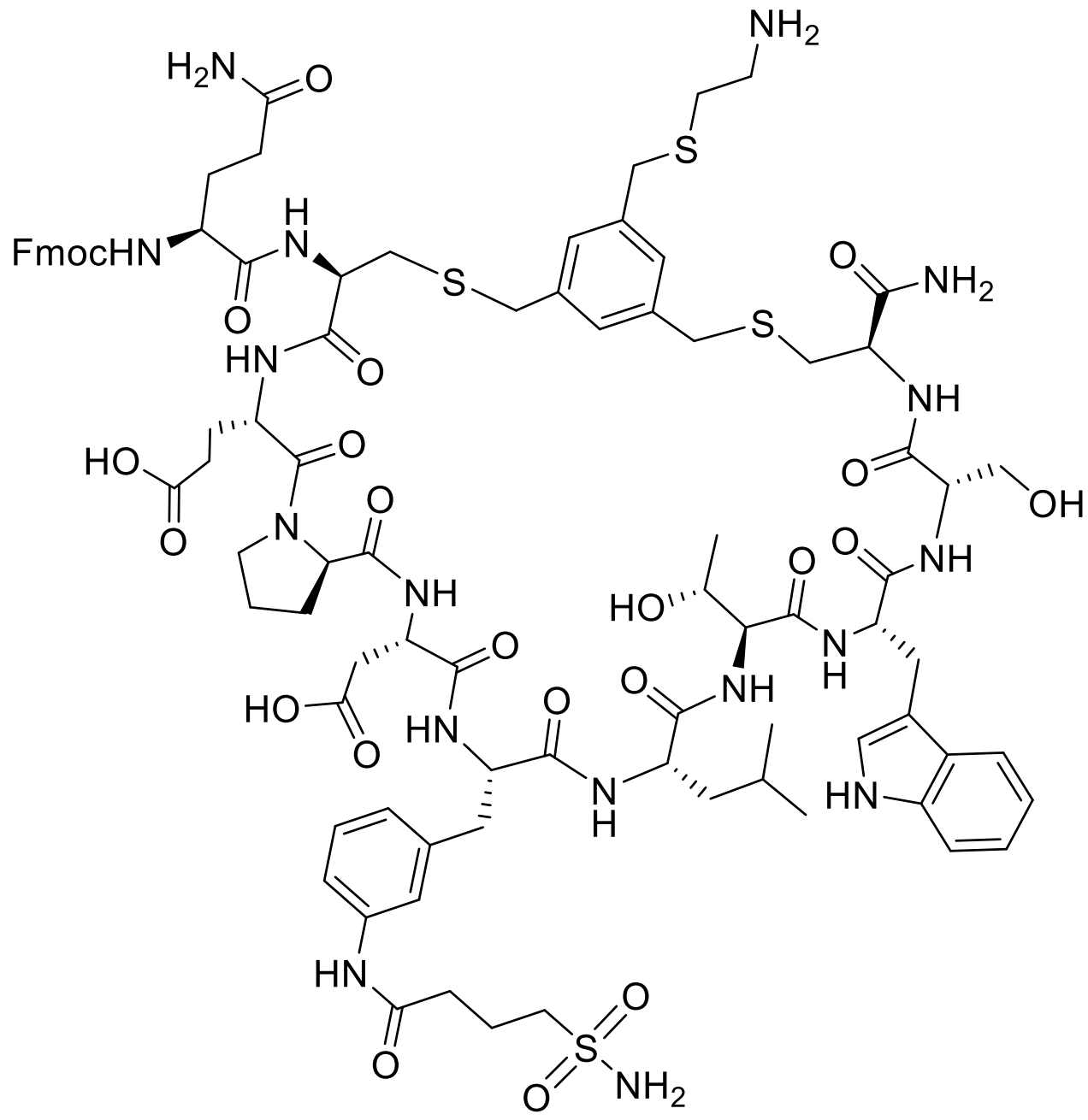

Compound 11

3-((12R,3S,6R,14R,17S,20S,23S,26S,29S,32S)-20-((1H-indol-3-yl)methyl)-6-((S)-2-((((9H-fluoren-9-yl)methoxy)carbonyl)amino)-5-amino-5-oxopentanamido)-105-(((2-aminoethyl)thio)methyl)-14-carbamoyl-32-(carboxymethyl)-23-((R)-1-hydroxyethyl)-17-(hydroxymethyl)-26-isobutyl-2,5,16,19,22,25,28,31,34-nonaoxo-29-(3-(4-sulfamoylbutanamido)benzyl)-8,12-dithia-4,15,18,21,24,27,30,33-octaaza-1(1,2)-pyrrolidina-10(1,3)-benzenacyclotetratriacontaphane-3-yl)propanoic acid (Compound 11)

To a solution of **compound 10** (17.1 mg, 0.01 mmol) in DMF (1.5 mL) were added 1,3,5-tris(bromomethyl)benzene (3.6 mg, 0.01 mmol, 1.0 equiv) and 2-aminoethane-1-thiol (0.8 mg, 0.01 mmol, 1.0 equiv). Then a freshly prepared mixture of NH_4HCO_3 in MeCN/H_2O was added to promote cyclization. The resulting mixture was stirred at room temperature for 2 h. After completion, the reaction mixture was diluted with H_2O (10 mL) and lyophilized to remove volatile solvents (DMF, MeCN, and NH_4HCO_3). The crude product was then purified using HPLC on a C18 column obtaining **compound 11** (7.3 mg, yield approximately 6.2%). MS (m/z): calced for $C_{88}H_{113}N_{17}O_{23}S_4$ $[M + 2H]^{2+}$, 952.9, found, 952.9.

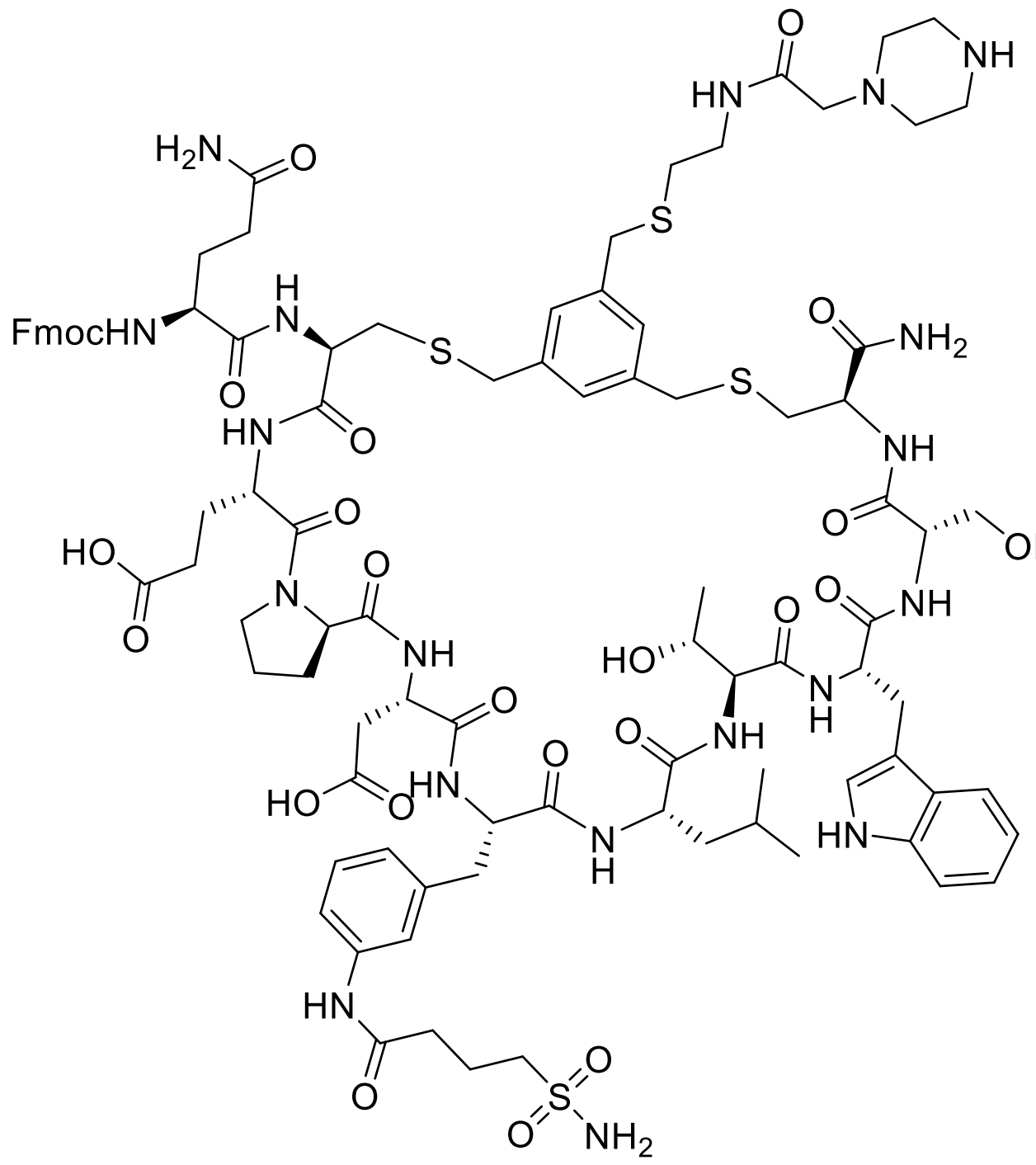

Compound 12

3-((12R,3S,6R,14R,17S,20S,23S,26S,29S,32S)-20-((1H-indol-3-yl)methyl)-6-((S)-2-((((9H-fluoren-9-yl)methoxy)carbonyl)amino)-5-amino-5-oxopentanamido)-14-carbamoyl-32-(carboxymethyl)-23-((R)-1-hydroxyethyl)-17-(hydroxymethyl)-26-isobutyl-2,5,16,19,22,25,28,31,34-nonaoxo-105-(((2-(2-(piperazin-1-yl)acetamido)ethyl)thio)methyl)-29-(3-(4-sulfamoylbutanamido)benzyl)-8,12-dithia-4,15,18,21,24,27,30,33-octaaza-1(1,2)-pyrrolidina-10(1,3)-benzenacyclotetratriacontaphane-3-yl)propanoic acid (Compound 12)

To a solution of **compound 11** (10.5 mg, 0.0055 mmol) and 4-Boc-1-piperazineacetic acid (4.0 mg, 0.0164 mmol, 3.0 equiv) in anhydrous DMF (1.0 mL) were added DCC (1.13 mg, 0.0055 mmol, 1.0 equiv), HOSu (0.63 mg, 0.0055 mmol, 1.0 equiv), and DIEA (1.15 μL, 0.0066 mmol, 1.2 equiv). The mixture was stirred at room temperature for 3 h. After the coupling reaction, the solvent was removed under reduced pressure. The residue was treated with 95% TFA in H_2O (v/v, 1.5 mL) at room temperature for 2 h to remove Boc protecting group. The reaction mixture was then concentrated under a gentle stream of nitrogen, and the crude product was precipitated by adding cold diethyl ether (8 mL). The precipitate was collected by centrifugation, washed twice with cold diethyl ether, and dried under vacuum. The crude product was then purified using HPLC on a C18 column obtaining **compound 12** (6.8 mg, yield approximately 3.8%). MS (m/z): calced for $C_{94}H_{123}N_{19}O_{24}S_4$ $[M + 2H]^{2+}$, 1015.9, found, 1015.9.

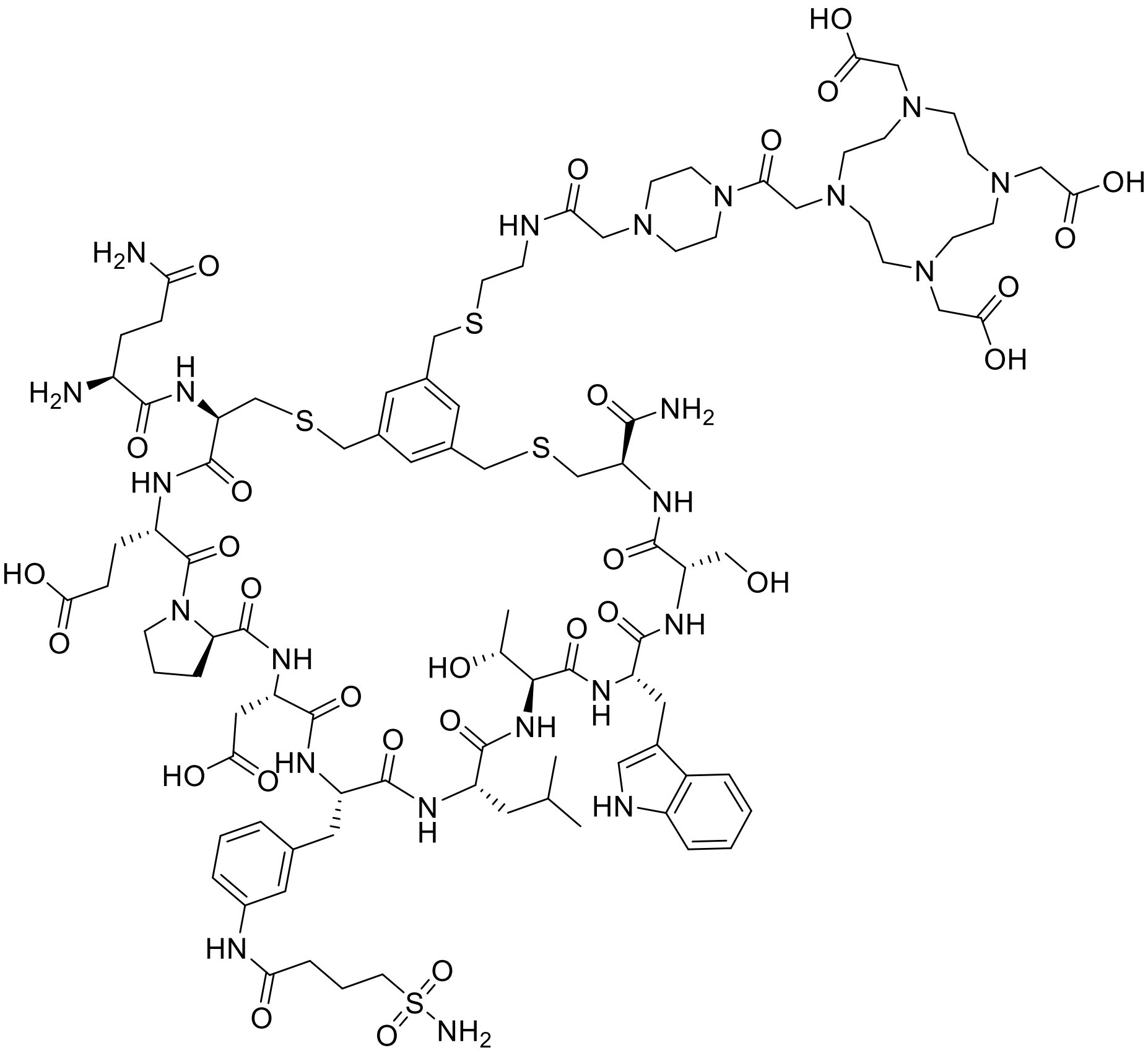

ZH5

2,2',2''-(10-(2-(4-(2-((2-((((12R,3S,6R,14R,17S,20S,23S,26S,29S,32S)-20-((1H-indol-3-yl)methyl)-14-carbamoyl-3-(2-carboxyethyl)-32-(carboxymethyl)-6-((S)-2,5-diamino-5-oxopentanamido)-23-((R)-1-hydroxyethyl)-17-(hydroxymethyl)-26-isobutyl-2,5,16,19,22,25,28,31,34-nonaoxo-29-(3-(4-sulfamoylbutanamido)benzyl)-8,12-dithia-4,15,18,21,24,27,30,33-octaaza-1(1,2)-pyrrolidina-10(1,3)-benzenacyclotetratriacontaphane-105-yl)methyl)thio)ethyl)amino)-2-oxoethyl)piperazin-1-yl)-2-oxoethyl)-1,4,7,10-tetraazacyclododecane-1,4,7-triyl)triacetic acid (ZH5)

To a solution of **compound 12** (6.5 mg, 0.0032 mmol) and DOTA-NHS-ester (4.8 mg, 0.0096 mmol, 3.0 equiv) in anhydrous DMF (1.0 mL) were added HATU (1.22 mg, 0.0032 mmol, 1.0 equiv) and DIEA (1.7 µL, 0.0096 mmol, 3.0 equiv). The mixture was stirred at room temperature for 2 h. After the coupling reaction, the solvent was removed under reduced pressure. The residue was treated with 20% piperidine in DMF (1.5 mL) at room temperature for 2 h to remove pyrrolidine-2,5-dione groups. The reaction mixture was then concentrated under a gentle stream of nitrogen, and the crude product was precipitated by adding cold diethyl ether (8 mL). The precipitate was collected by centrifugation, washed twice with cold diethyl ether, and dried under vacuum. The crude product was then purified using HPLC on a C18 column obtaining **ZH5** as a white powder (2.9 mg, yield approximately 1.3%). MS (m/z): calced for $C_{95}H_{139}N_{23}O_{29}S_4$ $[M + 2H]^{2+}$, 1098.0, found, 1098.8.

1.4. Spectroscopic and chromatogram data

The High-resolution mass spectrometry for the reference compound **1**.

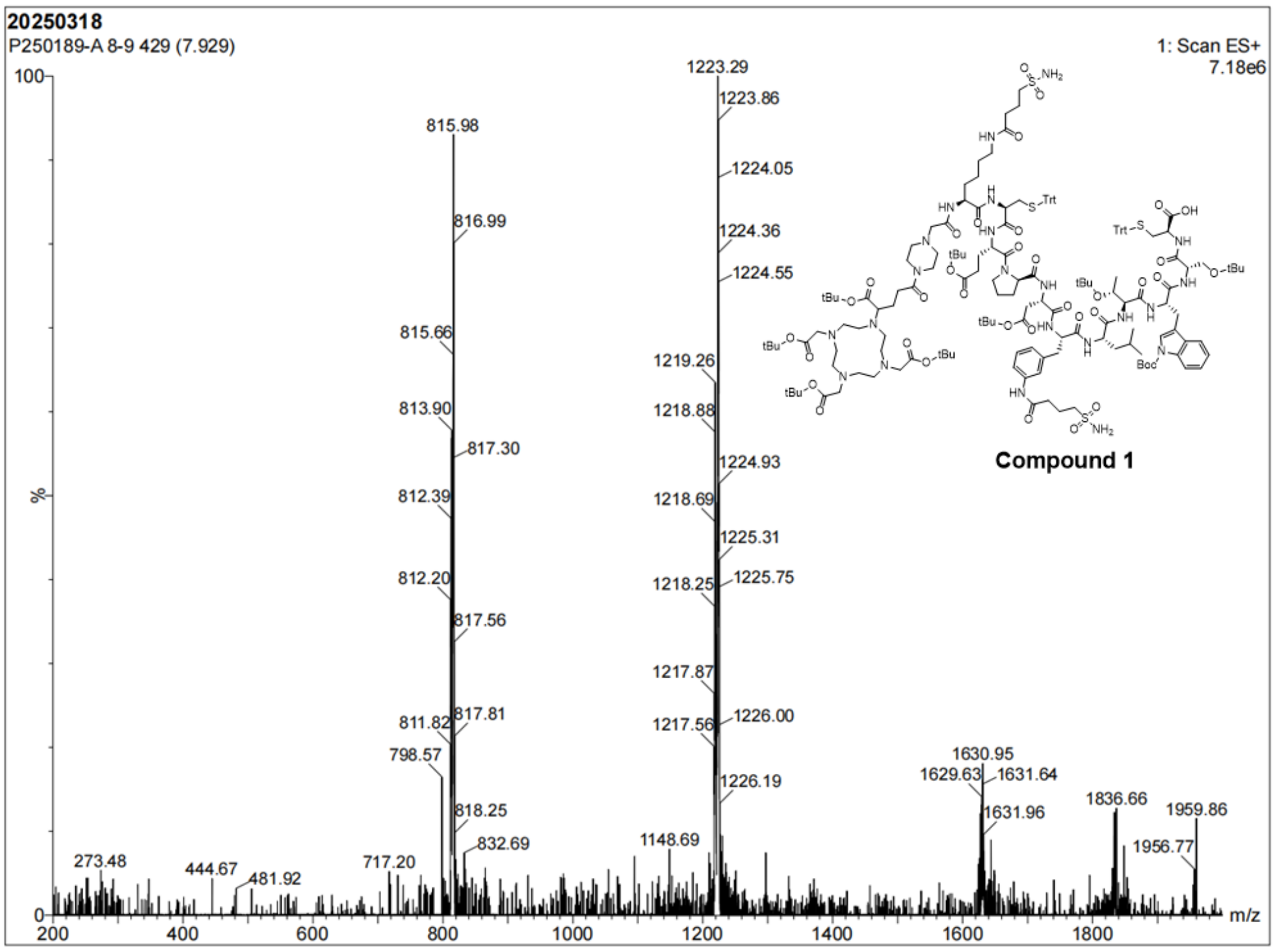

The High-resolution mass spectrometry for the reference compound **2**.

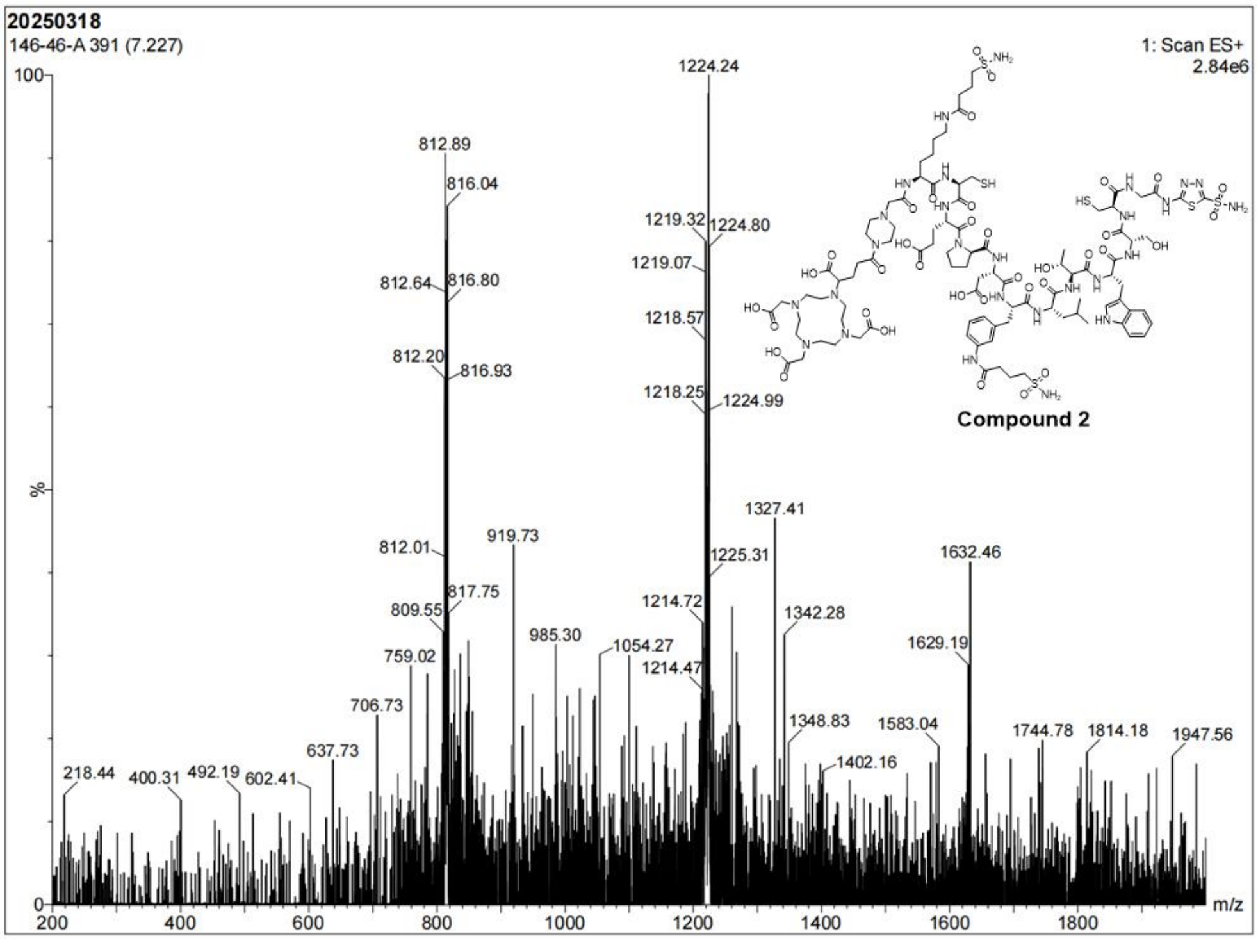

The High-resolution mass spectrometry for the reference ZH1.

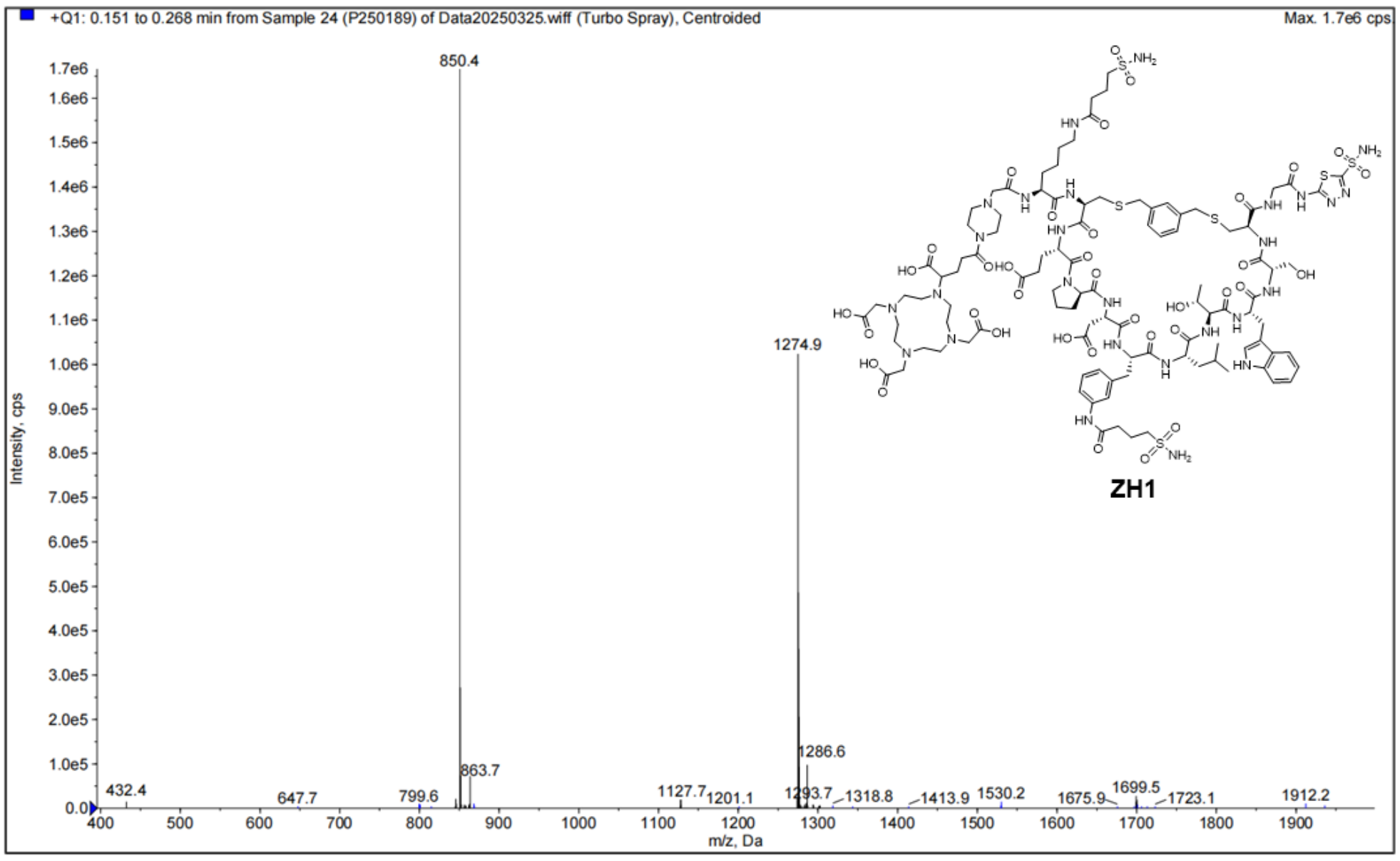

The High-resolution mass spectrometry for the reference compound **3**.

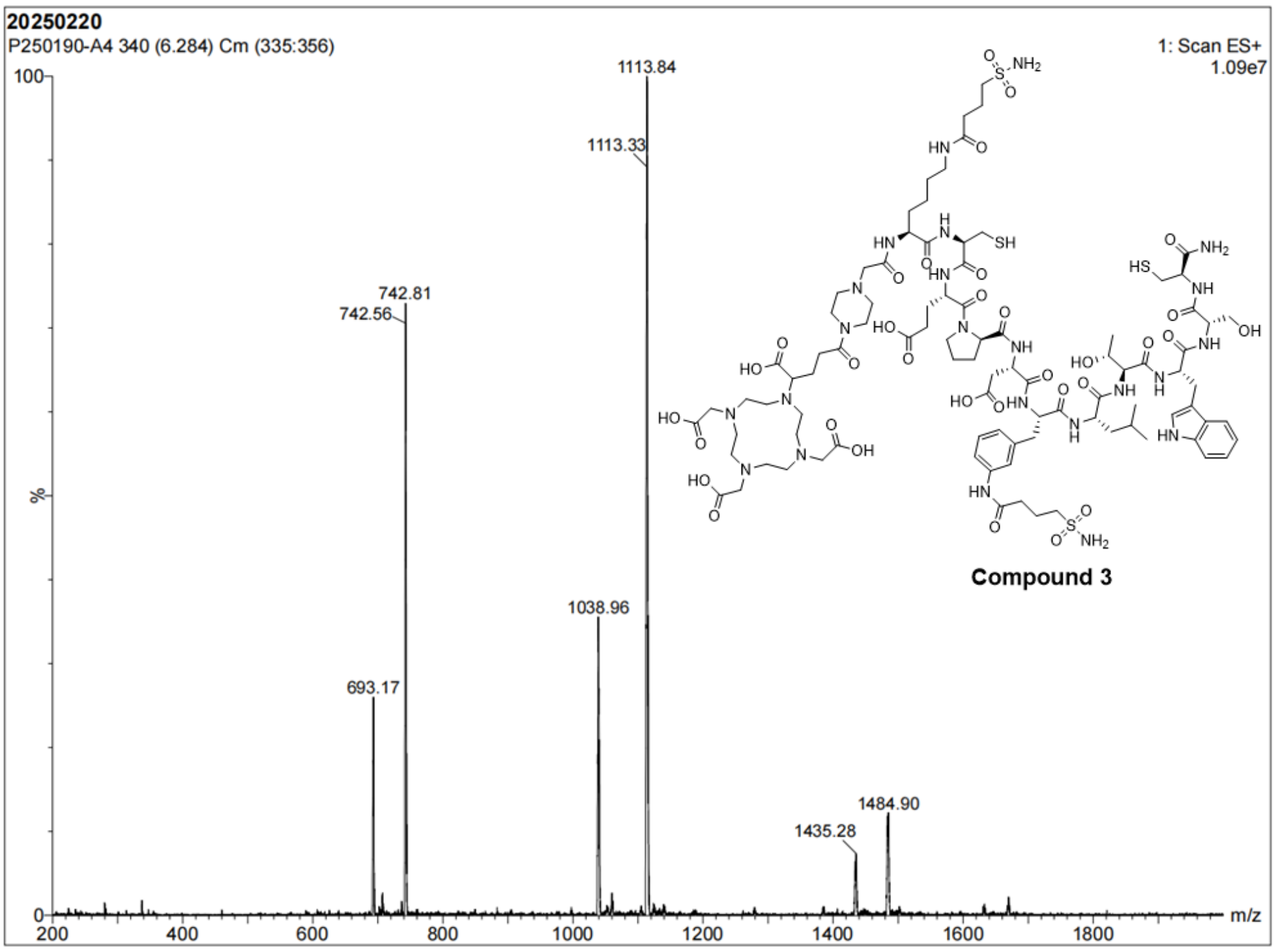

The High-resolution mass spectrometry for the reference ZH2.

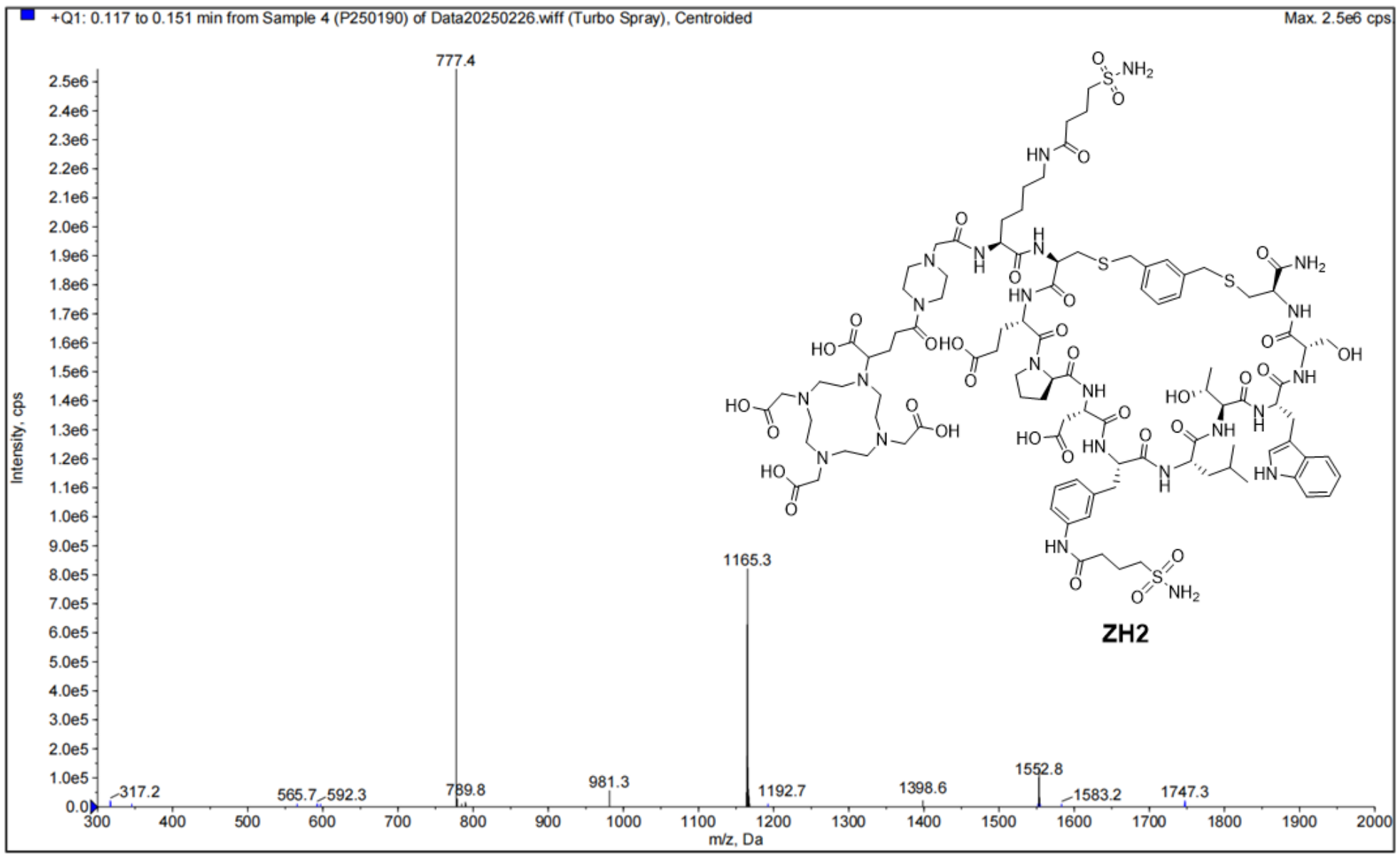

The High-resolution mass spectrometry for the reference compound **4**.

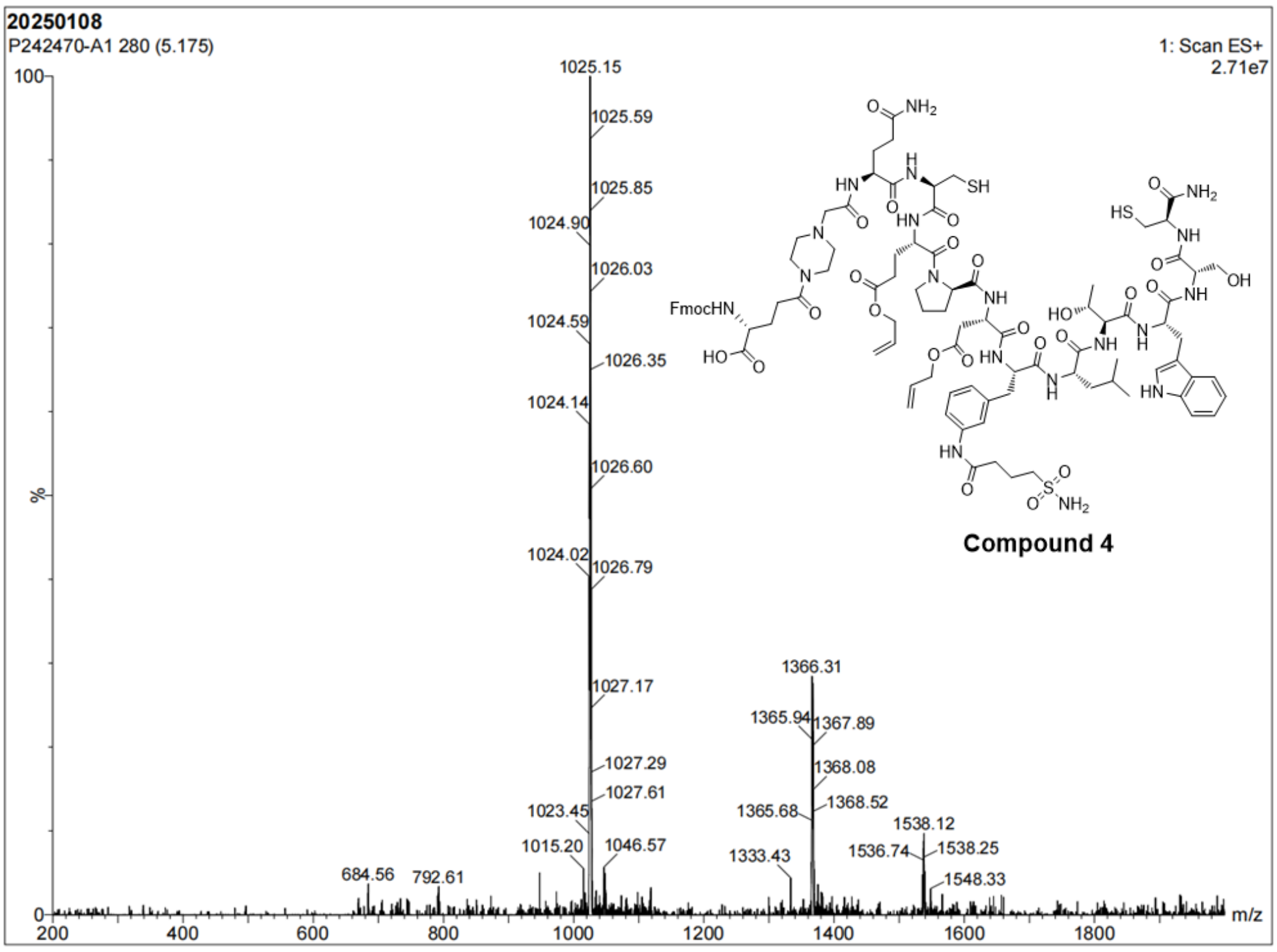

The High-resolution mass spectrometry for the reference compound **5**.

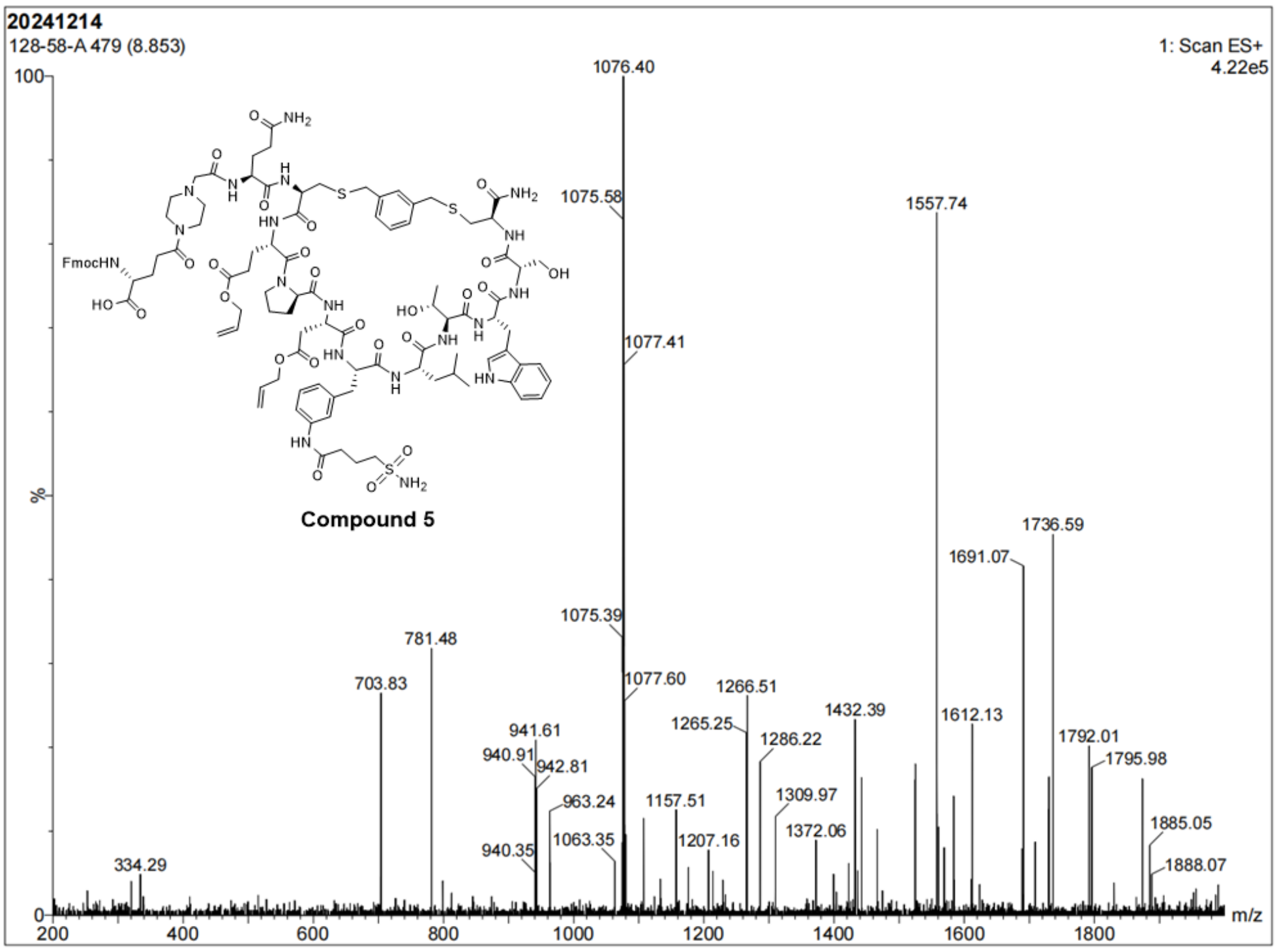

The High-resolution mass spectrometry for the reference compound **6**.

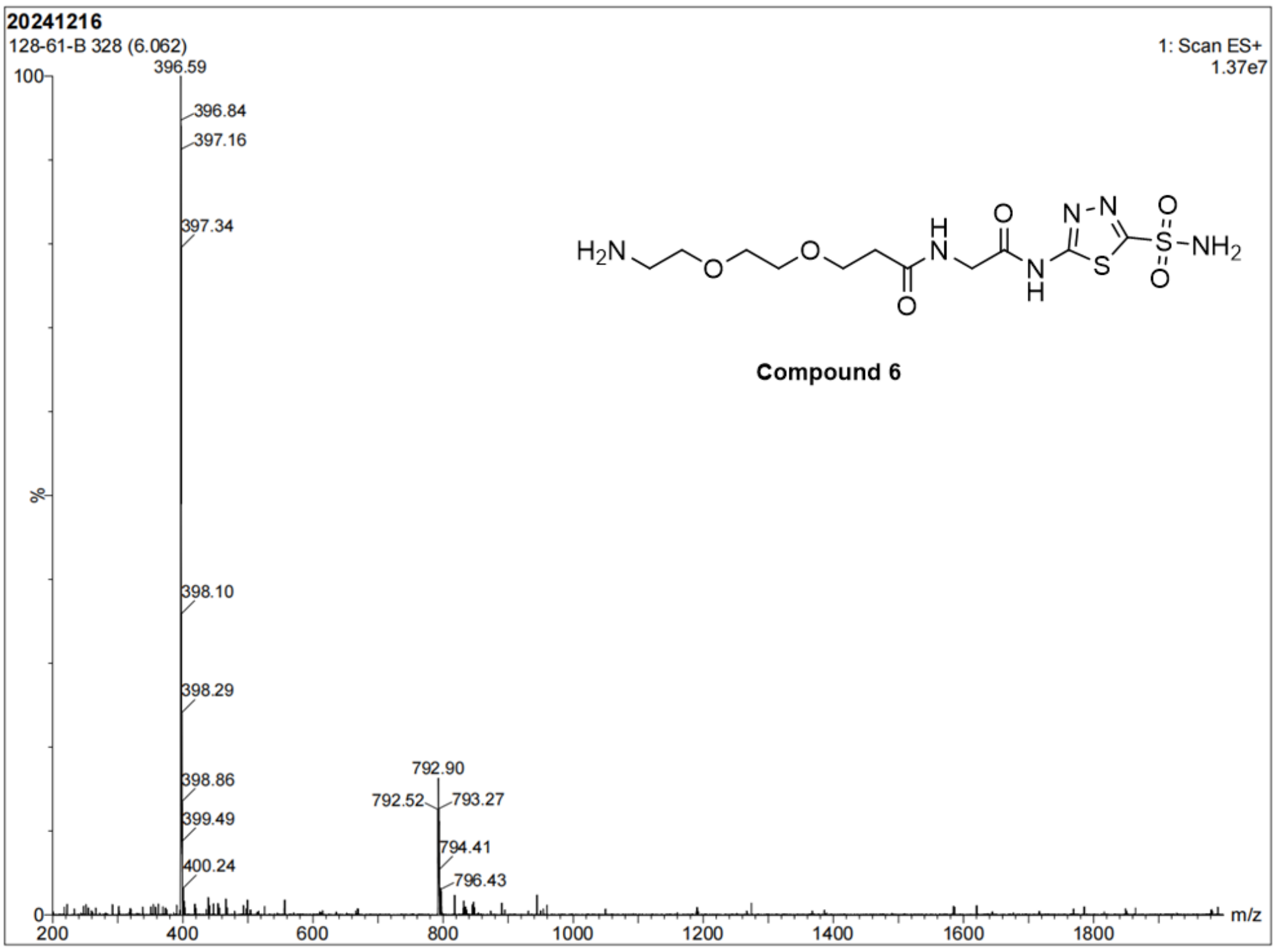

The High-resolution mass spectrometry for the reference compound **7**.

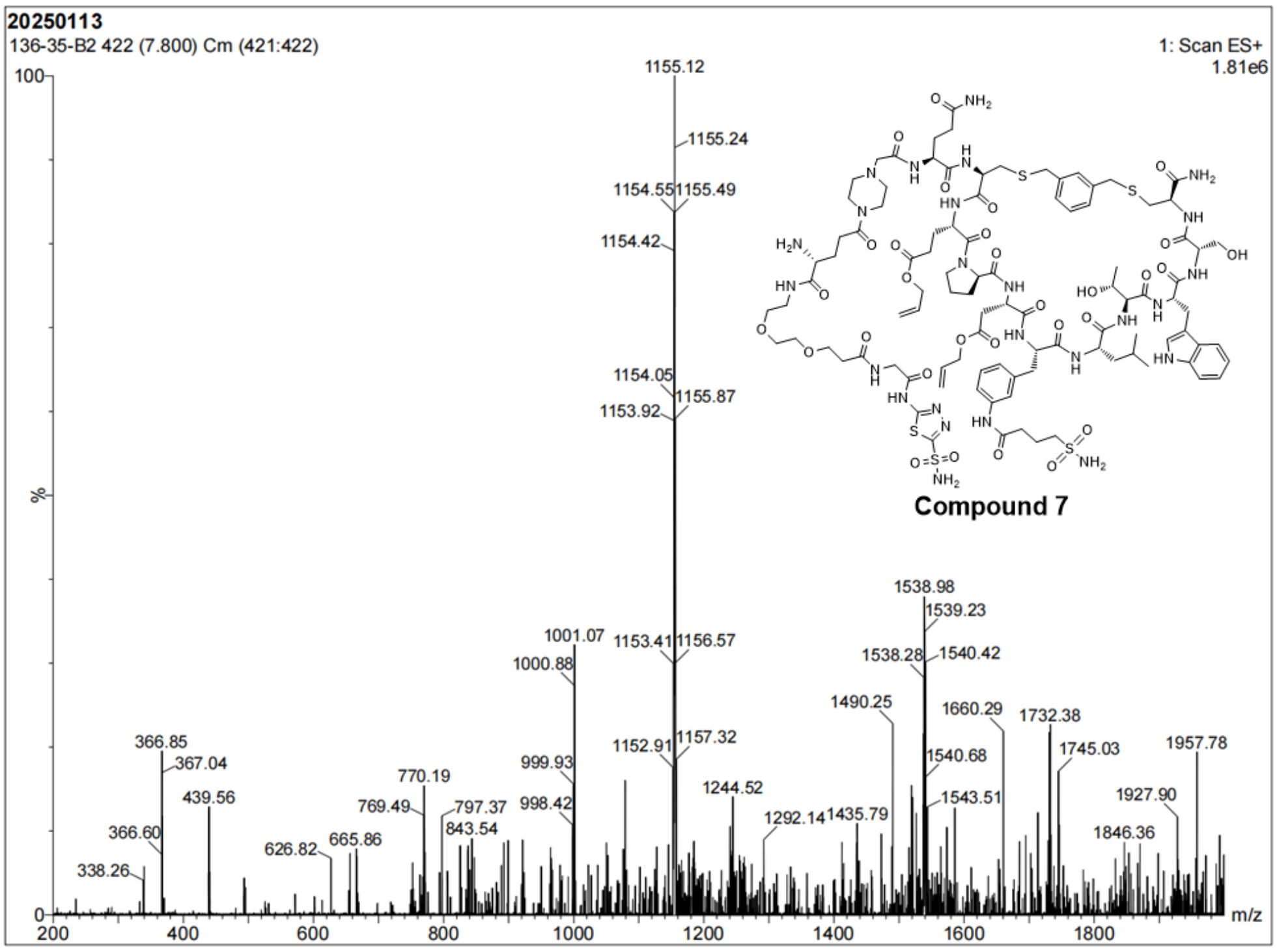

The High-resolution mass spectrometry for the reference ZH3.

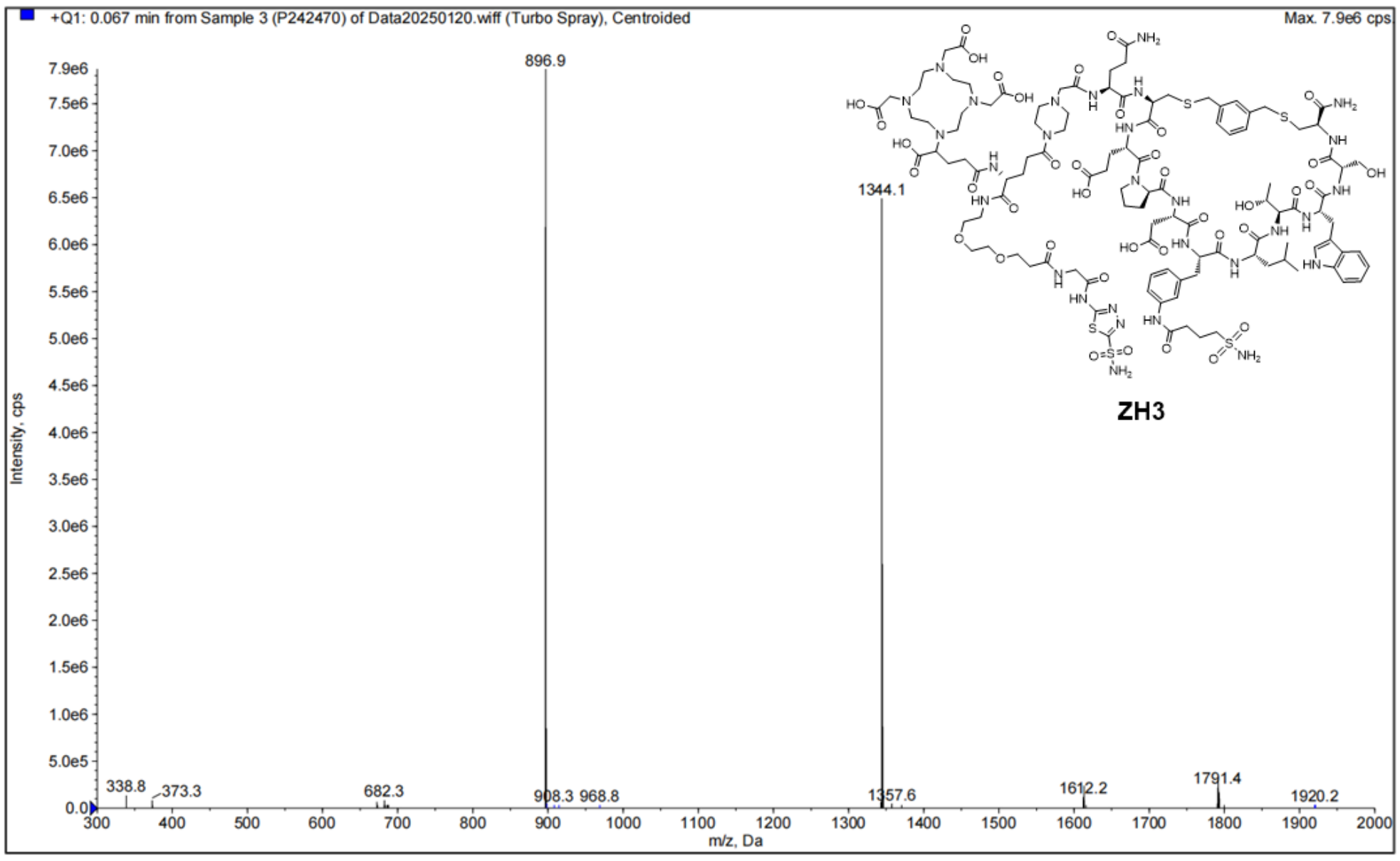

The High-resolution mass spectrometry for the reference compound **8**.

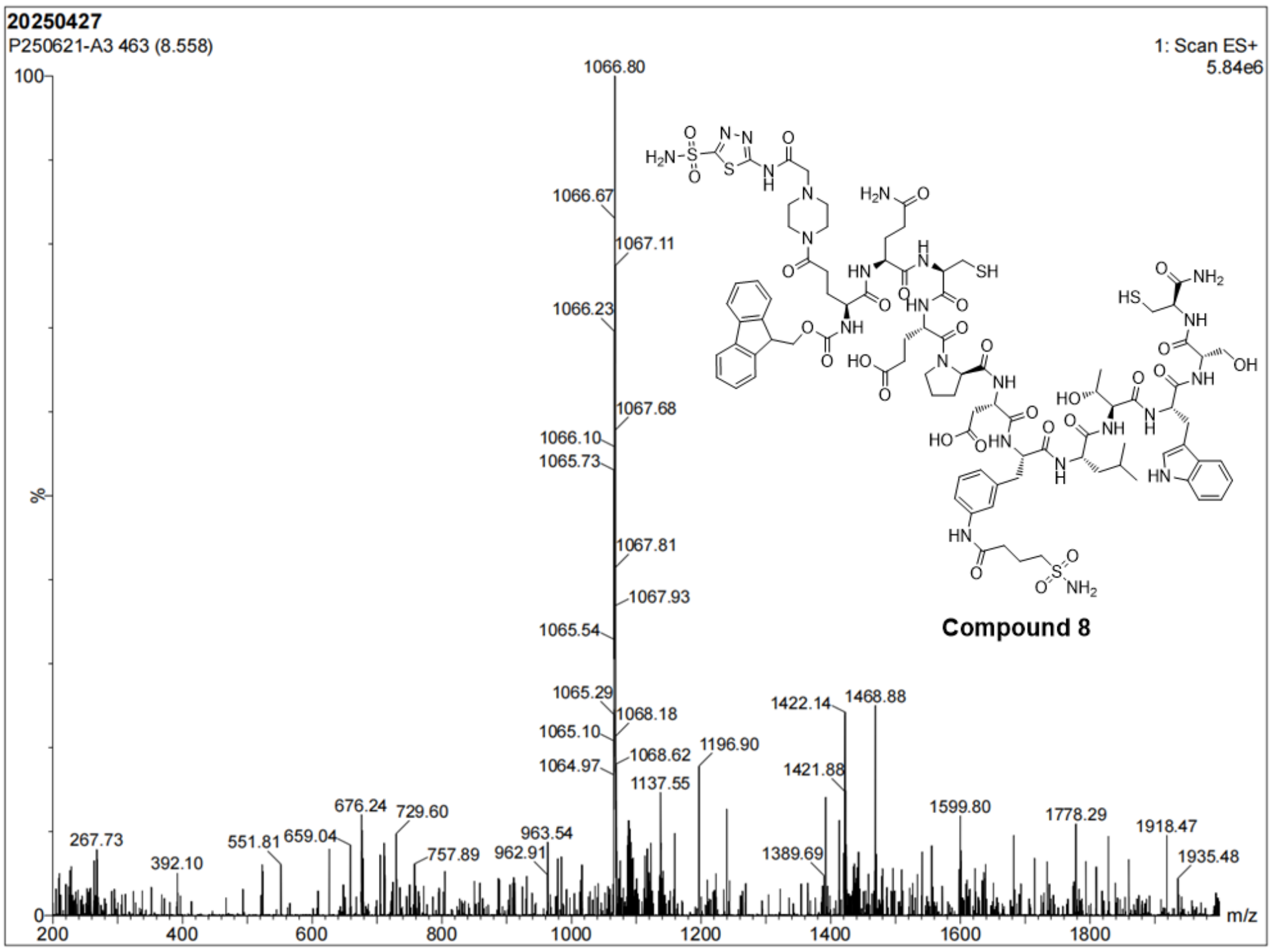

The High-resolution mass spectrometry for the reference compound **9**.

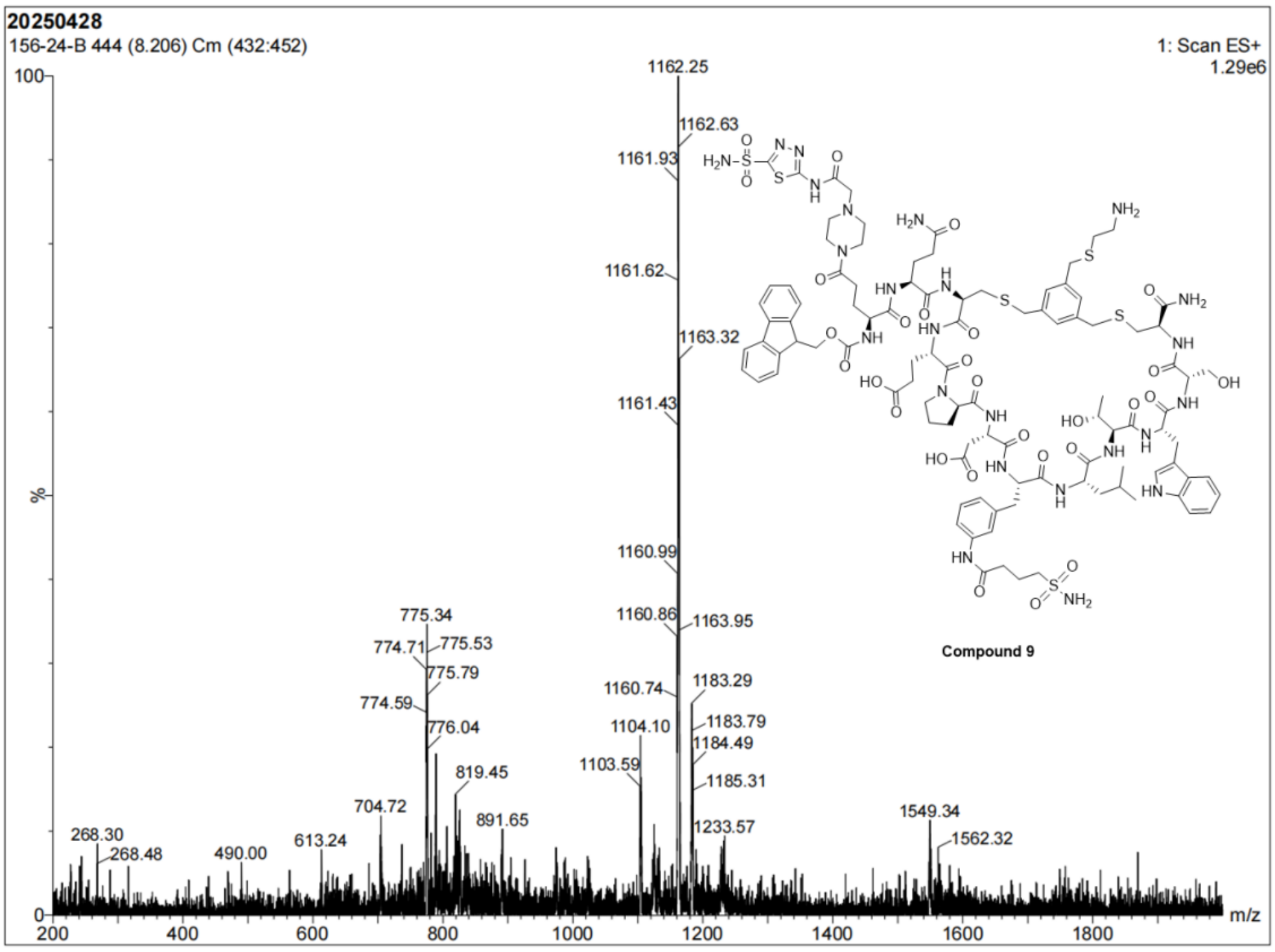

The High-resolution mass spectrometry for the reference ZH4.

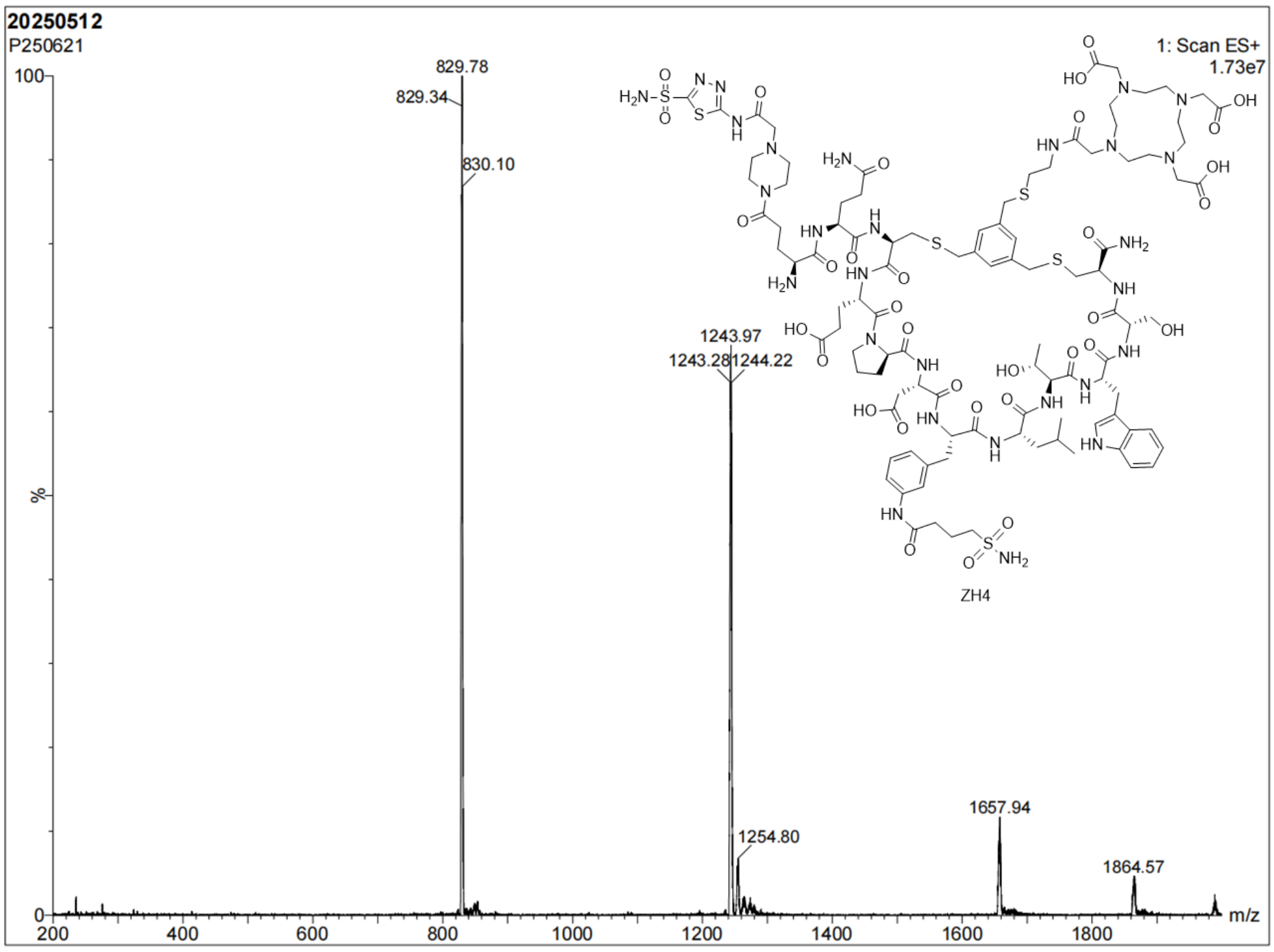

The High-resolution mass spectrometry for the reference compound **10**.

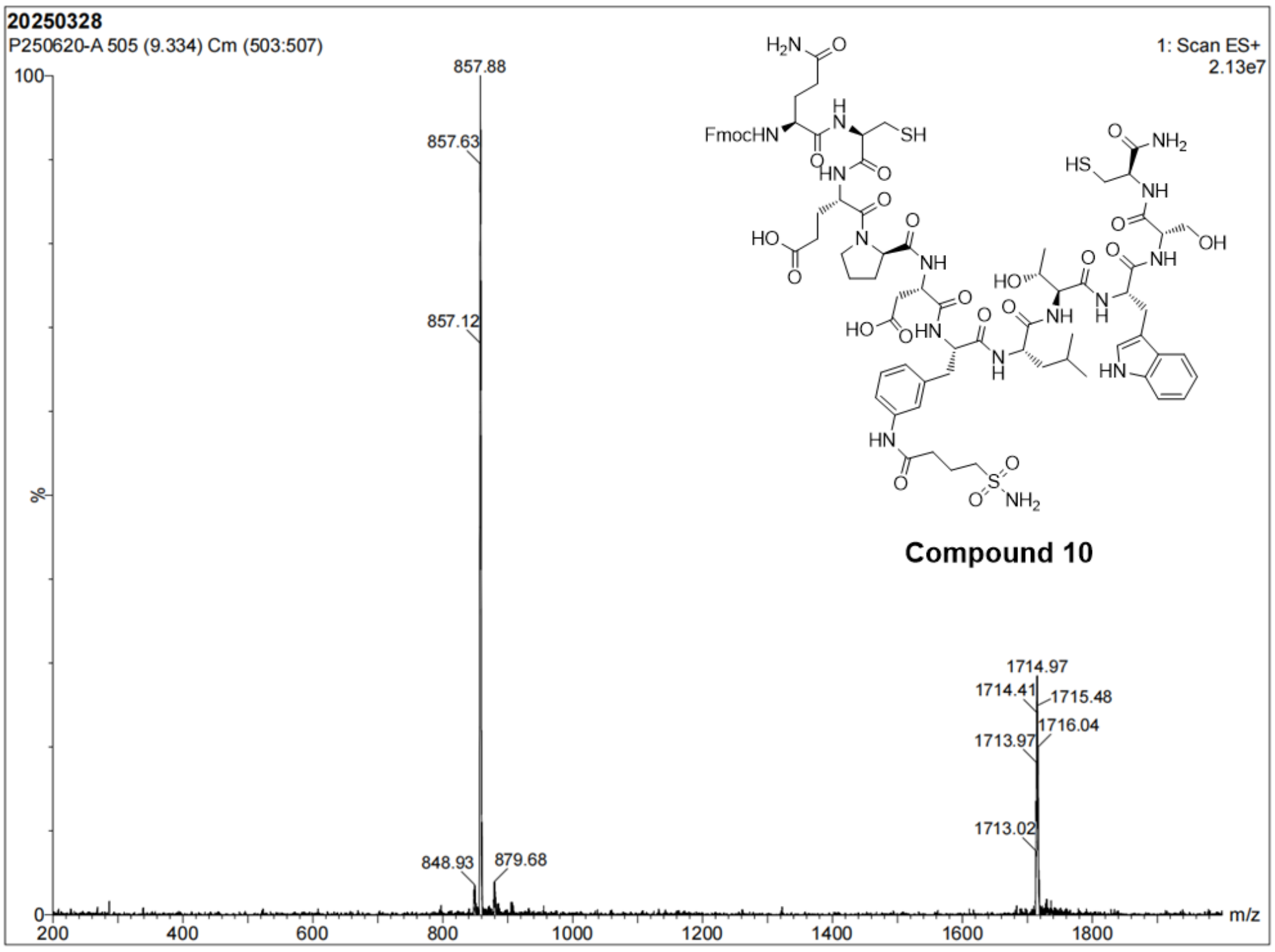

The High-resolution mass spectrometry for the reference compound **11**.

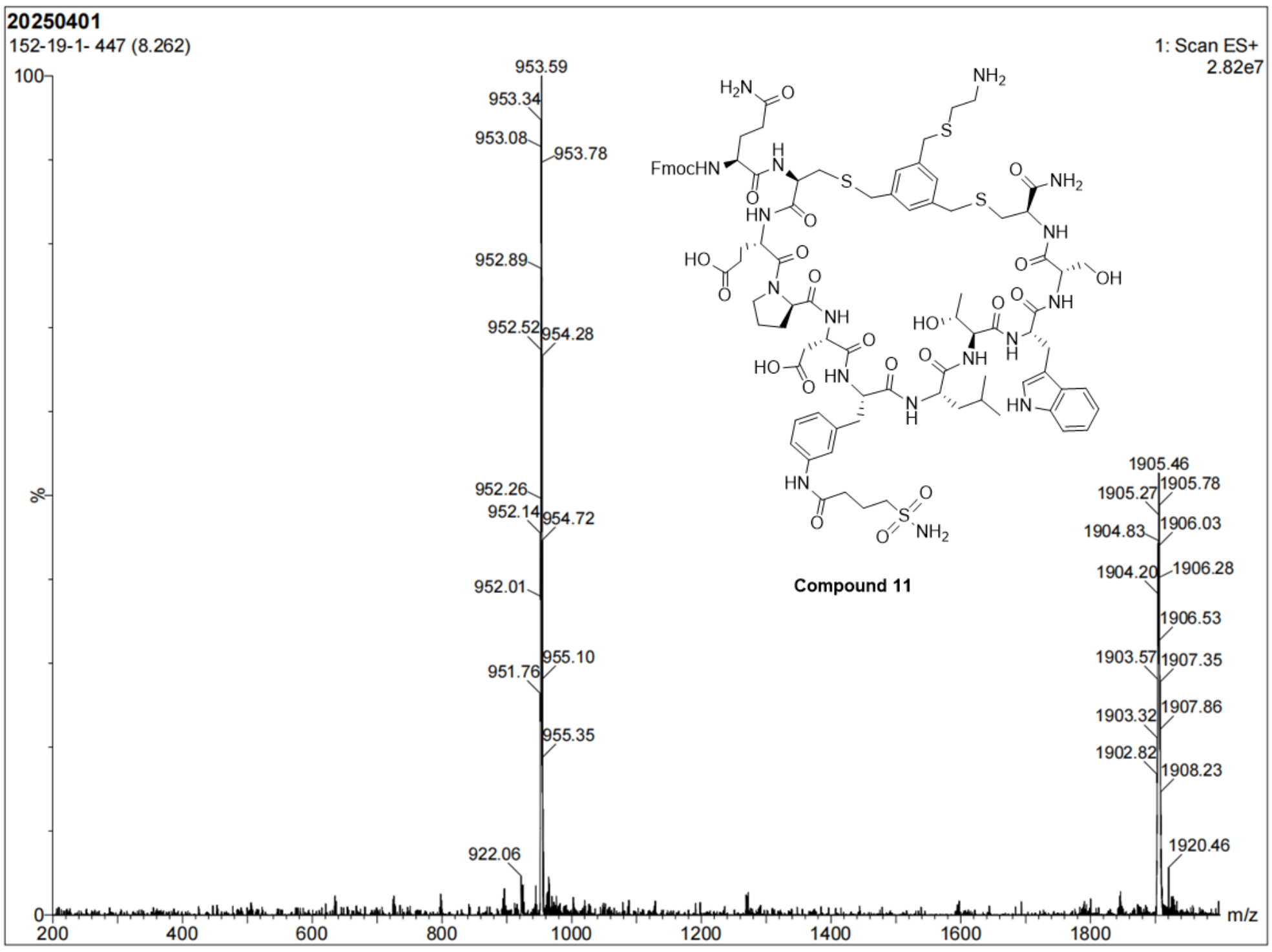

The High-resolution mass spectrometry for the reference compound **12**.

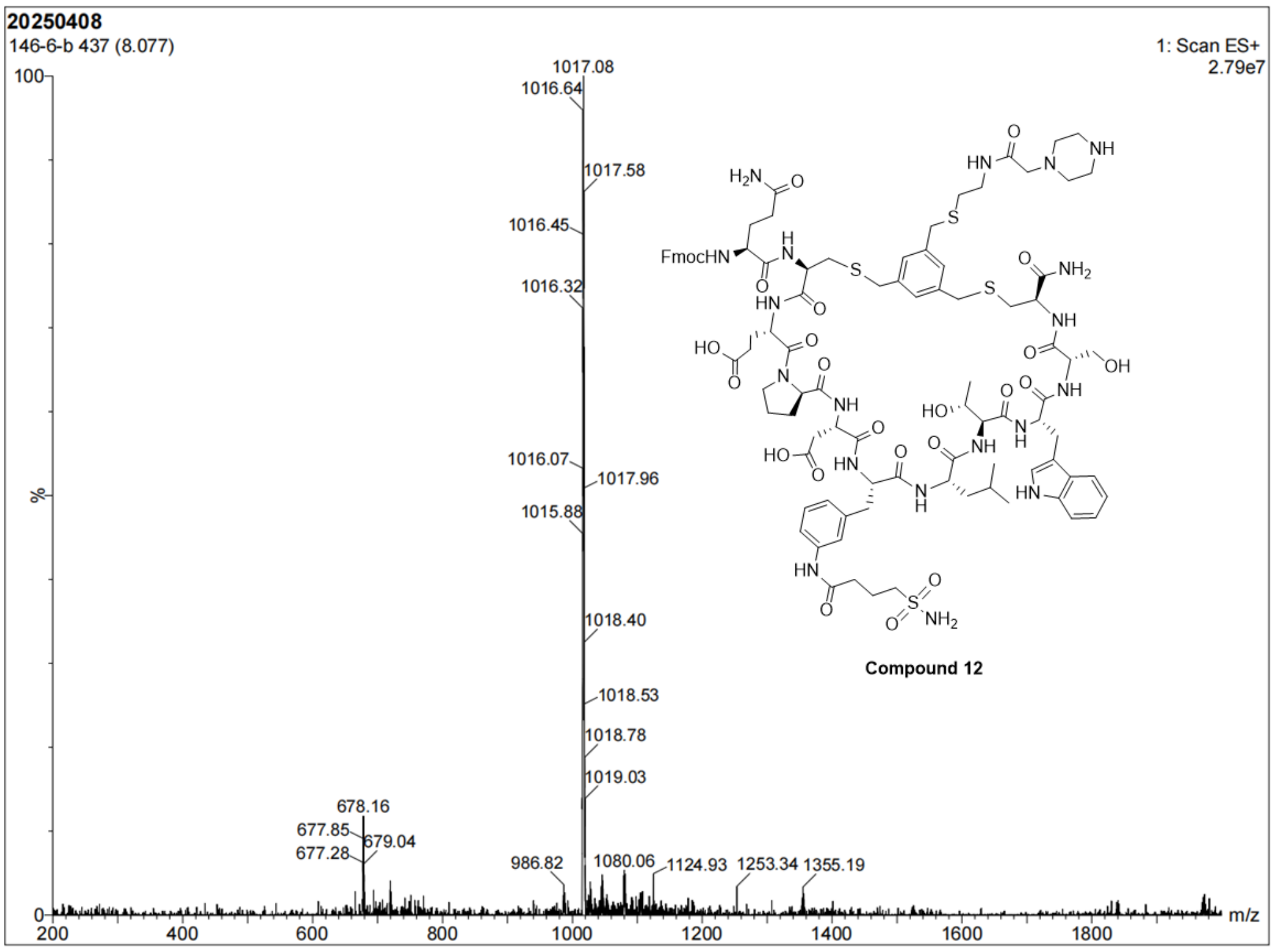

The High-resolution mass spectrometry for the reference ZH5.

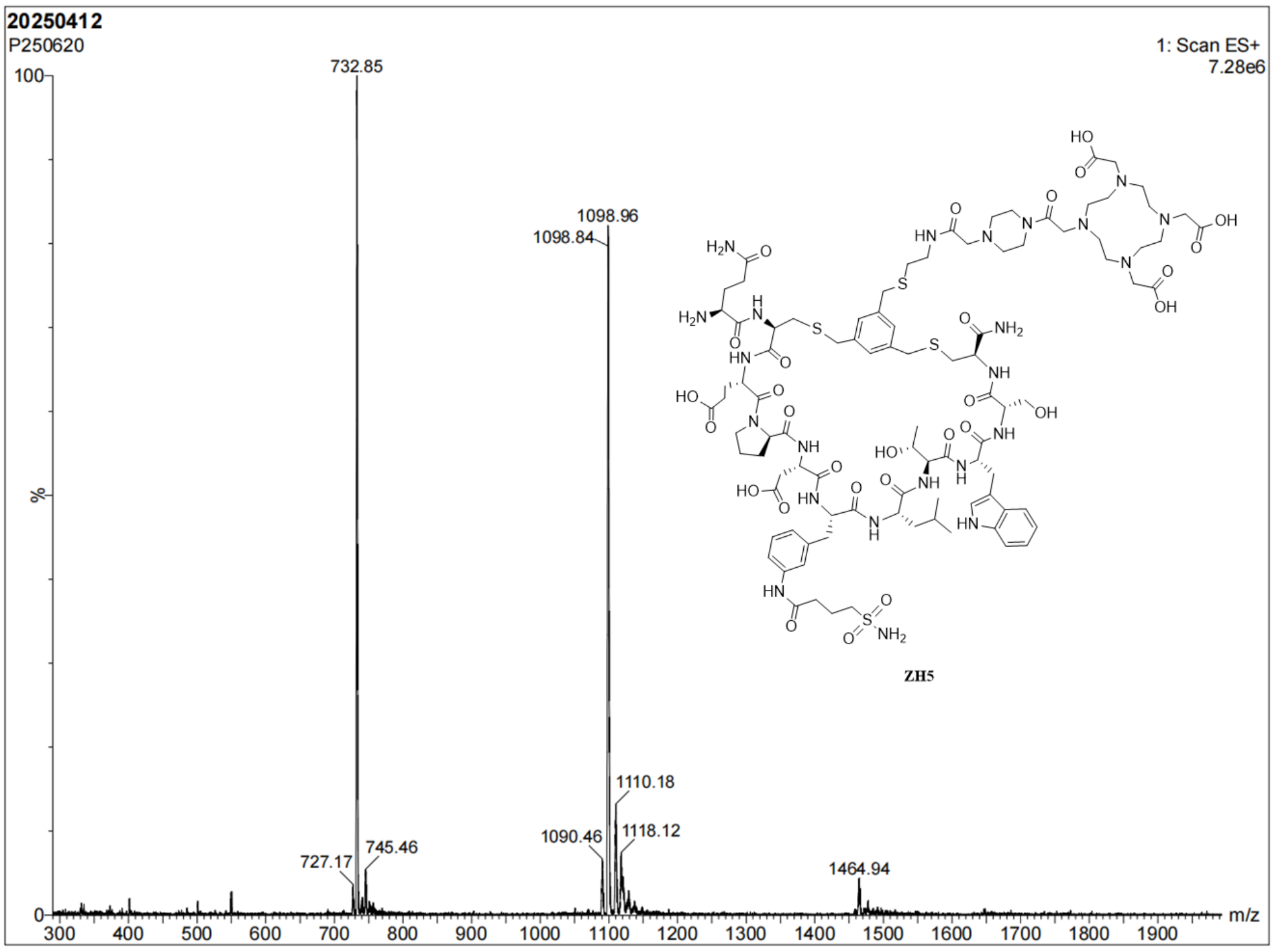

The HPLC profiles of ZH1.

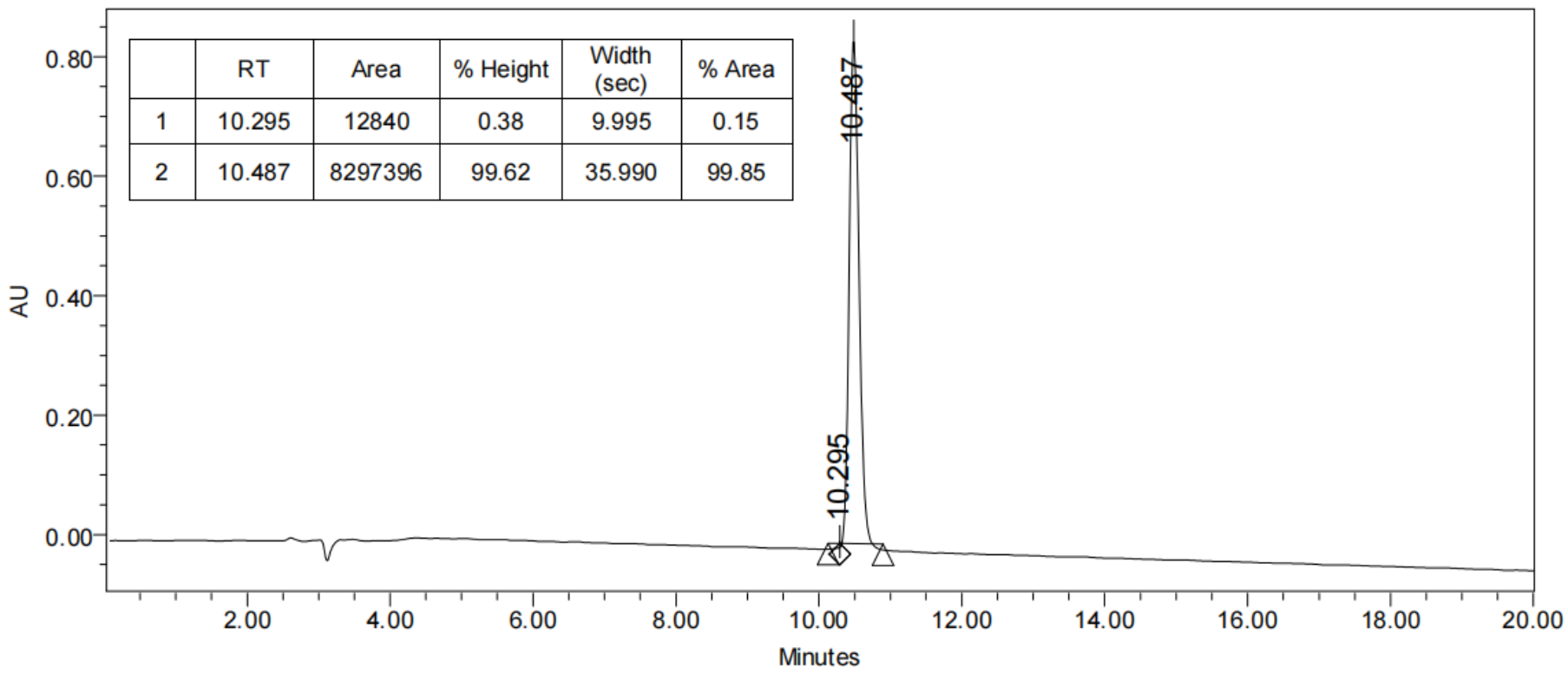

	RT	Area	% Height	Width (sec)	% Area
1	10.295	12840	0.38	9.995	0.15
2	10.487	8297396	99.62	35.990	99.85

The HPLC profiles of ZH2.

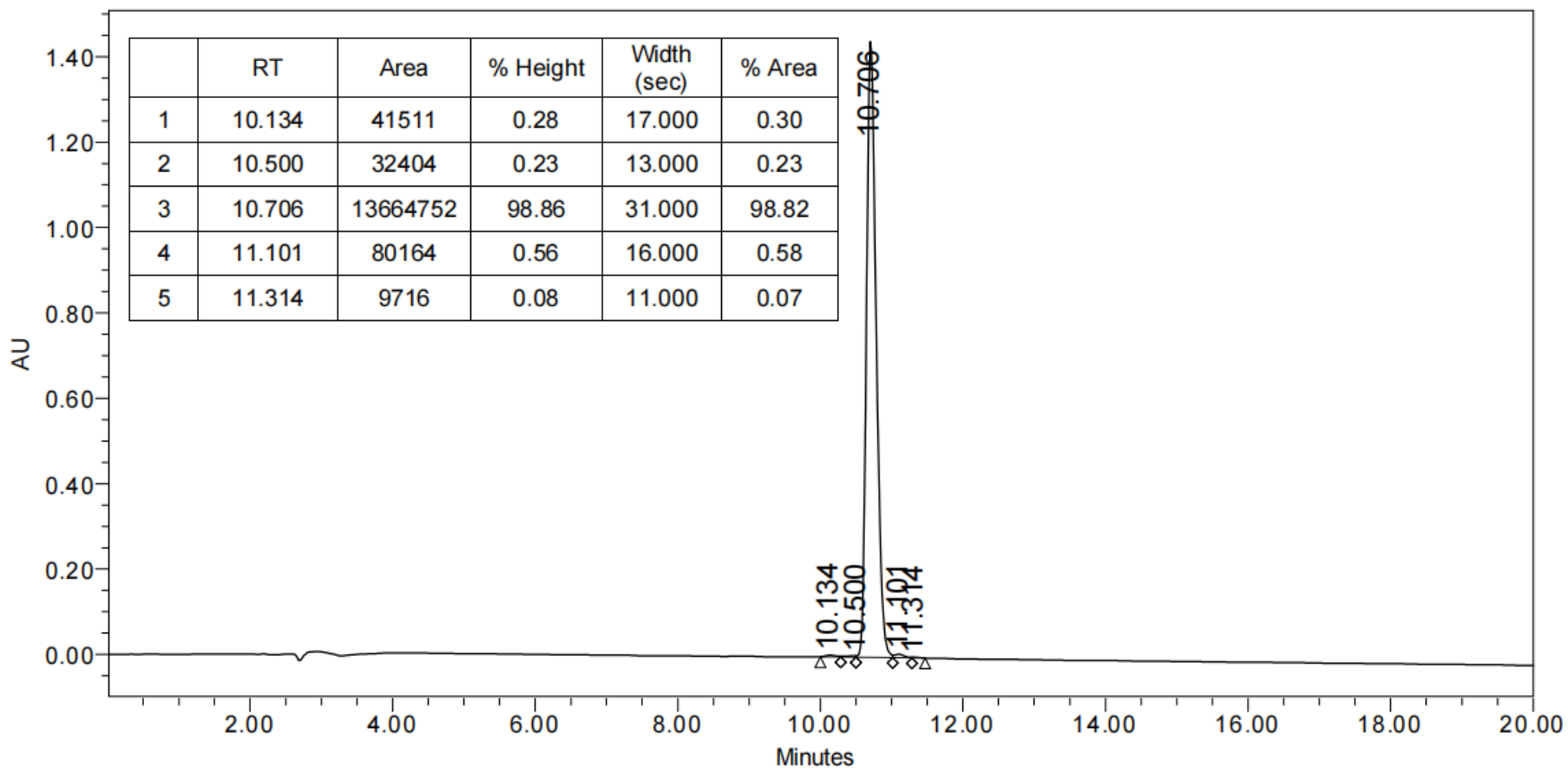

	RT	Area	% Height	Width (sec)	% Area
1	10.134	41511	0.28	17.000	0.30
2	10.500	32404	0.23	13.000	0.23
3	10.706	13664752	98.86	31.000	98.82
4	11.101	80164	0.56	16.000	0.58
5	11.314	9716	0.08	11.000	0.07

The HPLC profiles of ZH3.

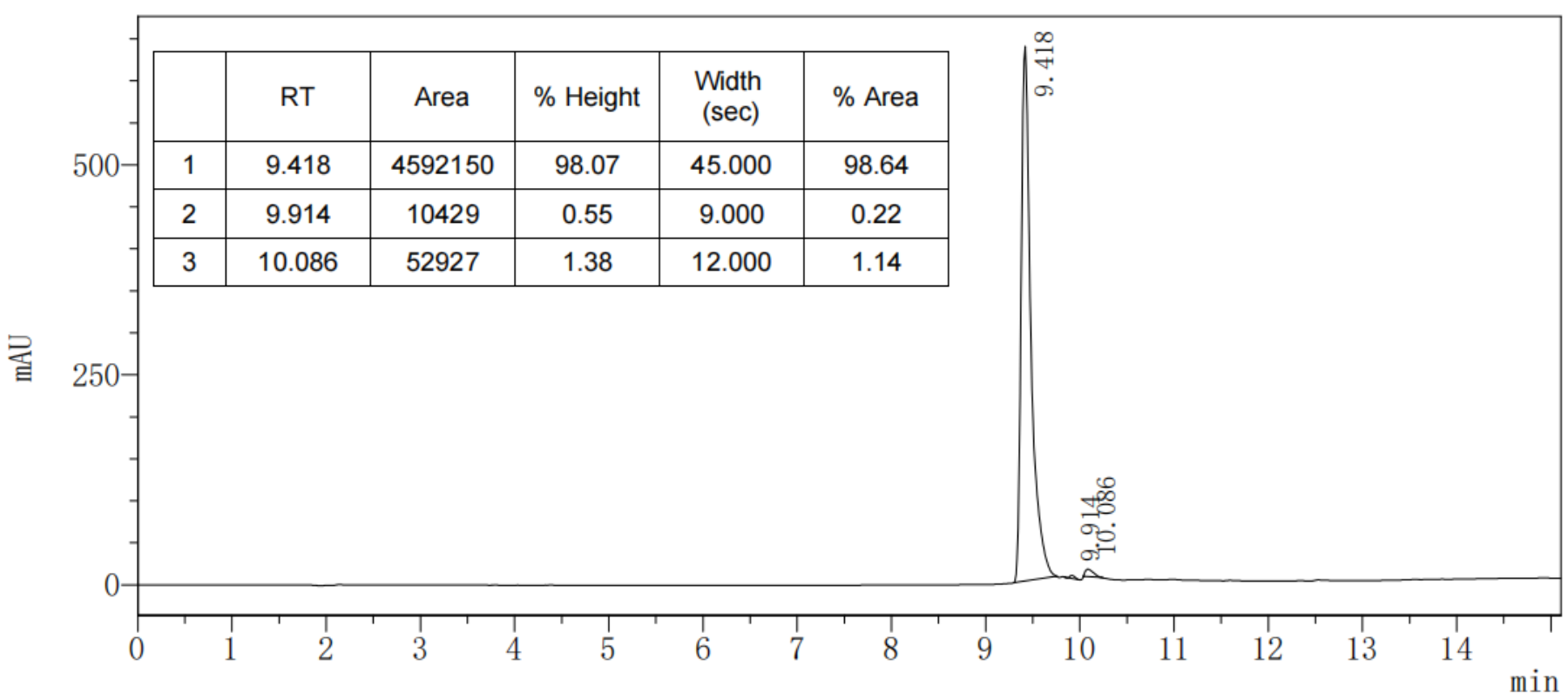

	RT	Area	% Height	Width (sec)	% Area
1	9.418	4592150	98.07	45.000	98.64
2	9.914	10429	0.55	9.000	0.22
3	10.086	52927	1.38	12.000	1.14

The HPLC profiles of ZH4.

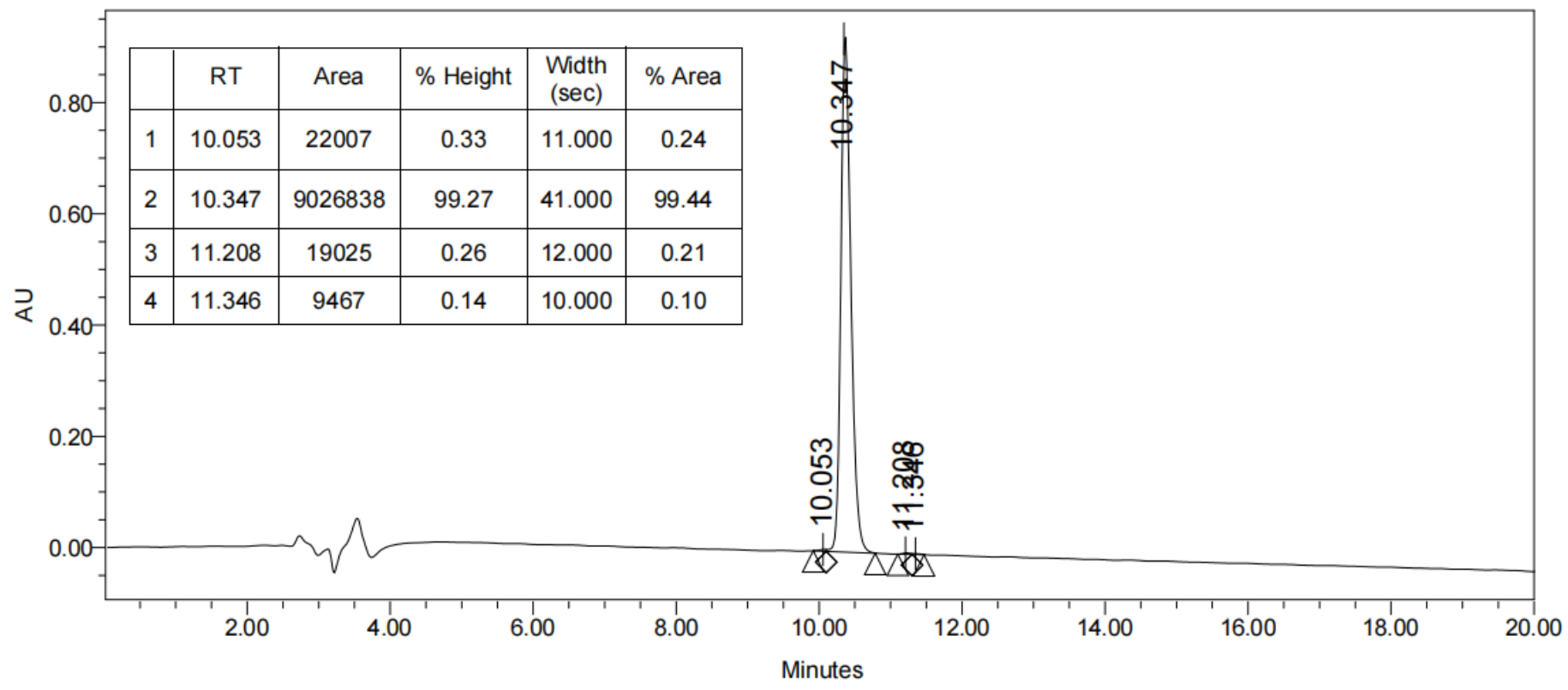

	RT	Area	% Height	Width (sec)	% Area
1	10.053	22007	0.33	11.000	0.24
2	10.347	9026838	99.27	41.000	99.44
3	11.208	19025	0.26	12.000	0.21
4	11.346	9467	0.14	10.000	0.10

The HPLC profiles of ZH5.

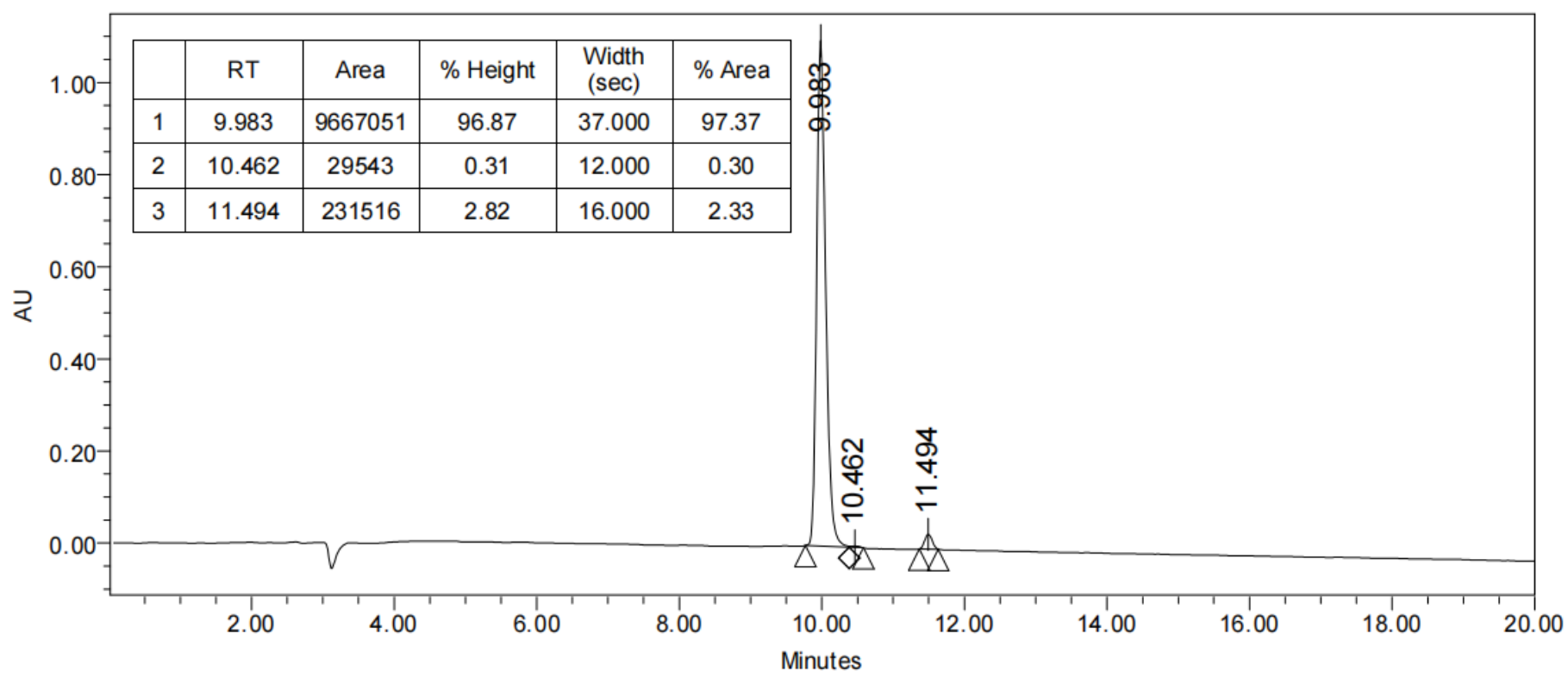

	RT	Area	% Height	Width (sec)	% Area
1	9.983	9667051	96.87	37.000	97.37
2	10.462	29543	0.31	12.000	0.30
3	11.494	231516	2.82	16.000	2.33

2. Supplementary Figures

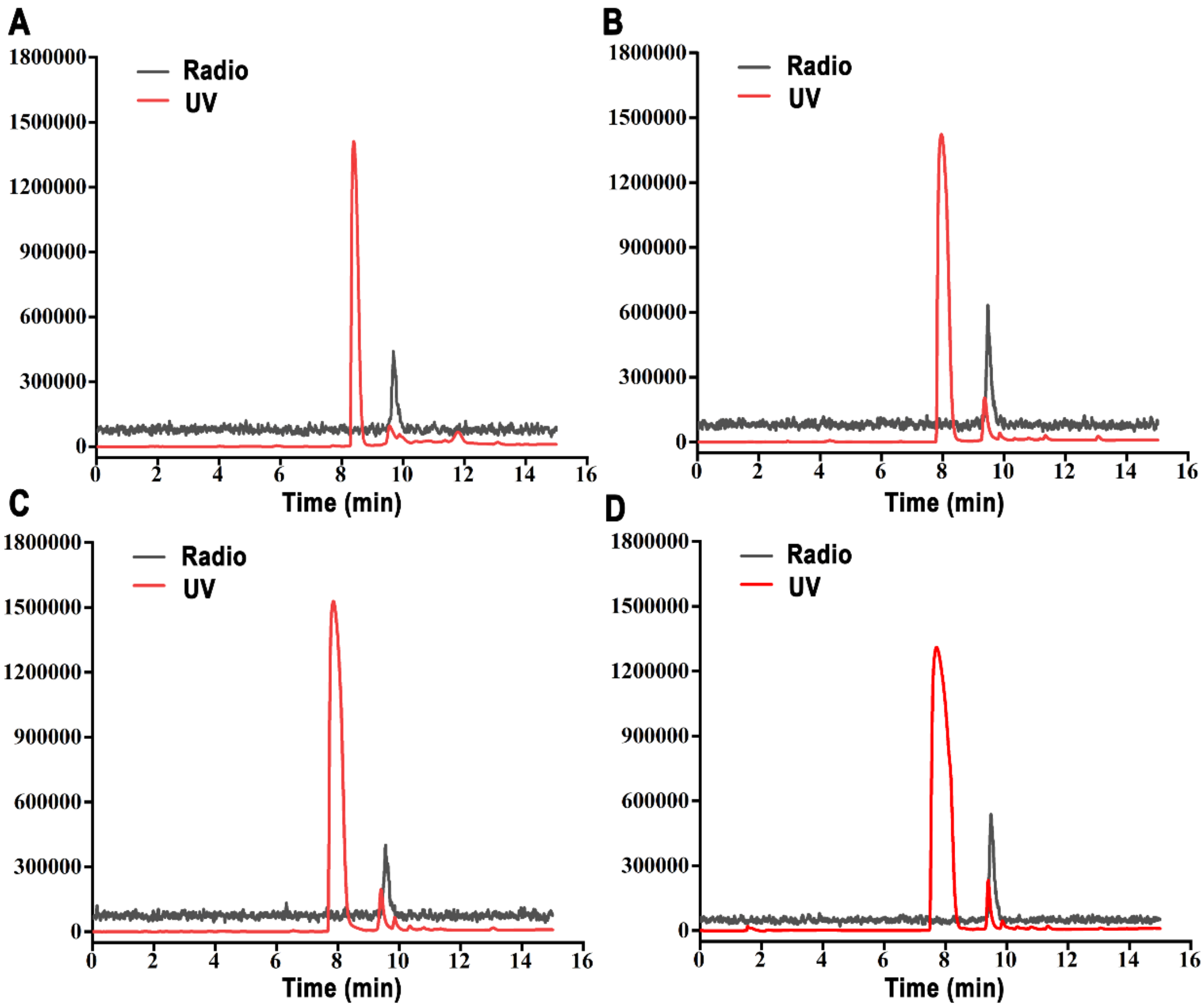

Figure S1. The quality control of the radiolabeled ligands. ^{177}Lu-ZH1 (**A**), ^{177}Lu-ZH2 (**B**), ^{177}Lu-ZH3 (**C**) and ^{177}Lu-DPI-4452 (**D**).

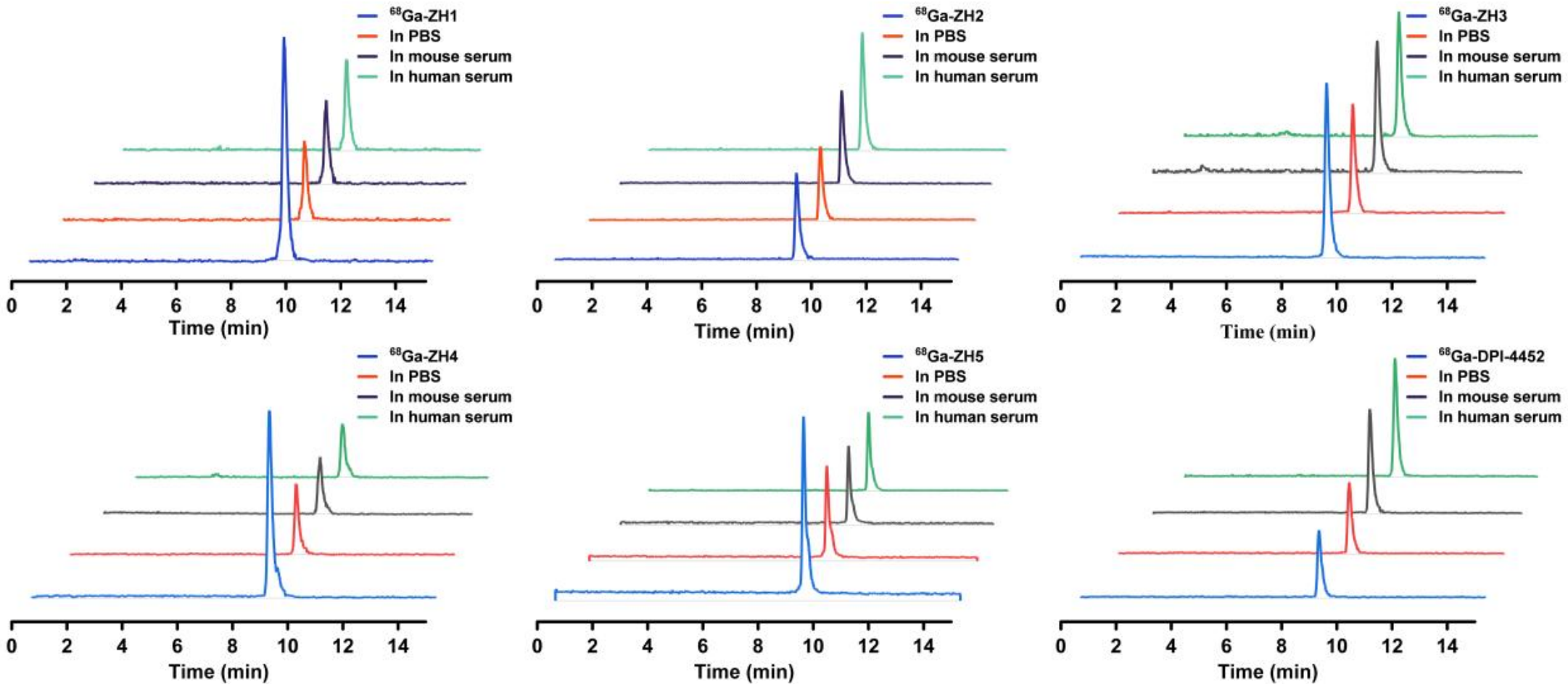

Figure S2. In vitro stability of ^{68}Ga-ZH1–5 and ^{68}Ga-DPI-4452 in PBS, mouse serum and human serum for 2 h.

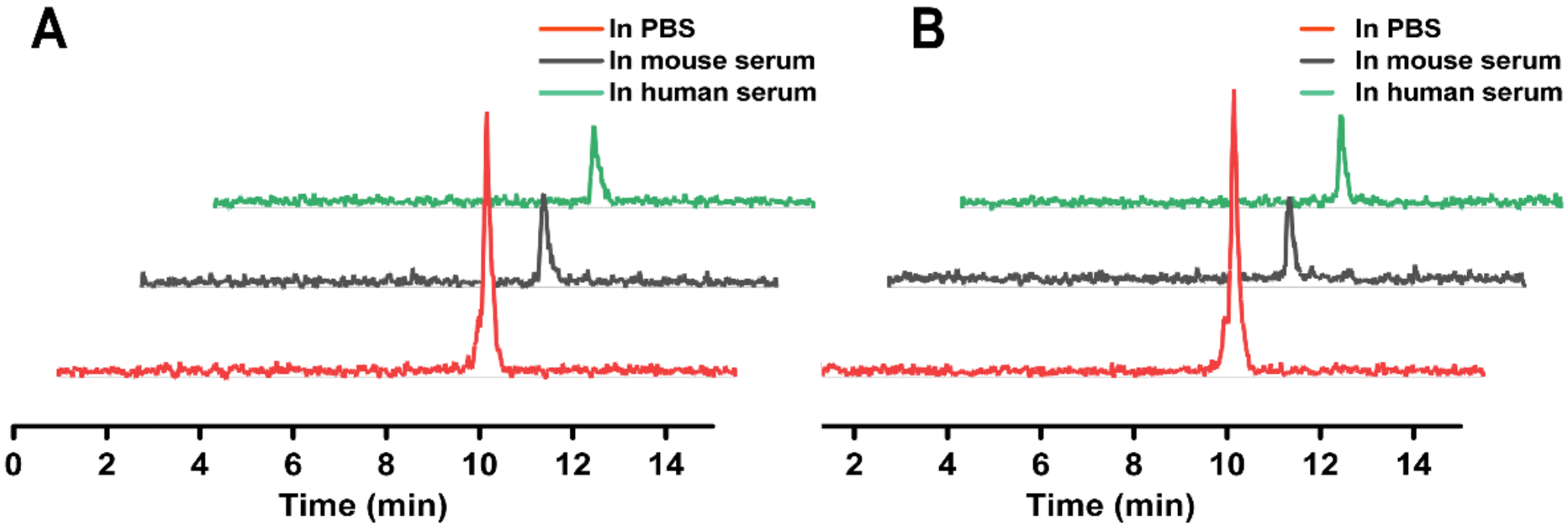

Figure S3. In vitro stability of ^{177}Lu-ZH2 (A) and ^{177}Lu-DPI-4452 (B) in PBS, mouse serum and human serum for 24 h.

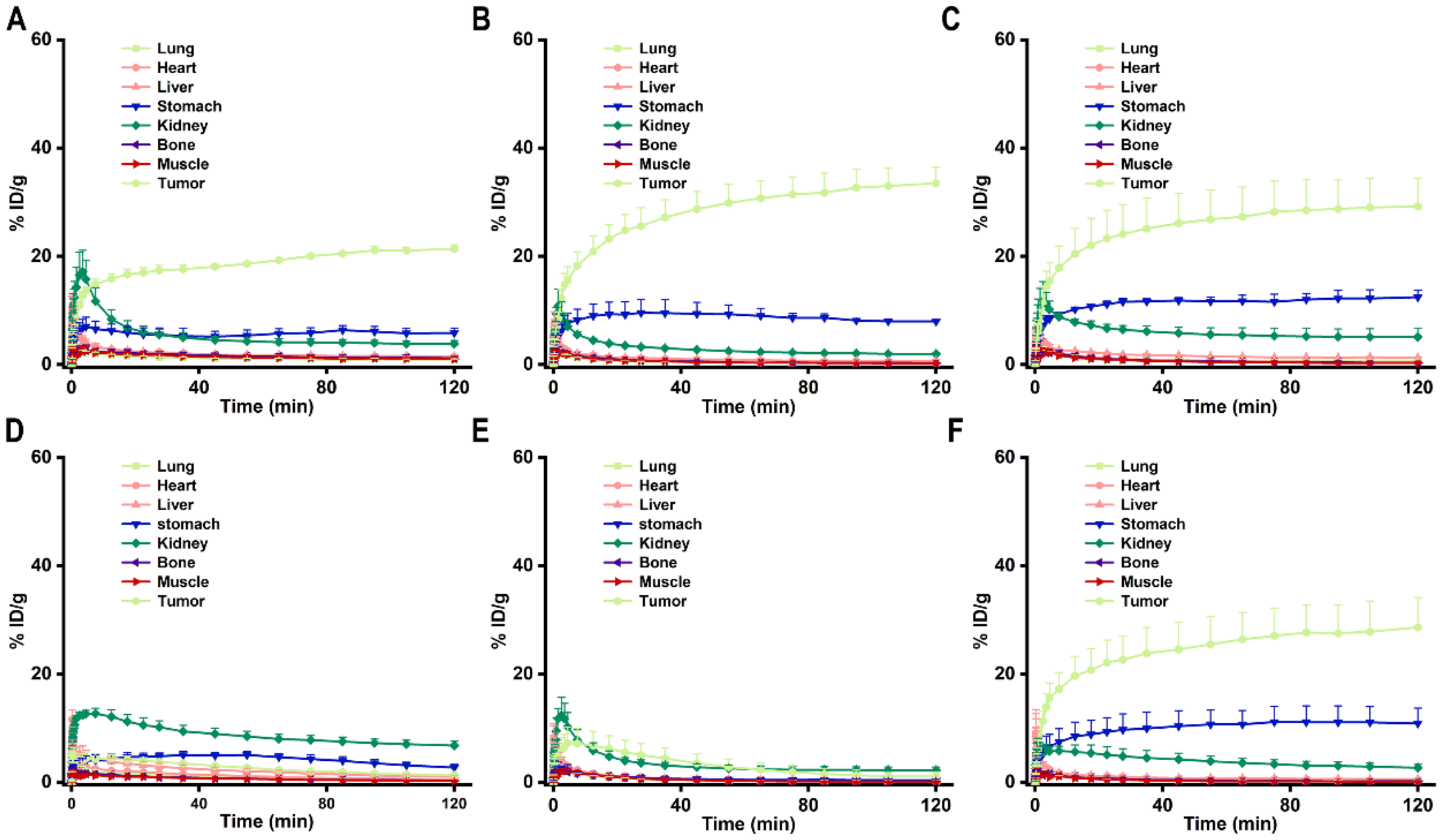

Figure S4. Time-activity curves of ^{68}Ga-ZH1–5 (A–E) and ^{68}Ga-DPI-4452 (F) in selected organs and tumor for 2 h. (*n* = 3–4, mean ± SD).

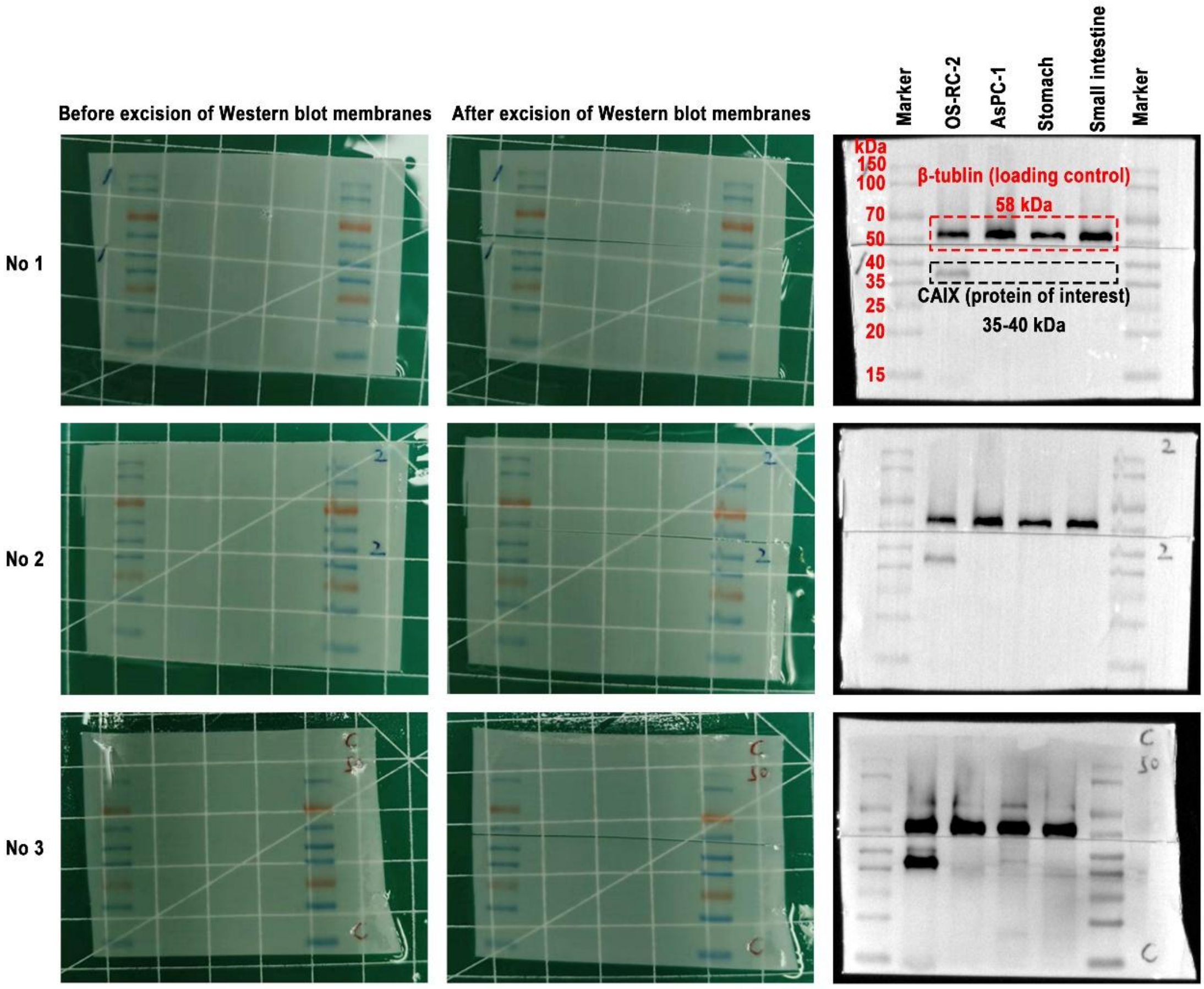

Figure S5. The original gel blots of WB analysis, *n* = 3.

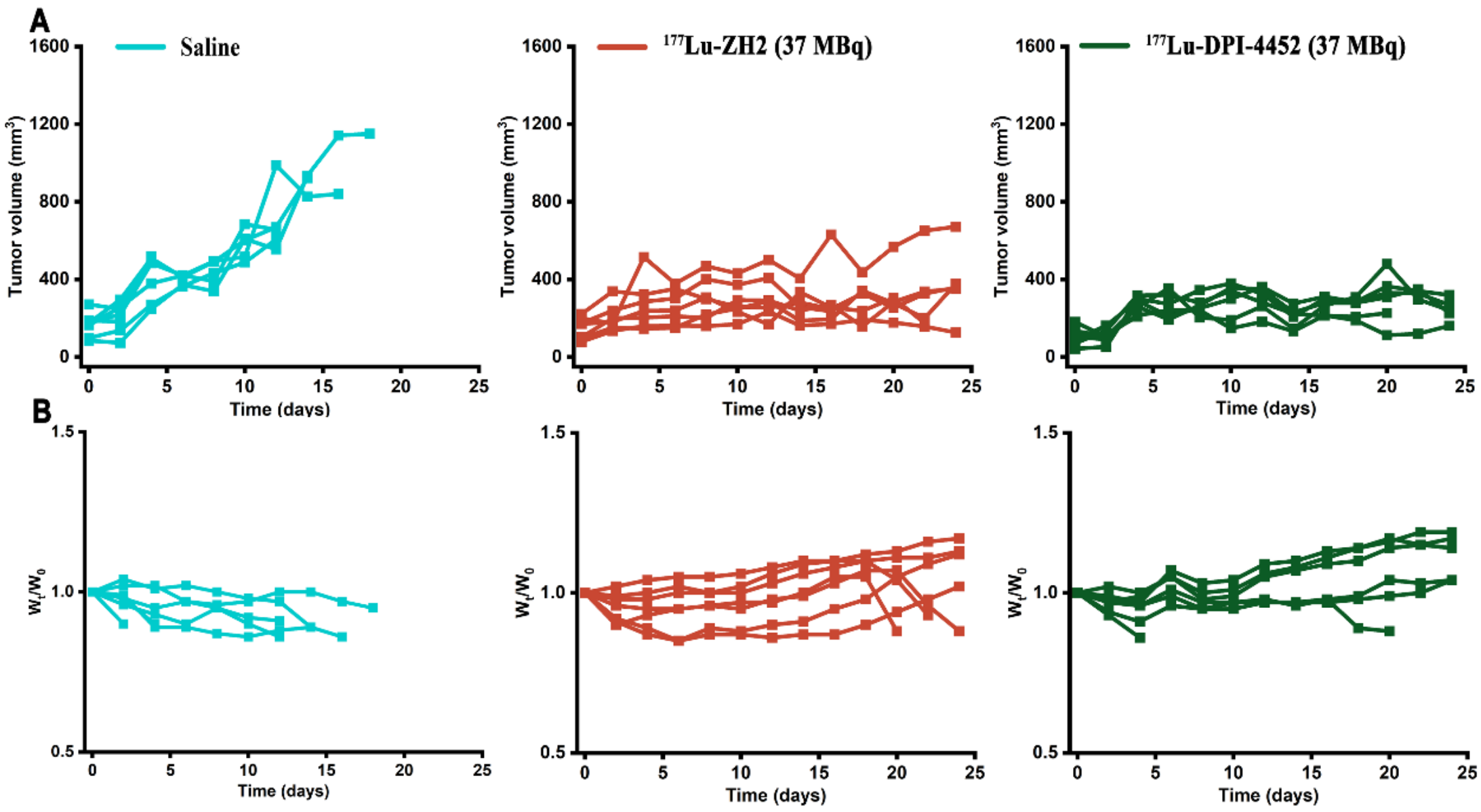

Figure S6. Therapeutic efficacy of CAIX-targeted ^{177}Lu-radiolabeling ligands. Tumor growth curve (**A**) and body weight (Wt/W0) changes (**B**) from day 0 to day 24.

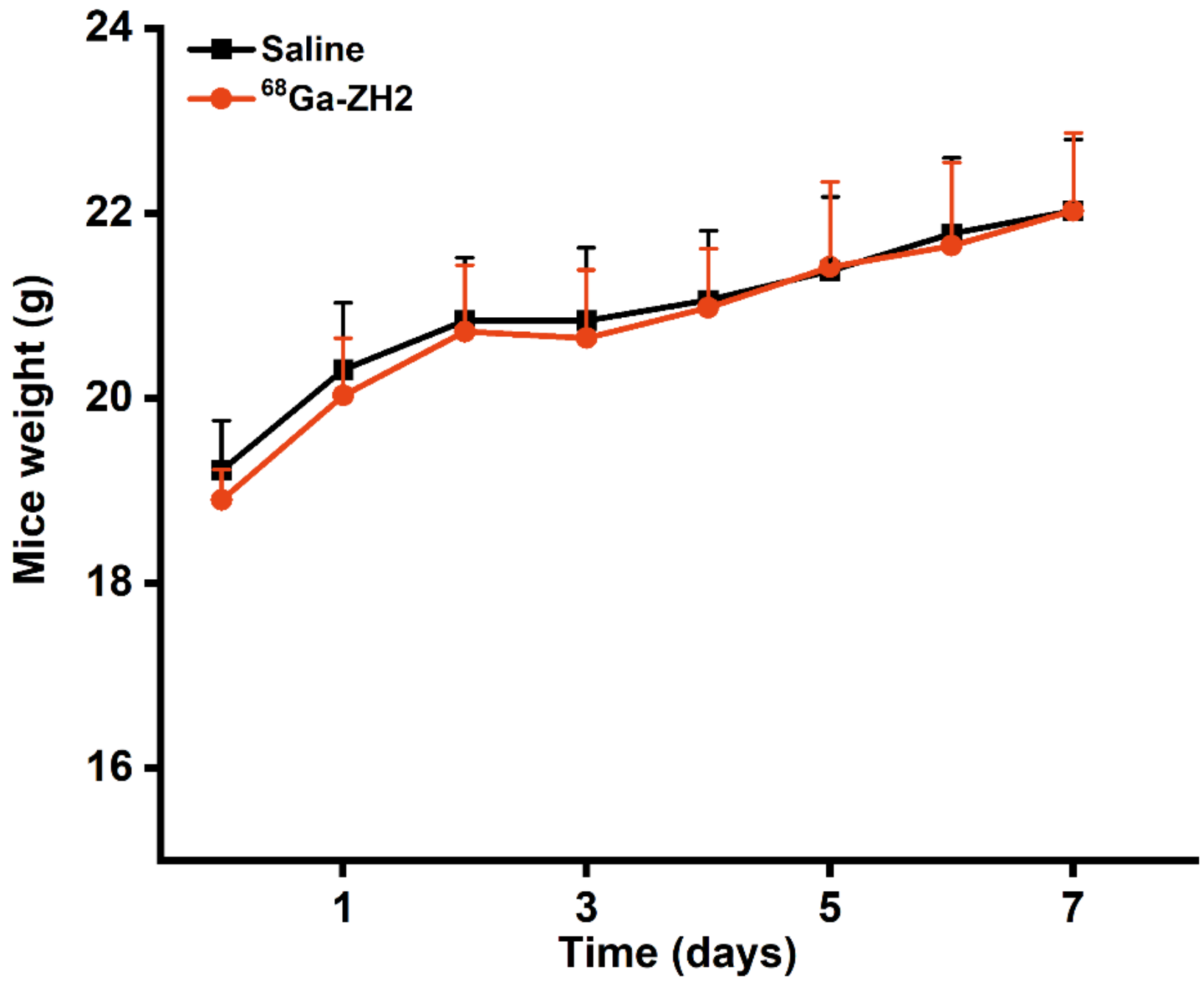

Figure S7. Toxicity test of ^{68}Ga-ZH2. Mice weight of control group and test group for both saline treatment group and ^{68}Ga-ZH2 treatment group (n = 10, mean ± SD).

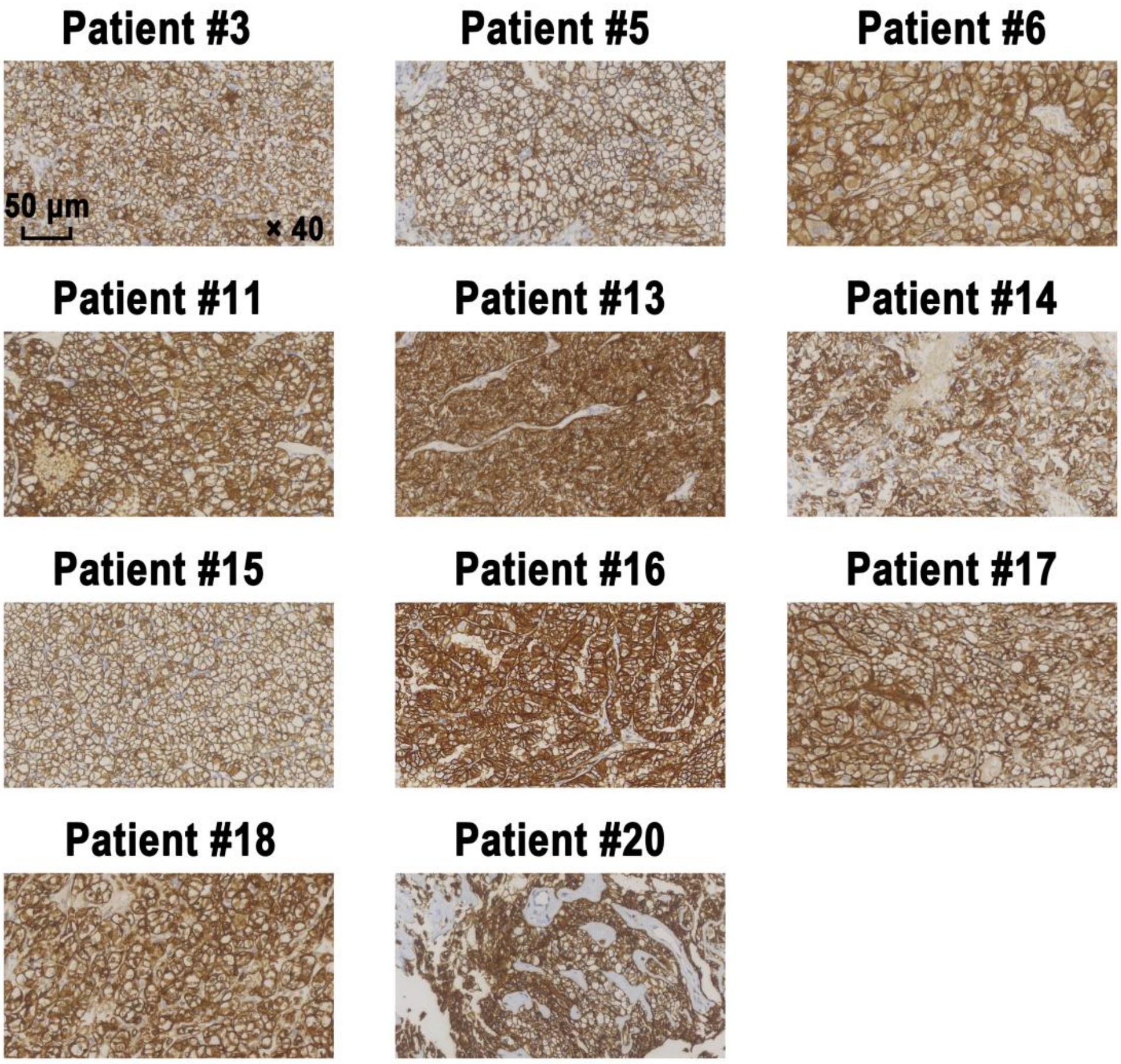

Figure S8. Immunohistochemical staining of patient tissue samples for CAIX. Original magnification, ×40, scale bar: 50 μm (for all panels).

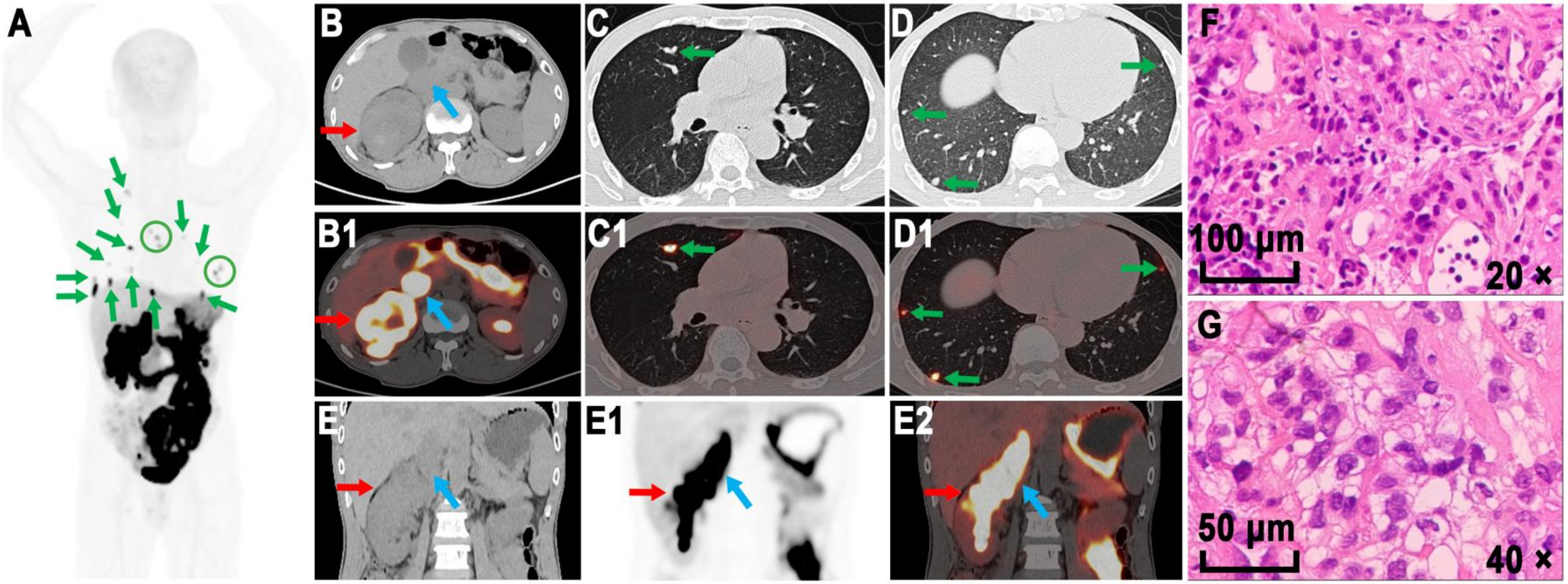

Figure S9. ^{68}Ga-ZH2 PET/CT in Patient #20 with Metastatic ccRCC. (A) Maximum intensity projection demonstrates widespread abnormal tracer uptake (green arrows and circles), indicating multifocal metastatic disease. Axial imaging of the abdomen (B, B1) shows the primary renal tumor (red arrows) and a right renal vein tumor thrombus (blue arrows), both with intense radiotracer uptake. Axial images of the lung (C, C1, D, D1) reveal multiple bilateral pulmonary nodules (green arrows), all exhibiting corresponding high ^{68}Ga-ZH2 avidity. Coronal CT, PET, and fused PET/CT images (E, E1, E2) further delineate the extent of the primary tumor (red arrows) and the cancer thrombus extending into the inferior vena cava (blue arrows). (F, G) Histopathological sections (20× and 40× magnification, respectively) from a biopsy of the right renal mass confirm ccRCC, showing tumor cells arranged predominantly in an acinar pattern with small, round nuclei and clear cytoplasm.

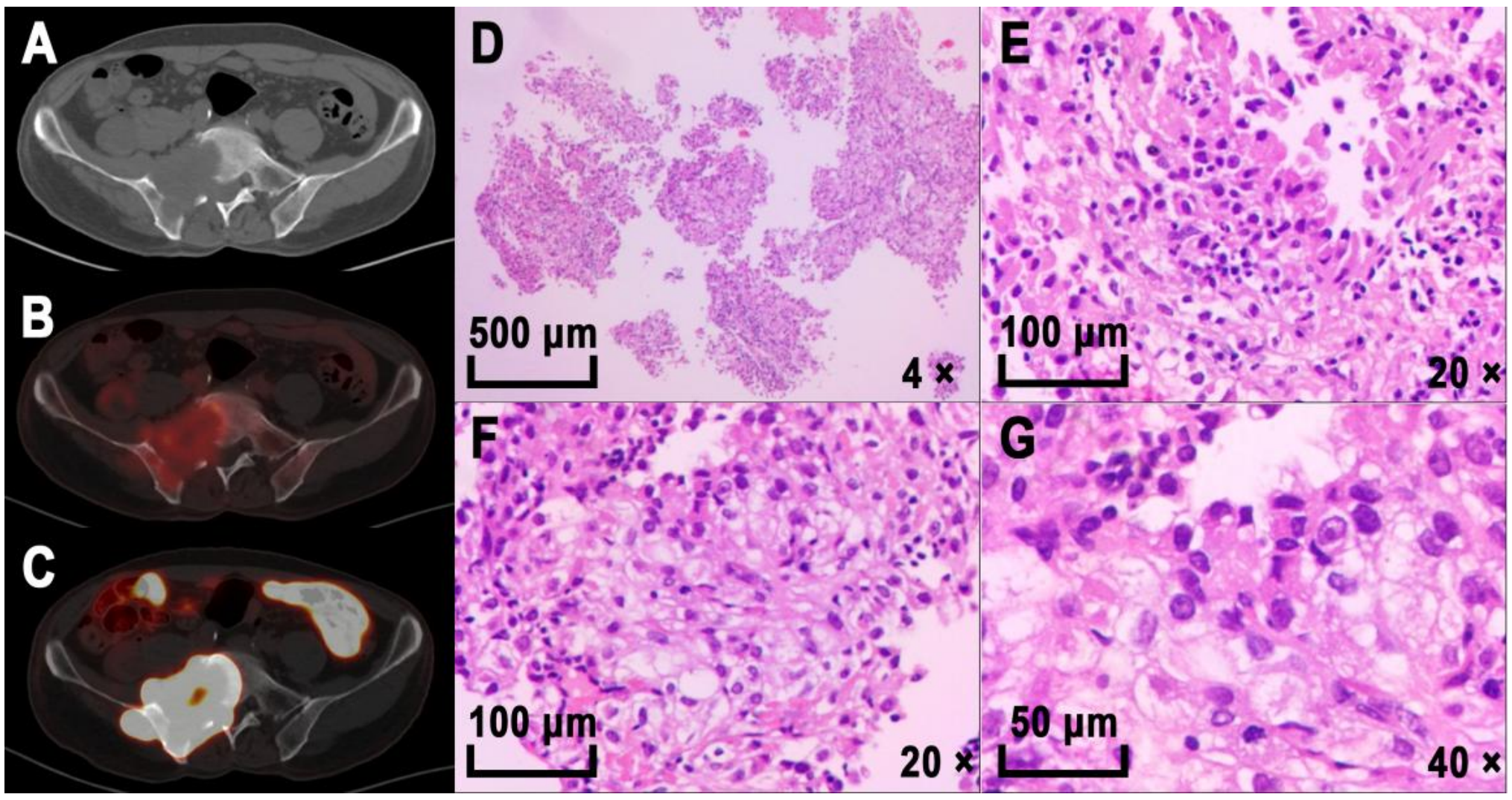

Figure S10. Imaging and histopathological findings of a metastatic lesion in the first sacral (S1) vertebra (Patient #4). (A) Axial CT demonstrates an osteolytic lesion in the ilium, the first sacral vertebra and the right pedicle. (B) ^{18}F-FDG PET/CT shows mild focal tracer uptake in the corresponding region. (C) ^{68}Ga-ZH2 PET/CT reveals intense radiotracer avidity. (D–G) Histopathological sections from a biopsy of the right pedicle of the S1 vertebra confirmed metastatic carcinoma with focal necrosis, consistent with renal cell carcinoma origin (hematoxylin and eosin stain; magnifications: ×4, ×20, and ×40).

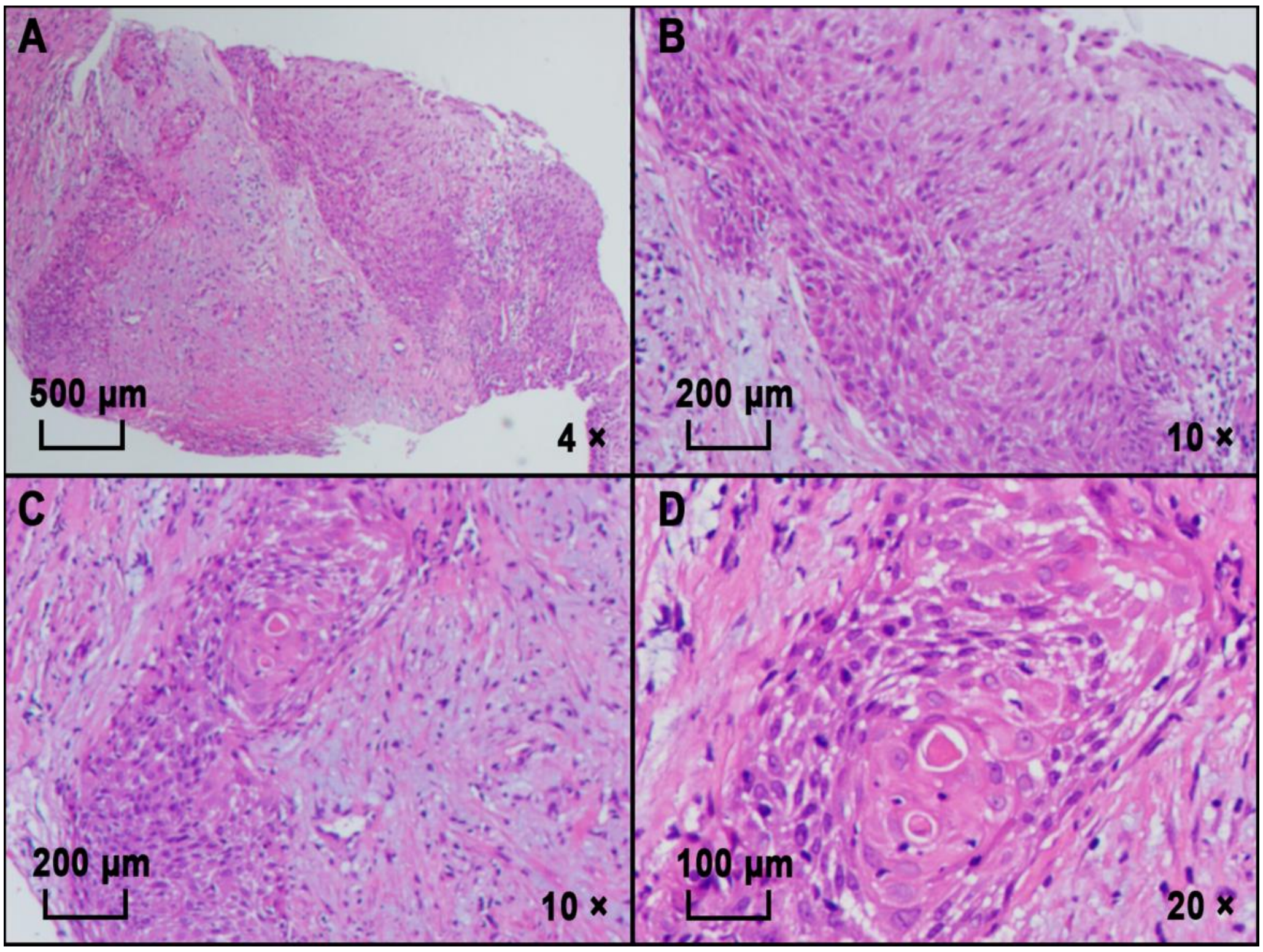

Figure S11. Histopathology of the resected left renal mass (Patient #19). (A~D) Microscopic examination revealed epithelioid cell clusters with focal squamous differentiation, mild cytological atypia, and locally infiltrative growth with evident keratinization. Admixed fibroadipose tissue, striated muscle, and parakeratotic squamous epithelium were also identified, confirming the diagnosis of keratinizing squamous cell carcinoma (hematoxylin and eosin stain, magnifications: ×4, ×10, and ×20).

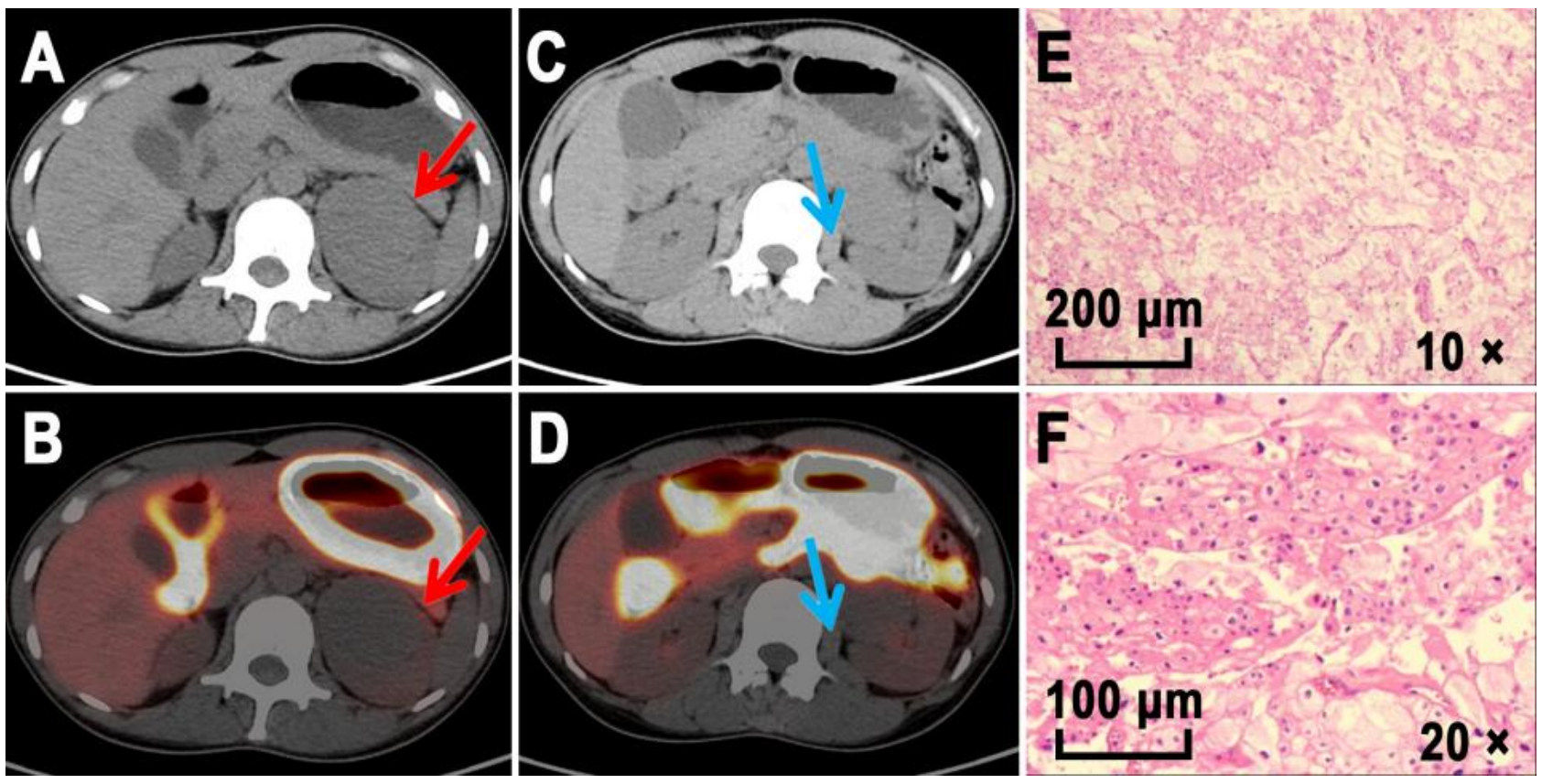

Figure S12. ^{68}Ga-ZH2 PET/CT in Patient #21 with chromophobe renal cell carcinoma. The primary renal tumor (A, B, red arrows) and retroperitoneal region (C, D, blue arrows) show no appreciable tracer uptake. (E, F) Histopathological examination of the surgically resected specimen confirmed the diagnosis of chromophobe renal cell carcinoma (hematoxylin and eosin stain, magnifications: ×10 and ×20).

3. Supplementary Tables

Table S1. Ex vivo biological distribution of ^{68}Ga-ZH1–3 and ^{68}Ga-DPI-4452 at 1 h.

Radioligand	^{68}Ga-ZH1	^{68}Ga-ZH2	^{68}Ga-ZH3	^{68}Ga-DPI-4452

**Organ**	**1 h** (% ID/g)	**1 h** (% ID/g)	**1 h** (% ID/g)	**1 h** (% ID/g)
Heart	0.90 ± 0.13	0.32 ± 0.10	0.31 ± 0.07	0.26 ± 0.04
Liver	1.26 ± 0.03	0.66 ± 0.13	0.60 ± 0.21	0.69 ± 0.07
Lung	1.94 ± 0.11	0.82 ± 0.22	0.94 ± 0.16	0.89 ± 0.24
Kidney	5.48 ± 0.39	3.25 ± 0.53	5.55 ± 0.44	4.48 ± 0.33
Spleen	0.80 ± 0.09	0.26 ± 0.05	0.21 ± 0.26	0.32 ± 0.08
Pancreas	0.86 ± 0.20	0.27 ± 0.05	1.19 ± 1.37	0.71 ± 0.31
Stomach	5.86 ± 0.95	5.51 ± 0.73	10.26 ± 0.89	11.11 ± 1.98
Bone	1.78 ± 0.20	0.73 ± 0.28	0.56 ± 0.20	0.99 ± 0.34
Muscle	0.72 ± 0.14	0.33 ± 0.13	0.34 ± 0.09	0.68 ± 0.34
Large intestine	1.29 ± 0.31	0.28 ± 0.09	0.41 ± 0.05	0.41 ± 0.12
Small intestine	1.65 ± 0.19	0.53 ± 0.11	1.59 ± 0.18	1.47 ± 0.13
Brain	0.34 ± 0.08	0.21 ± 0.09	0.19 ± 0.12	0.14 ± 0.09
Blood	1.20 ± 0.10	0.98 ± 0.28	0.64 ± 0.23	0.85 ± 0.16
Tumor	26.84 ± 1.70	41.84 ± 1.82	33.51 ± 2.23	33.32 ± 1.13

% ID/g, percentage injected dose per gram.

Table S2. The values of tumor to organ or tissue of ^{68}Ga-ZH2 and ^{68}Ga-DPI-4452 based on the ex vivo biodistribution data.

Radioligand	^{68}Ga-ZH2	^{68}Ga-DPI-4452
**Organ**	**ratio**	**ratio**
Liver	65.61 ± 11.11	49.08 ± 5.63

Lung	55.36 ± 16.99	39.71 ± 8.89
Kidney	13.02 ± 1.45	7.43 ± 0.55
Spleen	166.23 ± 26.59	116.75 ± 13.12
Pancreas	155.32 ± 18.01	62.07 ± 6.80
Bone	67.92 ± 30.09	29.23 ± 5.82
Muscle	156.56 ± 84.01	45.19 ± 12.93
Large intestine	135.87 ± 27.80	98.31 ± 12.20
Small intestine	83.75 ± 19.86	26.13 ± 6.38

Table S3. Ex vivo biological distribution of ^{177}Lu-ZH2 at 1, 4, 24, 72 and 168 h.

Radioligand	^{177}Lu-ZH2				
**Organ**	**1 h** (% ID/g)	**4 h** (% ID/g)	**24 h** (% ID/g)	**72 h** (% ID/g)	**168 h** (% ID/g)
Heart	0.08 ± 0.02	0.08 ± 0.06	0.03 ± 0.01	0.08 ± 0.08	0.02 ± 0.01
Liver	0.16 ± 0.03	0.16 ± 0.04	0.21 ± 0.13	0.20 ± 0.14	0.10 ± 0.04
Lung	0.17 ± 0.04	0.06 ± 0.01	0.04 ± 0.01	0.04 ± 0.02	0.06 ± 0.08
Kidney	13.56 ± 0.63	13.19 ± 1.73	3.87 ± 0.75	1.99 ± 1.20	0.54 ± 0.25

Spleen	0.08 ± 0.01	0.05 ± 0.02	0.07 ± 0.04	0.08 ± 0.07	0.06 ± 0.03
Pancreas	0.17 ± 0.04	0.05 ± 0.02	0.06 ± 0.02	0.05 ± 0.02	0.02 ± 0.02
Stomach	3.77 ± 1.15	0.53 ± 0.14	0.38 ± 0.11	0.21 ± 0.05	0.09 ± 0.02
Bone	0.33 ± 0.18	0.30 ± 0.17	0.14 ± 0.10	0.08 ± 0.10	0.10 ± 0.06
Muscle	0.11 ± 0.05	0.17 ± 0.03	0.03 ± 0.02	0.01 ± 0.01	0.02 ± 0.02
Large intestine	0.29 ± 0.08	0.23± 0.12	0.07 ± 0.02	0.05 ± 0.04	0.04 ± 0.03
Small intestine	0.40 ± 0.08	0.16 ± 0.07	0.06 ± 0.04	0.05 ± 0.02	0.05 ± 0.03
Brain	0.02 ± 0.02	0.02 ± 0.02	0.02 ± 0.02	0.004 ± 0.011	0.01 ± 0.01
Blood	0.24 ± 0.08	0.15 ± 0.16	0.07 ± 0.02	0.05 ± 0.02	0.02 ± 0.01
Tumor	83.51 ± 18.24	120.51 ± 15.15	90.74 ± 24.52	57.50 ± 8.55	19.79 ± 4.79

% ID/g, percentage injected dose per gram.

Table S4. Ex vivo biological distribution of ^{177}Lu-DPI-4452 at 1, 4, 24, 72 and 168 h.

Radioligand	^{177}Lu-DPI-4452				
**Organ**	**1 h** (% ID/g)	**4 h** (% ID/g)	**24 h** (% ID/g)	**72 h** (% ID/g)	**168 h** (% ID/g)
Heart	0.14 ± 0.05	0.05 ± 0.03	0.05 ± 0.02	0.05 ± 0.04	0.02 ± 0.02
Liver	0.36 ± 0.09	0.23 ± 0.02	0.32 ± 0.11	0.28 ± 0.18	0.18 ± 0.06
Lung	0.60 ± 0.14	0.09 ± 0.01	0.07 ± 0.03	0.10 ± 0.01	0.08 ± 0.08
Kidney	5.23 ± 1.01	3.22 ± 0.60	2.64 ± 0.45	0.61 ± 0.11	0.37 ± 0.08

Spleen	0.16 ± 0.06	0.11 ± 0.03	0.13 ± 0.04	0.12 ± 0.10	0.12 ± 0.04
Pancreas	0.57 ± 0.12	0.09 ± 0.02	0.05 ± 0.02	0.05 ± 0.01	0.03 ± 0.01
Stomach	8.72 ± 0.85	0.88 ± 0. 21	0.39 ± 0.08	0.27 ± 0.17	0.11 ± 0.04
Bone	0.25 ± 0.16	0.28 ± 0.07	0.26 ± 0.13	0.40 ± 0.09	0.36 ± 0.16
Muscle	0.21 ± 0.07	0.08 ± 0.06	0.15 ± 0.17	0.01 ± 0.02	0.01 ± 0.02
Large intestine	0.51 ± 0.19	0.23 ± 0.10	0.08 ± 0.03	0.08 ± 0.04	0.07 ± 0.04
Small intestine	0.82 ± 0.11	0.18 ± 0.08	0.11 ± 0.03	0.08 ± 0.05	0.06 ± 0.04
Brain	0.02 ± 0.01	0.01 ± 0.01	0.01 ± 0.01	0.02 ± 0.01	0.004 ± 0.004
Blood	0.48 ± 0.17	0.18 ± 0.09	0.06 ± 0.02	0.04 ± 0.03	0.02 ± 0.03
Tumor	77.63 ± 33.78	107.92 ± 18.86	89.31 ± 19.54	57.58 ± 6.24	18.76 ± 2.83

% ID/g, percentage injected dose per gram.

Table S5. The values of AUC and AUC ratio of tumor to organ or tissue of ^{177}Lu-ZH2 and ^{177}Lu-DPI-4452 based on the ex vivo biodistribution data.

Radioligand	^{177}Lu-ZH2		^{177}Lu-DPI-4452	
**Organ**	**AUC**((%ID/g)·h)	**AUC ratio**	**AUC**((%ID/g)·h)	**AUC ratio**
Heart	8.58 ± 5.30	1128.51	7.24 ± 2.52	1304.23
Liver	28.62 ± 10.05	338.43	42.60 ± 12.79	221.60
Lung	17.00 ± 16.13	569.76	22.35 ± 11.49	422.37
Kidney	481.30 ± 86.40	20.12	183.90 ± 25.24	51.33

Spleen	11.91 ± 4.73	813.27	19.99 ± 6.42	472.24
Pancreas	7.31 ± 1.68	1324.67	8.60 ± 1.13	1098.19
Stomach	43.68 ± 5.40	221.75	61.09 ± 11.20	154.53
Bone	19.87 ± 7.97	487.47	58.56 ± 11.25	161.20
Muscle	4.52 ± 1.23	2144.34	7.19 ± 5.22	1313.30
Large intestine	11.70 ± 3.34	827.86	14.92 ± 3.65	632.71
Small intestine	10.51 ± 2.51	921.60	16.09 ± 4.02	586.70
Brain	1.81 ± 1.19	5357.30	1.90 ± 0.59	4965.81
Blood	9.02 ± 2.38	1073.84	8.37 ± 2.92	1127.43
Tumor	9686.00 ± 1064.00	1.00	9440.00 ± 810.20	1.00

AUC, areas under the curve.

Table S6. The non-tumor organs and primary tumor uptakes (SUVmax) of ^{68}Ga-ZH2 in the patients with renal clear cell carcinoma.

Patient no.	Heart	Liver	Lung	Kidney	Spleen	Pancreas	Stomach	Bone	Muscle	Large Intestine	Small Intestine	Brain	Aortic Arch	Primary Tumor
1	1.1	4.3	0.5	4.1	1.5	23.1	16.3	1.2	0.5	2.1	21.6	0.03	1.7	-
2	1.6	3.4	0.5	3.6	1	3.2	13.8	1.2	0.3	3.2	13.5	0.3	1.5	14.1
3	0.6	3.4	0.4	1.8	0.9	16	90.7	0.2	0.3	0.9	39.9	0.08	1.1	244
4	0.3	3.9	0.1	0.7	0.4	5.2	50.6	0.1	0.1	0.1	15.2	0.04	0.5	186.6
5	0.7	4.4	0.3	3.3	0.4	6.8	58.2	0.2	0.2	0.8	37.6	0.04	0.8	81.1
6	0.5	5.6	0.3	2.4	0.5	7.7	50.8	0.1	0.4	0.1	16.6	0.04	0.9	47.8
7	0.7	2.7	0.3	3.2	0.7	2.9	18.4	0.2	0.4	0.8	30.1	0.05	1.1	-
8	0.7	3.8	0.3	1.8	0.6	9.9	40.9	0.2	0.2	0.9	18.9	0.05	0.9	65.1
9	0.5	2.4	0.3	2.1	0.8	4.2	35.7	0.2	0.2	1	15.3	0.05	0.9	169.2
10	0.4	2.3	0.2	1.3	0.3	3.8	72	0.2	0.2	0.8	28.4	0.02	0.5	84.3
11	0.6	2	0.3	1.9	0.4	3.3	25	0.2	0.2	0.5	14.8	0.02	0.7	178.1
12	0.6	1.3	0.3	2.2	0.4	4.1	30.5	0.3	0.2	0.3	19.7	0.04	1	102.3
13	0.5	2.5	0.2	2	0.4	24.2	68.7	0.2	0.2	0.8	25.7	0.04	0.7	110.6
14	0.7	1.8	0.3	1.6	0.6	3.1	34.7	0.3	0.1	0.6	32.1	0.03	0.7	96.7
15	0.9	3.9	0.3	2.7	0.6	7.1	32.6	0.3	0.2	0.9	23.9	0.04	0.9	223.2
16	0.6	3.5	0.2	2.4	0.5	7	62.3	0.2	0.2	0.6	27.9	0.02	0.8	123.5
17	0.8	3.5	0.4	2.1	0.6	17.1	56.7	0.2	0.4	1.1	37.2	0.03	1.4	105.9
18	0.4	4.2	0.2	1.9	0.7	99.5	13.7	0.1	0.3	0.6	15.9	0.02	0.9	195.9

SUVmax, maximum standardized uptake value.
